# Replacing a Cereblon Ligand by a DDB1 and CUL4 Associated Factor 11 (DCAF11) Recruiter Converts a Selective Histone Deacetylase 6 PROTAC into a Pan‐Degrader

**DOI:** 10.1002/cmdc.202500035

**Published:** 2025-03-24

**Authors:** Felix Feller, Heiko Weber, Martina Miranda, Irina Honin, Maria Hanl, Finn K. Hansen

**Affiliations:** ^1^ Department of Pharmaceutical and Cell Biological Chemistry Pharmaceutical Institute University of Bonn An der Immenburg 4 53121 Bonn Germany

**Keywords:** DDB1- and CUL4-associated factor 11 (DCAF11), Histone deacetylase (HDAC), Proteolysis targeting chimeras (PROTACs), Solid-phase synthesis, Targeted Protein Degradation (TPD)

## Abstract

Proteolysis‐targeting chimeras (PROTACs) have recently gained popularity as targeted protein degradation (TPD) promises to overcome the limitations of occupancy‐driven pharmacology. However, most degraders rely on a small number of E3 ligases. In this study, we present the first‐in‐class histone deacetylase (HDAC) PROTACs recruiting the DDB1‐ and CUL4‐ associated factor 11 (DCAF11). We established a synthesis route entirely on solid‐phase to prepare a set of eleven degraders. The long and flexible spacer bearing **FF2039** (**1j**) showed significant HDAC1 and 6 degradation in combination with cytotoxicity against the multiple myeloma cell line MM.1S. Further investigations revealed that **1j** was also able to degrade HDAC isoforms of class I, IIa and IIb. Compared to our previously published cereblon‐recruiting HDAC6 selective PROTAC **A6**, we succesfully transformed the selective degrader into a pan‐HDAC degrader by switching the recruited E3 ligase. A detailed profiling of the anticancer properties of **1j** demonstrated its significant antiproliferative activity against both hematological and solid cancer cell lines, driven by cell cycle arrest and apoptosis induction.

## Graphical abstract

This study introduced DCAF11 as an E3 ligase for PROTAC‐mediated histone deacetylase (HDAC) degradation. By replacing cereblon with DCAF11 as recruited E3 ligase, a selective HDAC6 degrader (**A6**) was transformed into pan‐HDAC degraders. The lead compound, **1j** (**FF2039**), potently degraded HDACs from classes I, IIa, and IIb, showing strong antiproliferative effects on multiple myeloma cells (MM.1S) *via* cell cycle arrest and apoptosis induction.

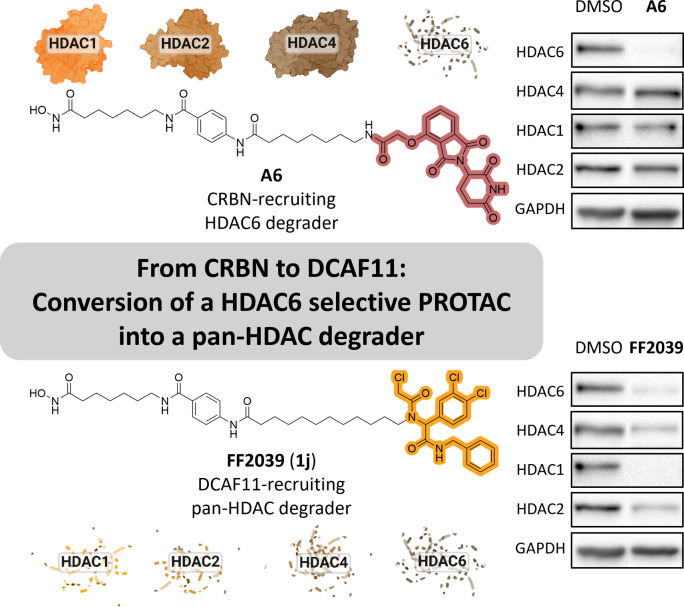

## Introduction

Cancer is the cause of nearly 10 million deaths per year, making it one of humanity's most significant disease burdens, particularly in western societies.^[1]^ It arises from a variety of factors, including genetic mutations and epigenetic alterations, the latter involving chromatin modifications without changes to the DNA sequence.^[2]^ Chromatin remodeling, like ϵ‐lysine acetylation of histones, is tightly regulated, as it is responsible for modulation of transcription, DNA repair, replication, and condensation.^[3]^ To reverse the acetylation, histone deacetylases (HDACs) hydrolyze the amide bond, restoring the positive charge of the lysine and resulting in a more compact chromatin.^[4]^

HDACs can be distinguished into four classes: Class I (HDAC1‐3 and 8), class IIa (HDAC4, 5, 7 and 9), class IIb (HDAC6 and 10), and class IV (HDAC11) are zinc‐dependent deacylases. Depending on the isoform, they are primarily localized in the nucleus or in the cytoplasm and some contribute to multi‐protein complexes. In addition, HDACs act on multiple substrates beyond histones and are involved in the hydrolysis of more than just acetyl groups.[^5^, ^6^] The up‐regulation and high expression of different HDAC isoforms are associated with poor prognosis in cancers such as multiple myeloma and acute myeloid leukemia.[^7^, ^8^] Therefore, HDAC inhibition is a promising strategy for tumor therapy. Numerous studies have demonstrated that HDAC inhibitors (HDACi) reduce angiogenesis, cell migration, proliferation, and resistance to chemotherapy. Furthermore, inhibition of HDACs promotes apoptosis and enhances cell differentiation.[^6^, ^9^] U.S. Food and Drug Administration (FDA) or European Medicines Agency (EMA) approved HDACi, such as vorinostat, belinostat, and romidepsin, for the treatment of T‐cell lymphoma, panobinostat for the treatment of multiple myeloma, and givinostat for treating Duchenne muscular dystrophy.[^10^, ^11^] In addition, the National Medical Product Administration of China approved tucidinostat for treating peripheral T‐cell lymphoma and hormone receptor positive breast cancer.[^12^, ^13^]

A promising alternative to conventional occupancy‐driven inhibitors is targeted protein degradation (TPD) for example by molecular glues or proteolysis‐targeting chimeras (PROTACs). In this approach, an E3 ligase is hijacked by a molecular glue or PROTAC to polyubiquitinate a protein of interest (POI), leading to its degradation. In Cullin RING E3 ligases, the largest group of E3 ligases, the E3 ligase complex works alongside an ubiquitin‐loaded E2 enzyme, which transfers ubiquitin to the substrate or POI. This leads to subsequent degradation by the ubiquitin‐proteasome system (UPS).[^14^, ^15^] This catalytic mode of action provides significant advantages, such as extended pharmacological effects, doses reduction, and potentially minimizing adverse effects.[^16^, ^17^] Importantly, degraders can overcome cancer resistance mechanisms, such as target amplification or overexpression, through their catalytic activity.^[18]^ Additionally, they can counteract resistance caused by altered ligand binding sites, as even weak binders can still enable efficient degradation.^[19]^

While molecular glues identification is often serendipitous, PROTACs can be designed more rationally. However, due to recent discoveries in degrader development it becomes more and more difficult to differentiate between molecular glues and PROTACs. This becomes particular evident in a series of linker‐less PROTACs that combine a POI‐warhead with a covalent handle for a specific E3 ligase. These compounds, termed monovalent molecular glues or linker‐less PROTACs, challenge traditional classifications.[^20^, ^21^]

**Figure 1:**
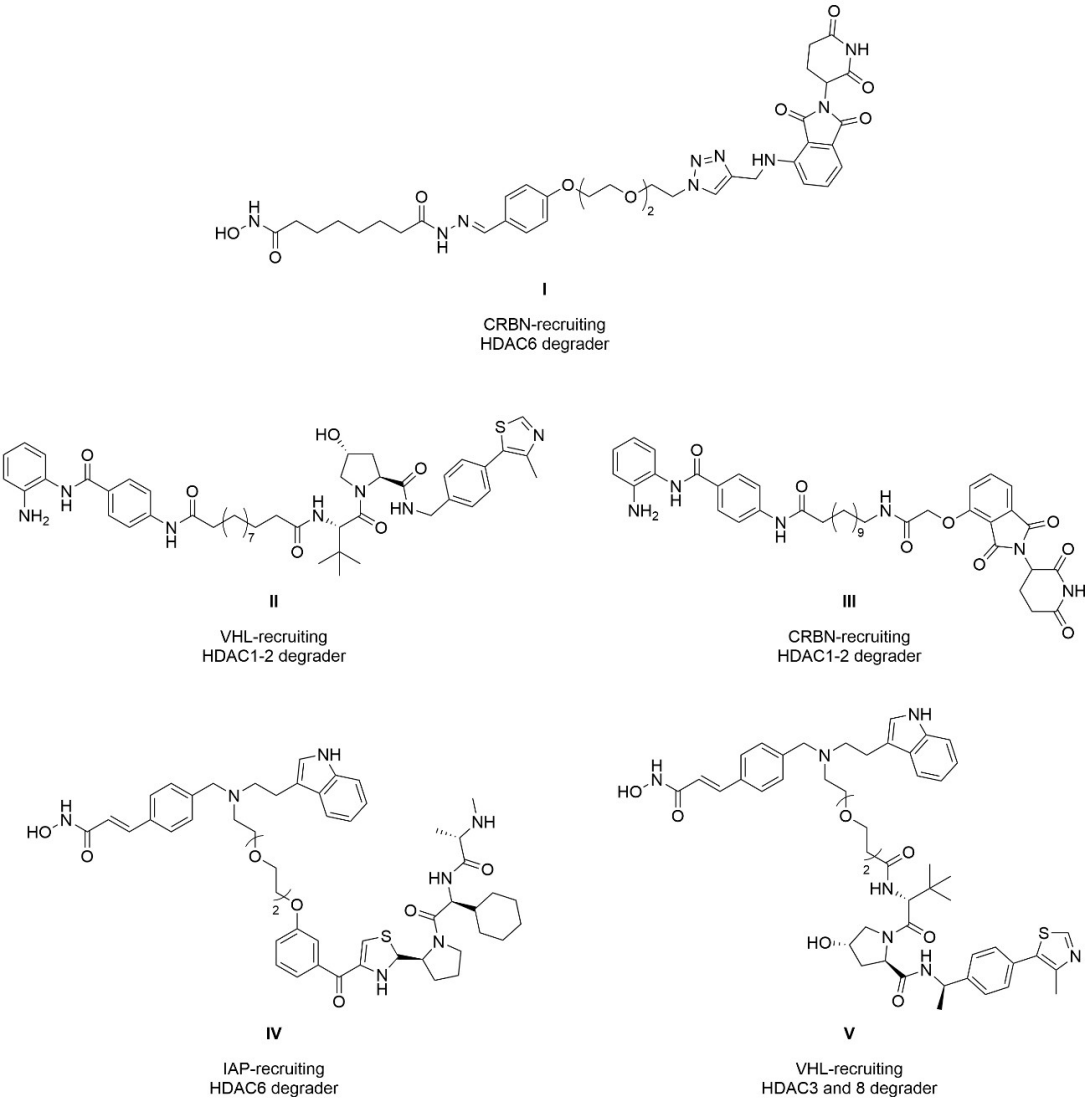
Comparison of HDAC PROTACs recruiting different E3 ligases. PROTAC **I** ^[22]^ was the first utilization of CRBN for HDAC degradation, while PROTAC **II** ^[28]^ represents the first VHL‐based HDAC degrader. PROTAC **III** ^[28]^ showed similar degradation selectivity as PROTAC **II**, but to a lesser extent by switching back to a CRBN recruiter. The first successful utilization of IAP for targeted HDAC degradation was PROTAC** IV** ^[29]^ and changing the E3 ligase to VHL, represented by PROTAC **V**,^[29]^ resulted in a switch of degraded isoforms.

PROTACs for targeted degradation of HDACs were introduced in 2018, and since then, over 100 HDAC PROTACs have been published.[^22^, ^23^, ^24^, ^25^, ^26^] Despite the use of many different HDAC ligands, only three E3 ligases have been employed according to PROTAC‐DB 3.0: Cereblon (CRBN), Von Hippel‐Lindau (VHL), and the inhibitor of apoptosis protein (IAP).^[27]^ The first PROTAC for targeted degradation of an HDAC was the crebinostat‐derived degrader **I** (Figure 1). It used pomalidomide for CRBN recruitment and despite PROTAC **I** contains a pan‐HDAC ligand, only HDAC6 was degraded.^[22]^ PROTAC **II** and **IV** are representatives of the first successful recruitment of VHL and IAP, respectively. PROTAC **III** contains a benzamide for targeting class I HDACs, and the switch to a CRBN recruiter shows similar degradation selectivity as **II**, but to a lesser extent.^[28]^ The E3 ligase switch from **IV** to **V** yielded a different result: Both contain the pan‐HDAC inhibitor dacinostat and a polyethylene glycol (PEG) spacer, but the IAP‐recruiting **IV** resulted in HDAC6 degradation, while **V** degraded HDAC3 and 8 by VHL recruitment.^[29]^ With more than 600 E3 ligases encoded in the human genome, there is a significant need to expand the number of utilized E3 ligases for HDAC degradation.^[30]^ Furthermore, unlocking specific E3 ligases with dedicated tissue or cell‐type specificity holds great potential for achieving degradation in the desired tissue. Likewise, targeting tumor‐ or disease‐enriched E3 ligase could enable the development of more refined and selective degraders.[^31^, ^32^] Expanding the use of new E3 ligases can also help to target new tumor entities; for example, it has been shown that the sensitivity pattern of tested tumor cell lines to PROTACs changes by switching from CRBN to VHL.^[33]^ However, ligands for both E3 s come with some limitations, as thalidomide is associated with teratogenicity and stability issues, while the VHL ligands increase molecular weight and topological polar surface area, which can be challenging for oral bioavailability.^[17]^ Using E3 ligases which degrade tumor suppressor‐proteins can have additional anticancer effects. For example, mouse double minute 2 homolog (MDM2) recruitment for TPD leads to a stabilization of p53 and p21 is substrate of the E3 ligase DCAF11.[^34^, ^35^]

Lately, the range of E3 ligases used in TPD has expanded. For example, ligands for Fem‐1 homolog B (FEM1B),^[36]^ Ring Finger Protein 4 (RNF4),^[37]^ Ring Finger Protein 114 (RNF114),^[38]^ DDB1‐ and CUL4‐associated factor 11 (DCAF11),[^39^, ^40^, ^41^, ^42^, ^43^] and DDB1‐ and CUL4‐associated factor 16 (DCAF16)^[44]^ have been utilized in TPD strategies. These ligands share an electrophilic warhead to engage the E3 ligase, resulting in a pseudo‐binary complex of E3 ligase, covalently bound PROTAC, and POI. This results in simpler kinetics, as the covalent construct of E3 ligase and PROTAC only needs to recruit a new POI molecule for polyubiquitination.^[45]^

**Figure 2:**
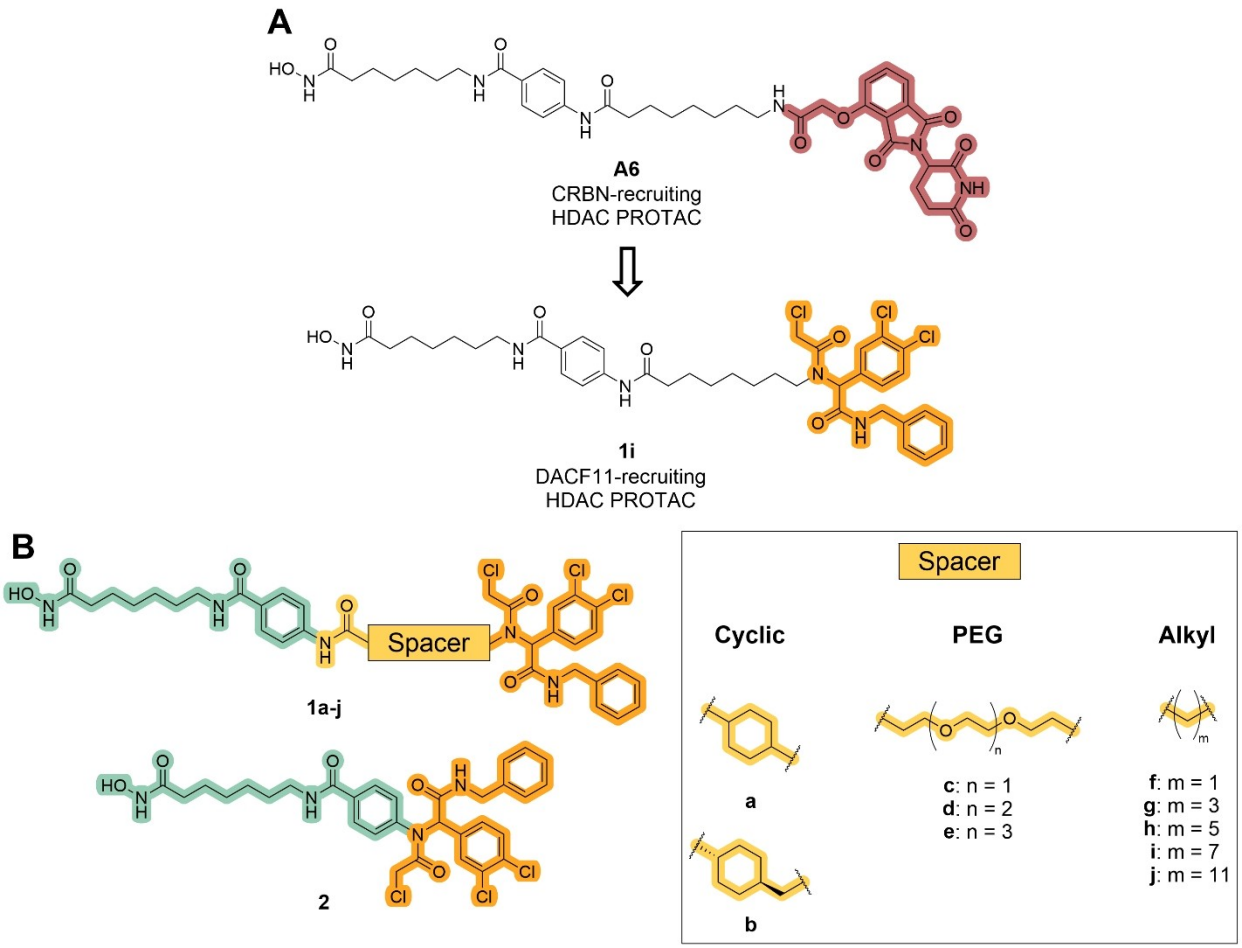
Design of DCAF11‐recruiting HDAC PROTACs. (A) Switch from the CRBN‐recruiting thalidomide‐based E3 ligase ligand (red) of **A6** to the DCAF11‐recruiting ligand (orange). (B) The DCAF11‐based HDAC PROTACs (**1a‐j**) consist of three parts: The vorinostat‐like HDAC ligand (green) and the DCAF11 ligand (orange) are connected by a broad spectrum of spacers (yellow). The spacer‐less compound **2** consist only of the HDAC and DCAF11 ligand.

In this work, we utilized the Ugi four component reaction (U‐4CR)‐derived DCAF11 ligand of Zhang et al.,^[39]^ because of its promising degradation results and fast accessibility due to the U‐4CR. Based on our previously published HDAC6 degrader **A6**, we started to design the DCAF11‐recruiting PROTACs (Figure 2).^[46]^ In our previous studies, we developed a highly modular approach for the solid‐phase synthesis of PROTACs and extended this approach to the synthesis of hydrophobically tagged HDAC inhibitors.[^46^, ^47^, ^48^] Adapting the U‐4CR to the solid‐phase conditions allowed us to synthesize a set of eleven PROTACs entirely on resin. The subsequent biological evaluation revealed significant degradation of HDAC1, correlating with cytotoxic effects in the multiple myeloma cell line MM.1S. Notably, further investigation of compounds **1j** and **2**, which share the POI ligand with **A6** but differ in linker length and E3 ligase ligand, revealed enhanced degradation capabilities and both compounds induced pan‐HDAC degradation across all isoforms tested. This effect was accompanied by increased cell cycle arrest, apoptosis induction, and long‐term antiproliferative activity.

## Results and Discussion

### Design and Synthesis of DCAF11‐Recruiting HDAC PROTACs

The design of our PROTACs was based on the best degrader of our previous HDAC degrader study, **A6**,^[46]^ which combines a vorinostat‐like HDAC inhibitor and a thalidomide‐based CRBN ligand, fused by a C7 aliphatic spacer (Figure 2A). Although vorinostat is a pan‐HDAC inhibitor, **A6** specifically targeted HDAC6 for degradation. The aim of this study was to investigate how switching the E3 ligase affects the HDAC degradation selectivity profile.

**Scheme 1:**
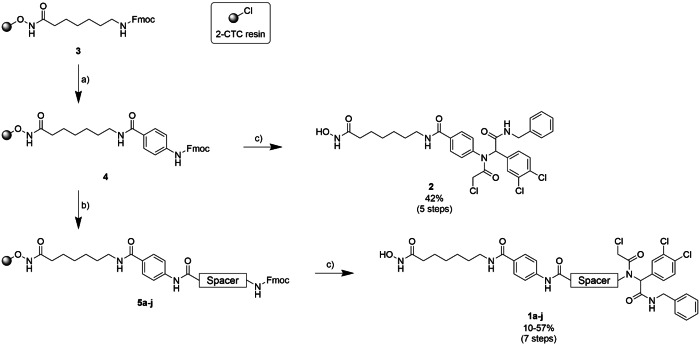
Solid‐phase synthesis of DCAF11‐recruiting HDAC PROTACs. *Reagents and conditions*: a) (i) 20% Piperidine, DMF, rt, 2×5 min, (ii) Fmoc‐4‐aminobenzoic acid, HATU, HOBt⋅H_2_O, DIPEA, DMF, rt, 18 h; b) (i) 20% piperidine, DMF, rt, 2×5 min, (ii) Fmoc‐NH‐spacer‐COOH, HATU, HOBt⋅H_2_O, DIPEA, DMF, rt, 18 h; c) (i) 20% piperidine, DMF, rt, 2×5 min, (ii) 3,4‐dichlorobenzaldehyde, 2‐chloroacetic acid, benzyl isocyanide, DMF/MeOH (1/1 *v*/*v*), rt, 72 h, (iii) 5% TFA, 5% triisopropylsilane, CH_2_Cl_2_, rt, 1 h.

To convert the CRBN‐utilizing PROTAC into one that recruits DCAF11, we replaced the thalidomide‐based ligand (red, **A6**) with a DCAF11 ligand (orange, **1i**, Figure 2A). We retained the vorinostat‐like pan‐HDACi ligand and C7 linker, to trace back any possible effects to the exchange of the E3 ligase recruiter. The initial PROTAC **1i**, which represents the direct DCAF11‐recruiting analog of **A6**, was complemented by a set of nine degraders, bearing a broad range of different spacers to introduce diversity in regards to chain length, lipophilicity, and rigidity. In detail, two short and rigid cyclic spacers (**1a‐b**, Figure 2B), three more flexible PEG spacers (**1c‐e**), and some alkyl spacers ranging from C1‐C11 (**1f‐j**) were selected. As mentioned before, the line between PROTACs and molecular glues is becoming increasingly blurred,[^20^, ^49^] we also designed a compound that can be considered as a spacer‐less PROTAC (**2**). In total, we decided to synthesize eleven PROTACs to study the effect of DCAF11 recruitment on HDAC degradation. The synthesis of the DCAF11‐recruiting PROTACs was completely carried out on solid support and is shown in Scheme 1. For resin modification, loading determination, and amide coupling reactions, our previously published protocols were used.[^46^, ^47^, ^48^] Specifically, the commercially available 2‐chlorotrityl chloride (2‐CTC) resin was modified with *N*‐hydroxyphthalimide to immobilize the hydroxylamine as the precursor for the hydroxamic acid on the resin (not shown). The subsequent hydrazine monohydrate‐mediated deprotection released the resin‐bound hydroxylamine, enabling its coupling with Fmoc‐7‐aminoheptanoic acid, which was facilitated by HATU and HOBt. This step formed the zinc‐binding group and linker of the resin‐bound HDAC inhibitor **3**. After Fmoc‐deprotection of the precursor, Fmoc‐4‐aminobenzoic acid was used to complete the HDACi **4**. The last step before the introduction of the DCAF11 ligand was the attachment of various spacers, which was performed in the same manner as previous couplings steps to produce **5a‐j**. The precursors **4** and **5a‐j** were then used to install the DCAF11 ligand by an on resin U‐4CR. To this end, 3,4‐dichlorobenzaldehyde, 2‐chloroacetic acid, and benzyl isocyanide were used in the U‐4CR. This synthetic approach enabled the introduction of the DCAF11 ligand in one final step. Finally, all compounds were cleaved from the resin and purified by preparative HPLC to >95% purity.

### Degradation and Antiproliferative Activity of DCAF11‐Recruiting PROTACs

**Figure 3:**
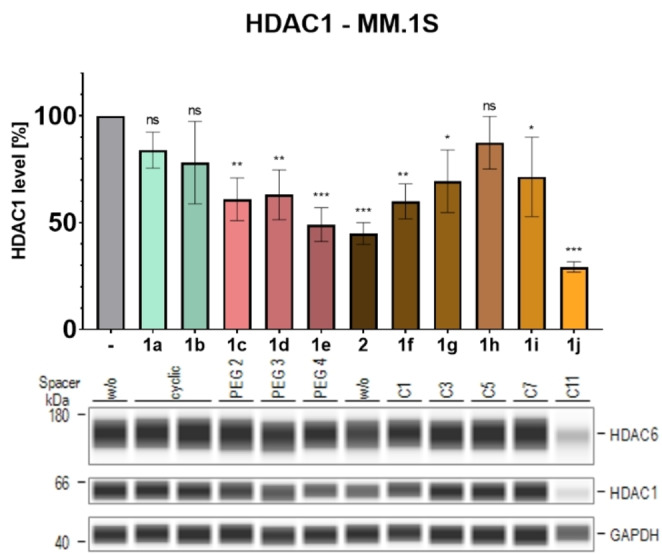
Initial degradation screening of HDAC1 and 6 by DCAF11‐recruiting PROTACs by Simple Western™ immunoassay analysis of MM.1S cell lysates. MM.1S cells were treated with the indicated compound (10 μM) or vehicle (DMSO) for 24 h. Top: Quantification of HDAC1 levels, presented as mean ± standard deviation of n=3 biological replicates; Significance compared to vehicle: ns=p≥0.05; *=p≤0.05, **=p≤0.01, ***=p≤0.001. Bottom: Representative images from a total of n=3 biological replicates, labeled with spacer type; w/o: without spacer.

To investigate the HDAC degradation capability of the novel DCAF11‐recruiting PROTACs, we treated the multiple myeloma cell line MM.1S with the compounds and performed automated Simple Western™ immunoassays. According to DepMap, MM.1S cells show similar baseline expression levels of DCAF11 and CRBN (5.02 and 4.9 (log2) transcription per million respectively).^[50]^ We first examined the effects on HDAC1 and 6 protein levels, as they represent members of the most studied HDAC classes (I and IIb) and are mainly located in opposite cellular compartments (nucleus and cytoplasm). Figure 3 summarizes the analyzed HDAC1 and 6 levels: HDAC1 was not degraded by PROTACs bearing the cyclic spacers, but the longer and more polar PEG spacer‐containing PROTACs showed a significant reduction of up to 51% (**1e**). The alkyl spacer‐based compounds revealed some unexpected results: while the C5 spacer (compound **1h**) did not reduce HDAC1 levels, PROTACs with both shorter and longer spacers showed a trend toward significant HDAC1 degradation. Interestingly, also the spacer‐less degrader **2** demonstrated significant HDAC1 degradation. The most pronounced reduction of HDAC1 levels was detected for spacer‐less PROTAC **2** (55%) and the longest C11 spacer bearing PROTAC **1j** (71%). In addition, these two PROTACs were also capable of achieving significant reduction of HDAC6 protein levels, whereas all the other compounds showed no significant effects on HDAC6 protein abundance.

**Figure 4:**
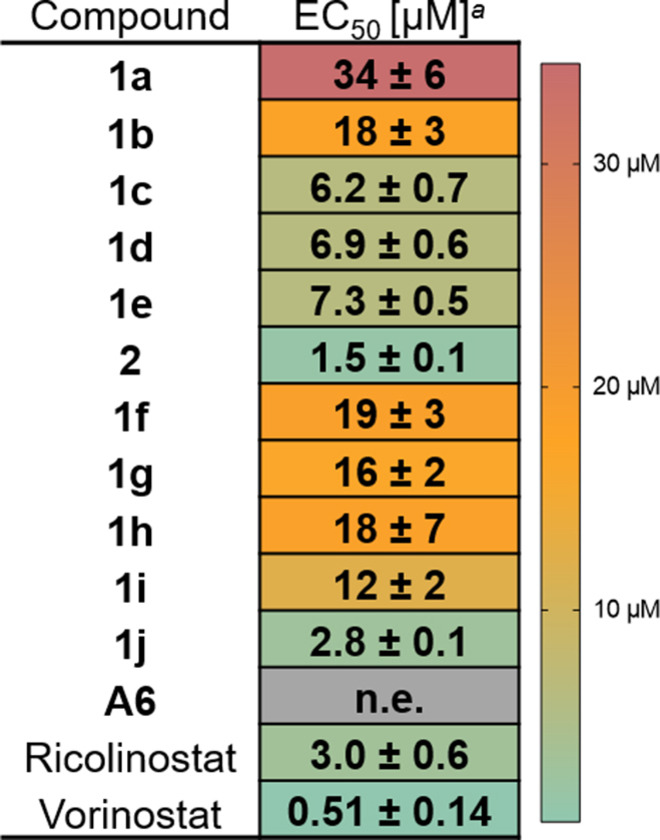
Antiproliferative activity of DCAF11‐recruiting PROTACs. ^*a*^Mean ± standard deviation of EC_50_ of three independent experiments, each in duplicates; n.e.: no effect (≤25% effect up to 50 μM). Antiproliferative activity of DCAF11‐recruiting PROTACs in MM.1S cells after 72 h.

Encouraged by the promising reductions in HDAC1 and HDAC6 levels, we further investigated the phenotypic effects of our DCAF11‐recruiting PROTACs on cancer cells. Accordingly, we performed a cell viability assay on multiple myeloma MM.1S cells after treatment with the indicated compounds. The results are presented in Figure 4. The rigid cyclic spacer‐bearing PROTACs (**1a**, **1b**) demonstrated diminished antiproliferative activity, whereas the spacer‐less (**2**: EC_50_=1.5 μM) and the longest alkyl spacer (**1j**: EC_50_=2.8 μM) yielded pronounced degradation of both HDAC1 and 6. In addition, **2** and **1j** displayed superior antiproliferative activity in MM.1S cells compared to ricolinostat, an HDACi currently under clinical investigation.^[51]^ Furthermore, we investigated the contribution of the DCAF11 ligand to the antiproliferative activity of the PROTACs. To this end, we synthesized the *n*‐propyl‐substituted DCAF11 warhead **6** (see Scheme S1, Supporting Information) and tested a 1 : 1 combination of vorinostat and **6** in a cell viability assay. Since the EC_50_ of the combination treatment was similar to that of vorinostat alone, the DCAF11 ligand seems not to add significant cytotoxicity (Figure S1, Supporting Information). In contrast, the thalidomide‐based PROTAC **A6** showed no effect on the viability of MM.1S cells, which is in line with the literature, where there was little to no effect by **A6** depending on the cell line.[^46^, ^52^]

Notably, the antiproliferative activity of **2** and **1j** is reduce compared to the parent HDACi vorinostat by a factor of ~3 – 6, which is comparable to the previously published FEM1B‐recruiting PROTACs sharing the same POI ligand.^[52]^ To investigate this reduced antiproliferative activity in comparison to vorinostat, we tested cell permeability using a cellular HDAC inhibition assay in the presence and absence of IGEPAL as a permeabilization agent. Cellular permeability does not appear to be an issue for the PROTACs, as cellular HDAC inhibition is not dependent on the addition of IGEPAL (Figure S2, Supporting Information). Finally, aqueous stability was identified as a potential source of reduced antiproliferative activity. Vorinostat remained intact in DPBS buffer for 72 hours, whereas PROTAC **2** decomposed by 31% over the same period. During the first 24 hours, PROTAC **1j** showed similar stability to **2**. However, after 72 hours, **1j** showed a degradation profile that was comparable to that of **A6**, with a reduction in content of more than 90% (Figure S3, Supporting Information).

### Target Engagement and HDAC Degradation Selectivity Profile of 2 and 1j

For further investigations, we selected the two most effective HDAC1 degraders from this set (**2** and **1j**), as they also demonstrated the highest antiproliferative activity. As target engagement is crucial for targeted protein degradation, we next investigated the HDAC inhibition efficiency of the PROTAC hits. To distinguish between their effects on the various isoforms, we evaluated the inhibitory activity on representative isoforms from different HDAC classes, namely HDAC1, 2, 4 and 6 (Table 1). Compound **2** demonstrated comparable inhibition results as **A6**. However, **1j**, the most potent degrader, exhibited comparatively weaker inhibition of all tested isoforms. The half‐maximal inhibitory concentration (IC_50_) is consistently ~4‐8 times higher than that of **2**

**Table 1:**
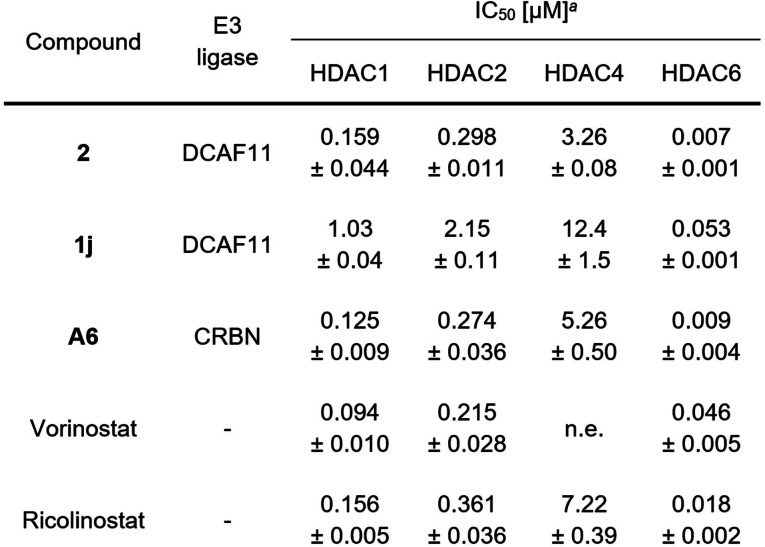
HDAC isoform inhibition by DCAF11‐recruiting PROTACs.

^*a*^Mean±standard deviation is shown of at least two independent experiments,each performed in duplicates. n.e.: no effect (≤30 % inhibition up to 10 μM).

**Figure 5:**
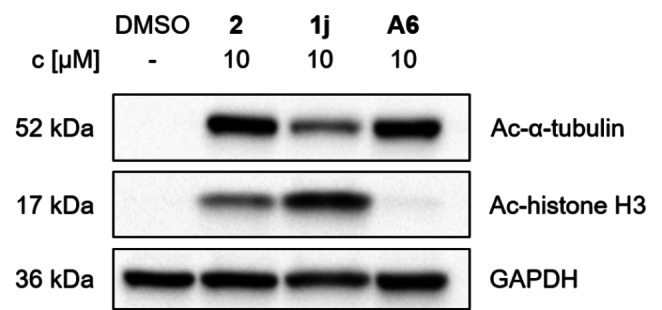
Cellular target engagement by **2**, **1j**, and **A6**. Immunoblot analysis of acetylated α‐tubulin and histone H3 in MM.1S cell lysates. Cells were treated with the indicated compounds (10 μM) or vehicle (DMSO) for 24 h. Representative images from a total of n=3 biological replicates.

Moreover, the capability to reduce the enzymatic activity of HDACs was confirmed by a cellular target engagement assay. MM.1S cells were treated with the PROTACs and the level of acetylated HDAC substrate proteins was subsequently determined. Class I HDACs deacetylate histone H3, and thus hyperacetylation of histone H3 indicates class I HDAC inhibition. Conversely, hyperacetylation of α‐tubulin serves a marker of HDAC6 inhibition. Both hit compounds are capable of inhibiting class I and HDAC6 in a cellular environment, confirming effective target engagement in MM.1S cells (Figure 5). With regards to the HDAC6 inhibition, the trend of the enzyme inhibition data was thus confirmed. However, histone H3 hyperacetylation was more pronounced by **1j**, compared to **2** and **A6**.

**Figure 6:**
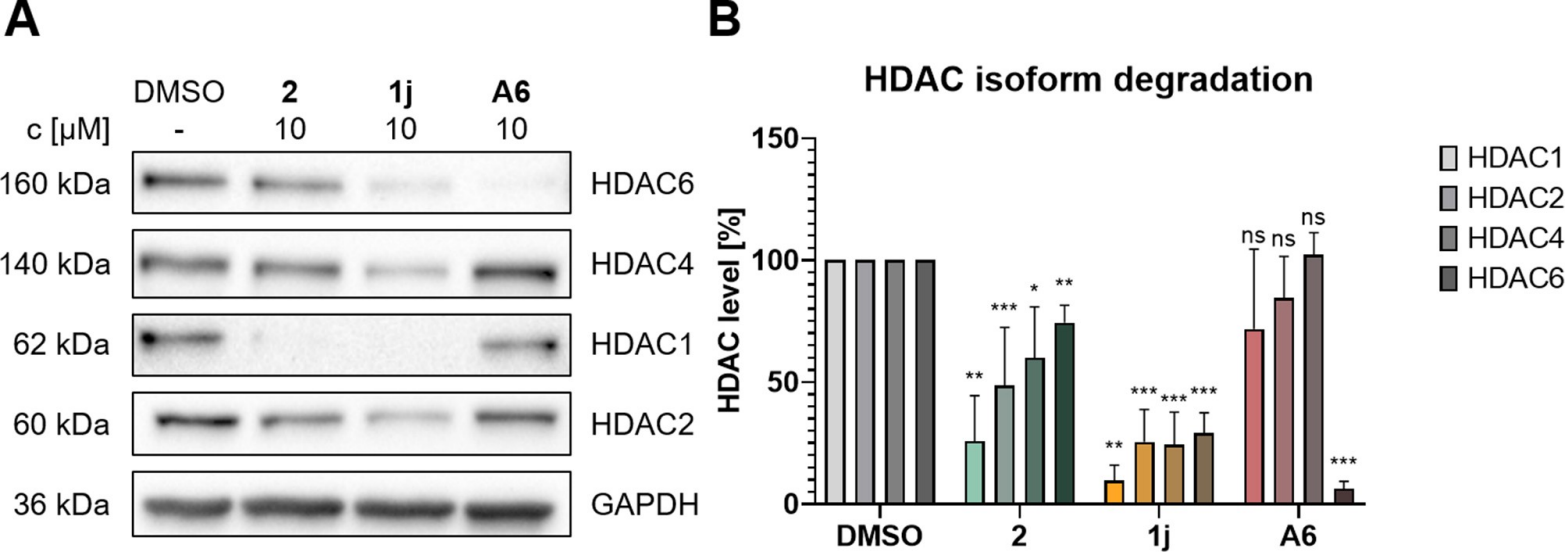
Examination of HDAC isoform degradation. MM.1S cells were treated with the indicated compounds (10 μM) or vehicle (DMSO) for 24 h. HDAC1, 2, 4, and 6 levels were analyzed by immunoblotting. (A) Representative images from a total of n=3 biological replicates. (B) Quantification of HDAC1, 2, 4, and 6 presented as mean ± standard deviation of n=3 biological replicates; significance compared to vehicle: ns=p≥0.05; *=p≤0.05; **=p≤0.01; ***=p≤0.001.

In the next step, we investigated whether this target engagement translates into degradation of multiple HDAC isoforms. HDAC1, 2, 4, and 6 levels were determined by immunoblot analysis of MM.1S cell lysates and the CRBN‐recruiting PROTAC **A6** was used as control. The immunoblot analysis confirmed the automated Simple Western™ immunoassay results, as **1j** showed most pronounced degradation of all tested HDAC isoforms (Figure 6). In detail, the strongest maximal degradation (*D*_max_) was observed for HDAC1 (90%) by **1j**, while the degradation of HDAC2, 4, and 6 ranged from 71–76%. *D*_max_ of compound **2**, although slightly less efficient, was also the strongest for HDAC1 (74%) followed by HDAC2 (51%), while HDAC4 (40%) and HDAC6 (26%) were also degraded, but to a lesser extent (see Table S1, Supporting Information). In contrast, **A6** selectively degraded HDAC6 without significant effects on the other HDAC isoforms, as previously reported.^[46]^ By utilizing DCAF11 instead of CRBN for HDAC degradation, **1j** and **2** are able to broaden the scope of degraded HDAC isoforms, degrading not only HDAC6 but also HDAC1, 2, 4, and 6. In addition, the degradation pattern of **1j** was further investigated by its time dependency. Treatment of MM.1S cells revealed significant degradation of HDAC1 and 6 after 6 h, which increased after 14 h and 24 h (Figure S4, Supporting Information).

### Cell Cycle Arrest and Apoptosis Induction of 2 and 1j

**Figure 7:**
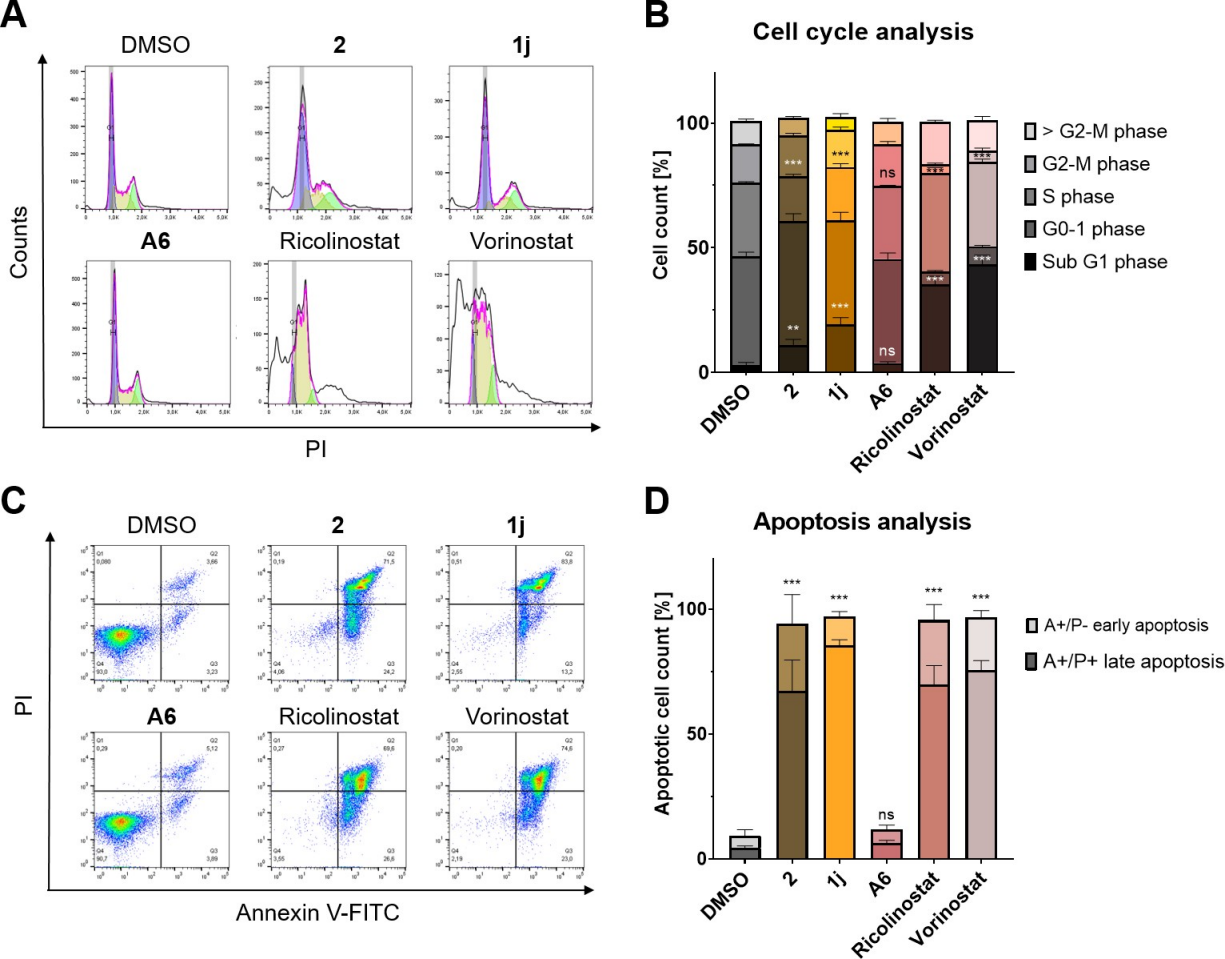
Cell cycle and apoptosis induction analysis by flow cytometry. MM.1S cells were treated with the respective compound (10 μM) or vehicle (DMSO) for 48 h. (A) Representative data of cell cycle analysis of MM.1S cells after PI staining. (B) Quantification of cell cycle analysis, presented as mean ± standard deviation of n=3 biological replicates, each performed in duplicates. (C) Representative data of apoptosis induction analysis of MM.1S cells after annexin V‐FITC/PI staining. (D) Quantification of apoptosis induction analysis, presented as mean ± standard deviation of n=3 biological replicates, each performed in triplicates. Significance of apoptosis analysis is regarding the combined quantities of early and late apoptosis. In the case of (B) and (D), significance of quantification is compared to vehicle: ns=p≥0.05; **=p≤0.01 and ***=p≤0.001.

To gain a more comprehensive understanding of the cellular effects of **2** and **1j**, cell cycle and apoptosis induction was analyzed. After treating MM.1S cells with the indicated compounds or vehicle for 48 h, the cells were permeabilized, fixed, and stained with propidium iodide (PI) to quantify the DNA content by flow cytometry (Figure 7A, B). The treatment of both PROTACs (**2**, **1j**) resulted in an increase of the sub G1 phase, while the S phase was decreased. This indicates cell cycle arrest and apoptosis induction. In contrast, the CRBN‐recruiting PROTAC **A6** indicated no significant changes in cell cycle. However, the included HDACi ricolinostat and vorinostat both showed stronger effects on the different phases of the cell cycle. The apoptosis induction was additionally assessed by staining with FITC labeled annexin V and PI. Flow cytometry analysis with these two dyes enables the distinctions between early and late apoptotic cells besides unaffected cells (Figure 7C, D). This analysis revealed less differences between the DCAF11‐recruiting PROTACs and the HDACi. All compounds significantly induced apoptosis, to a comparable extent. The only exception is **A6**, which stayed on the level of vehicle control. This proves that the antiproliferative activity of **2** and **1j** in MM.1S cells is based on the arrest of cell cycle and of induction of apoptosis.

### Synthesis and Evaluation of the Non‐Degrading Control 1j‐nc

**Scheme 2:**
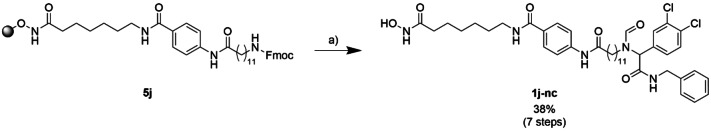
Synthesis of the non‐degrading control (**1j‐nc**) based on **1**. *Reagents and conditions*: a) (i) 20% piperidine, DMF, rt, 2×5 min, (ii) 3,4‐dichlorobenzaldehyde, formic acid, benzyl isocyanide, DMF/MeOH (1/1 *v*/*v*), rt, 72 h, (iii) 5% TFA, 5% triisopropylsilane, CH_2_Cl_2_, rt, 1 h.

In the next step, we wanted to design and synthesize a non‐degrading control for the most effective PROTAC **1j**. It is essential that the non‐degrading control is as comparable to the PROTAC as possible, without binding to the E3 ligase. By the absence of degradation, the non‐degrading control proves TPD, which requires the E3 ligase for the reduction of protein levels. In addition, it enables to distinguish between inhibitor and degrader effects. In case of **1j** the non‐degrading control was designed by substituting the electrophilic warhead of the DCAF11 ligand. The replacement of 2‐chloroacetic acid by propionic acid led to no successful U‐4CR, because of reduced nucleophilicity of the propionic acid. We successfully obtained the U‐4CR product bound on resin by switching to formic acid. After cleavage and purification, as described before, **1j‐nc** was prepared in 38% yield over seven steps (Scheme 2).

**Figure 8:**
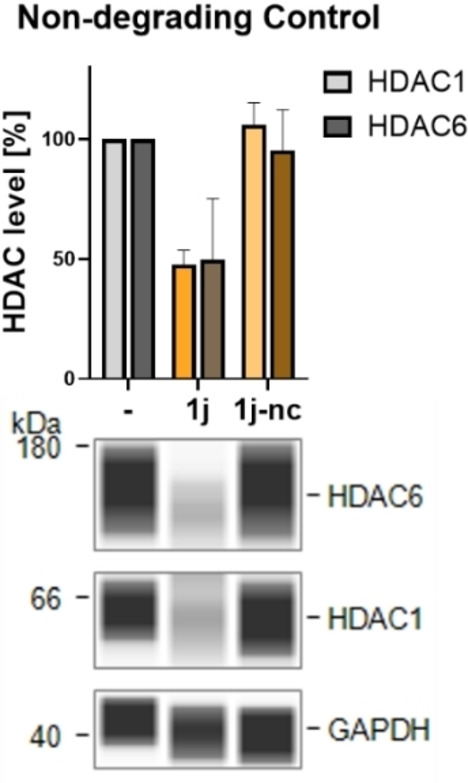
Evaluation of the effects of the non‐degrading control **1j‐nc** on HDAC protein levels. Simple Western™ immunoassay analysis of MM.1S cell lysates. MM.1S cells were incubated with **1j** (10 μM), **1j‐nc** (10 μM), or vehicle (DMSO) for 14 h. Representative image from a total of n=2 biological replicates.

The non‐degrading control was then used to treat MM.1S cells in direct comparison to the related PROTAC **1j**. In comparison to **1j**, the non‐degrading control **1j‐nc** was not able to affect the protein levels of HDAC1 and 6 (Figure 8), proving that covalent binding to DCAF11 is crucial for degradation.

### Cross Cell Line Activity of 2 and 1j

Encouraged by the positive results in the multiple myeloma cell line MM.1S, we wanted to expand the application of the DCAF11 PROTACs to other cancer models. The triple negative breast cancer cell line MDA‐MB‐231 and the glioblastoma cell line U‐87MG were selected as they depict aggressive and hard to treat solid tumors, in which HDACi showed first promising effects.[^53^, ^54^] The phenotypic cell viability screening demonstrated that both PROTACs exhibited cytotoxicity against all tested cell lines (Table 2). However, the effect was diminished in the solid cancer cell lines, particularly for U‐87MG, in comparison to the multiple myeloma cell line MM.1S. As anticipated, the non‐degrading control **1j‐nc** demonstrated no activity in MDA‐MB‐231, indicating that degradation is the underlying cause of cytotoxicity. However, **1j‐nc** exhibited some impact on MM.1S and U‐87MG, but consistently weaker compared to the PROTACs.

**Table 2:**
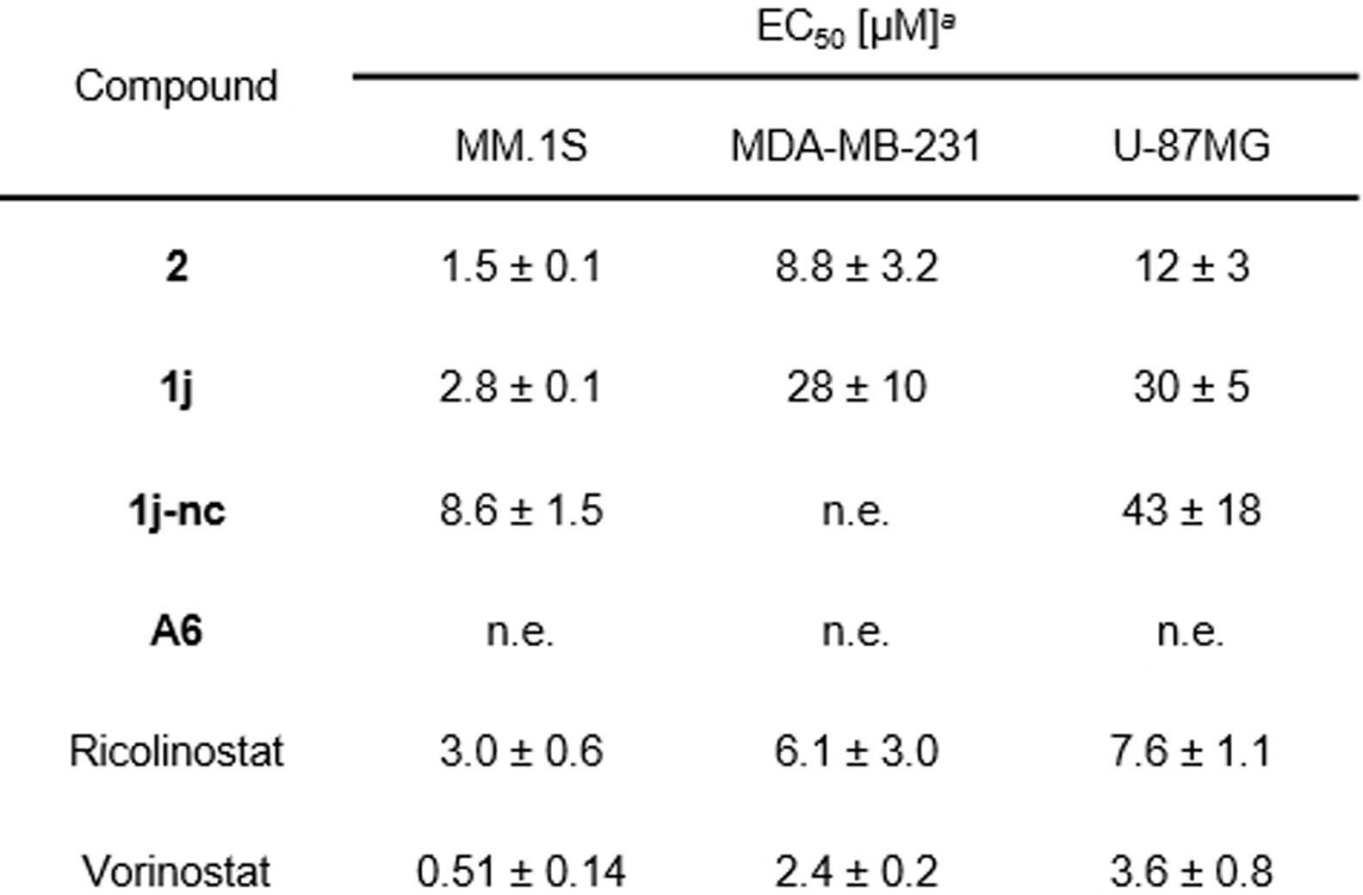
Antiproliferative activity of DCAF11 PROTACs against different tumor entities.

^*a*^Mean±standard deviation of at least three independent experiments. n.e.: no effect (≤25 % effect up to 50 μM). Antiproliferative activity of respective compounds in different cancer cell lines after 72 h.

As **2** and **1j** led to reduced cytotoxicity in U‐87MG cells, we analyzed HDAC1 and 6 degradation in this cell line. We chose the same treatment conditions as for MM.1S and the automated Simple Western™ immunoassay analysis confirmed significant degradation for HDAC1 by **2** and significant HDAC1 and 6 degradation by **1j** (Figure S5, Supporting Information). Nevertheless, the degradation was weaker in U‐87MG cells compared to MM.1S which is in good agreement with data from the viability assays.

### Clonogenic Growth Inhibition by 2 and 1j

**Figure 9:**
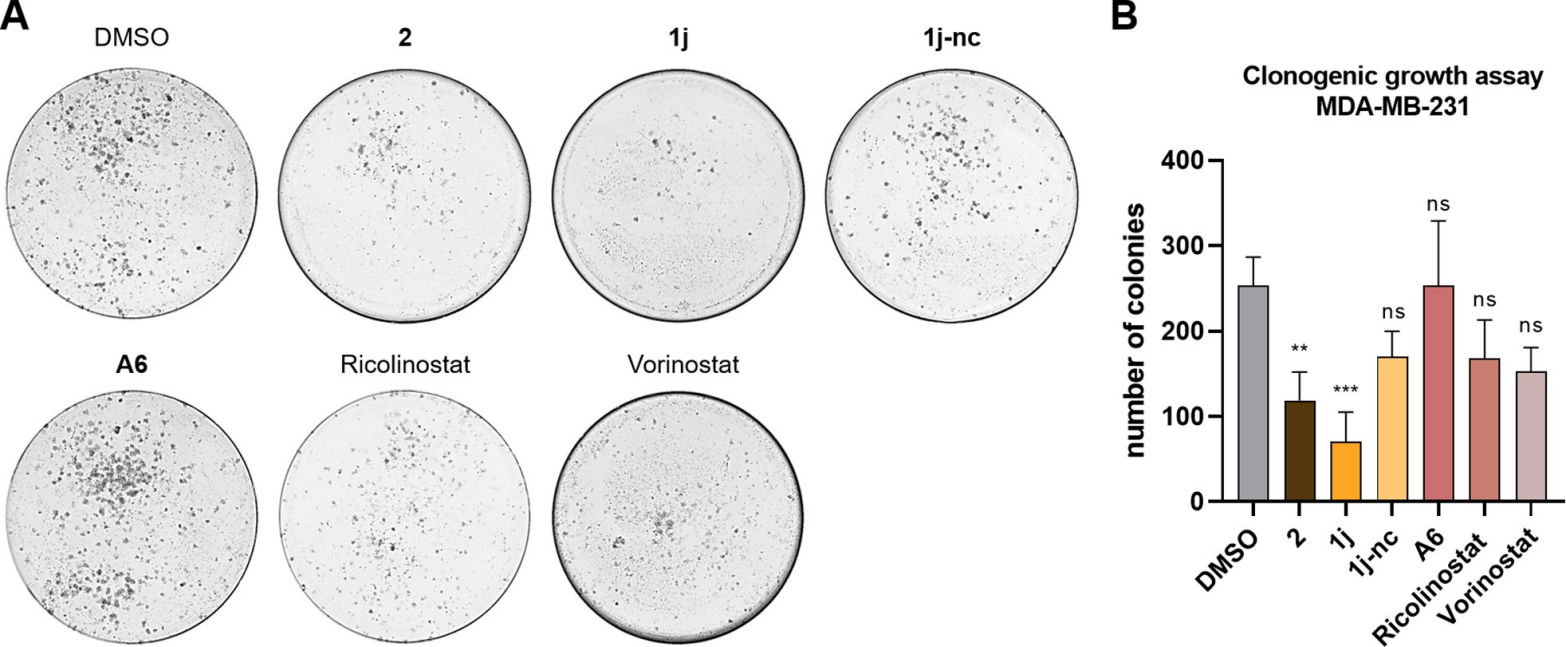
Clonogenic growth assay of DCAF11‐recruiting PROTACs. MDA‐MB‐231 cells were treated with 10 μM of the respective compound or vehicle (DMSO) for 48 hours. (A) Representative images of crystal violet stained colonies after nine days of growth. (B) Quantification of counted colonies, presented as mean ± standard deviation of three biological replicates, each performed in triplicates. Significance of quantification compared to vehicle: ns=p≥0.05; **=p≤0.01 and ***=p≤0.001.

The short‐term effects on cancer cell lines were supported by a clonogenic growth assay. Using this assay, we could study the long‐term anticancer effects of DCAF11‐recruiting HDAC PROTACs. The solid cancer cell line MDA‐MB‐231 was chosen for this investigation, because of the elevated effect on cell viability and the better growth pattern, compared to U‐87MG. The triple negative breast cancer cells were incubated for 48 h with the respective compound, before they were isolated and grown for nine days. The resulting colonies were quantified and are depicted in Figure 9. Both **2** and **1j** resulted in a significant reduction of colonies. However, **1j** demonstrated a more pronounced reduction in clonogenic growth, which aligns with the enhanced HDAC degradation. The two HDACi, included as a control, demonstrated a trend to a reduction in colony count, albeit less pronounced and not significant. Notably, **2** and **1j** outperformed ricolinostat and vorinostat in the long‐term clonogenic growth assays. In contrast, the thalidomide‐based HDAC PROTAC **A6** did not result in a significant reduction in clonogenic growth.

## Conclusions

In summary, we present the first‐in‐class DCAF11‐recruiting HDAC PROTACs. These novel compounds are derived from our previously reported HDAC PROTAC **A6** but feature a DCAF11 ligand in place of the CRBN ligand. The synthesis was performed entirely on solid‐phase, which accelerated the synthesis and gave compounds **1a‐j** and **2** in yields of up to 57% over seven steps. The subsequent evaluation of the PROTACs for HDAC degradation uncovered **2** and **1j** (hereafter dubbed **FF2039**) as the most effective HDAC1 degraders with additional HDAC6 degradation capabilities. Moreover, we demonstrated that HDAC degradation translates into antiproliferative activity against MM.1S cells, with cytotoxicity appearing to correlate with the degree of HDAC1 degradation. An *in vitro* enzyme inhibition assay revealed that **2** showed comparable inhibition to **A6** against HDAC1, 2, 4, and 6, whereas **FF2039** displayed reduced inhibition of all tested isoforms. In contrast, the cellular target engagement analysis showed a distinct inhibition of HDAC6 by both PROTACs and a stronger inhibition of class I HDACs by **2** and **FF2039** compared to **A6**. Both **2** and **FF2039** were able to significantly degrade HDACs of class I, IIa, and IIb, with **FF2039** emerging as the more efficient degrader. In detail, **FF2039** could achieve a pronounced degradation of all four tested isoforms (*D*_max_: 71–90%), demonstrating robust pan‐HDAC degradation activity. This demonstrates not only the successful incorporation of a DCAF11 recruiter for targeted HDAC degradation but also underscores the critical role of the chosen E3 ligase in governing degradation selectivity. Similar to the shift in degradation selectivity observed when PROTAC **IV** was transitioned to **V** by replacing IAP with VHL, we showed that substituting the CRBN ligand with a DCAF11 recruiter shifted selectivity from HDAC6‐specific to pan‐HDAC degradation.^[29]^

This change in the selectivity profile creates new opportunities to investigate and compare HDAC isoform‐specific or general effects. Importantly, PROTACs affect not only the enzymatic but also the non‐enzymatic functions of their targets. While HDAC6 selective degraders allow for a more detailed assessment of HDAC6 function in pathology and physiology, a pan‐HDAC degrader may provide a more general picture of HDACs functions across different isoforms and may also reveal isoform synergies in terms of phenotypic effects such as anticancer activity. In addition, both approaches may have potential clinical applications. While pan‐HDAC degraders could be developed to treat multiple cancers,^[51]^ selective HDAC6 PROTACs, like selective HDAC6 inhibitors,^[55]^ could be used in neurodegenerative diseases, inflammatory diseases, and some cancers. In the latter case, it would be important to replace the non‐selective HDAC inhibitor warhead in **A6** with a selective HDAC6 inhibitor scaffold to avoid class I effects resulting from HDAC inhibition.

To verify that degradation is mediated via DCAF11 recruitment, we synthesized the non‐degrading control **1j‐nc** based on **FF2039**. No effects on HDAC1 and HDAC6 levels were observed with **1j‐nc**, confirming the necessity of covalent binding to the E3 ligase for effective degradation. In addition, we could show that **2** and **FF2039** possess antiproliferative activity in the triple negative breast cancer cell line MDA‐MB‐231 and the glioblastoma cell line U‐87MG. The non‐degrading control showed no or less effects in all cell lines. Furthermore, for a deeper understanding of the anticancer properties, cell cycle and apoptosis induction were examined in MM.1S cells. Compound **2** and **FF2039** induced greater cell cycle arrest and apoptosis compared to the CBRN‐recruiting **A6**. An investigation of the long‐term effects on the solid tumor cell line MDA‐MB‐231 revealed that the DCAF11‐recruiting degraders significantly reduced clonogenic growth, outperforming the HDACi vorinostat and ricolinostat, whereas **A6** showed no reduction.

Taken together, we developed the first DCAF11‐based HDAC degraders and our most advanced degrader, **FF2039**, represents the first tool to achieve pan‐HDAC degradation. This elucidates the effects of the selected E3 ligase for TPD.

## Funding Sources

Funding by the Deutsche Forschungsgemeinschaft (DFG, German Research Foundation)–GRK2873 (494832089) is gratefully acknowledged. The new NMR console for the 500 MHz NMR spectrometer used in this research was funded by the Deutsche Forschungsgemeinschaft (DFG, German Research Foundation) under project number 507275896.

## Conflict of Interests

The authors declare no conflict of interest.

1

## Supporting information

As a service to our authors and readers, this journal provides supporting information supplied by the authors. Such materials are peer reviewed and may be re‐organized for online delivery, but are not copy‐edited or typeset. Technical support issues arising from supporting information (other than missing files) should be addressed to the authors.

Supporting Information

## Data Availability

The data that support the findings of this study are available from the corresponding author upon reasonable request.
