# Supplementary material for: Replacing a Cereblon Ligand by a DDB1 and CUL4 Associated Factor 11 (DCAF11) Recruiter Converts a Selective Histone Deacetylase 6 PROTAC into a Pan‐Degrader
Source: ChemMedChem. 2025 Mar 24;20(10):e202500035. doi: 10.1002/cmdc.202500035 (PMC12091851; doi:10.1002/cmdc.202500035)
Supplement: Supplementary file 1 — Supporting Information [file CMDC-20-e202500035-s001.pdf]

ChemMedChem

Supporting Information

Replacing a Cereblon Ligand by a DDB1 and CUL4 Associated Factor 11 (DCAF11) Recruiter Converts a Selective Histone Deacetylase 6 PROTAC into a Pan-Degrader

Felix Feller, Heiko Weber, Martina Miranda, Irina Honin, Maria Hanl, and Finn K. Hansen*

Supplementary Information

Replacing a Cereblon Ligand by a DDB1 and CUL4 Associated Factor 11 (DCAF11) Recruiter Converts a Selective Histone Deacetylase 6 PROTAC into a Pan-Degrader

Felix Feller,[a] Heiko Weber,[a] Martina Miranda,[a] Irina Honin,[a] Maria Hanl,[a] Finn K. Hansen[a]*
[a]Department of Pharmaceutical and Cell Biological Chemistry, Pharmaceutical Institute, University of Bonn, An der Immenburg 4, 53121 Bonn, Germany.

Table of contents

1. Supplementary Tables and Figures S3
Figure S1. ..... S3
Figure S2. ..... S3
Figure S3. ..... S4
Figure S4. ..... S4
Figure S5. ..... S5
Scheme S1. ..... S6
Table S1 ..... S6
2. Biological Experiments S7
2.1. Cell Culture S7
2.2. Simple Western™ Immunoassay S7
2.3. CellTiter-Glo® Cell Viability Assay S8
2.4. Cellular HDAC Inhibition Assay S8
2.5. Aqueous Stability Assay S9
2.6. Immunoblot S9
2.7. HDAC Enzyme Inhibition Assay S9
2.8. MTT Cell Viability Assay S10
2.9. Cell Cycle Analysis S10
2.10. Annexin V/PI Assay S11
2.11. Clonogenic growth assay S11
3. Chemical Experiments S12
3.1. General Information S12
3.2. General Procedures S13
3.3. Preparation of Compounds S14

3.4. NMR Data of compounds in biological testing S26
3.5. HPLC chromatograms of target compounds S39
4. References S46

1. Supplementary Tables and Figures

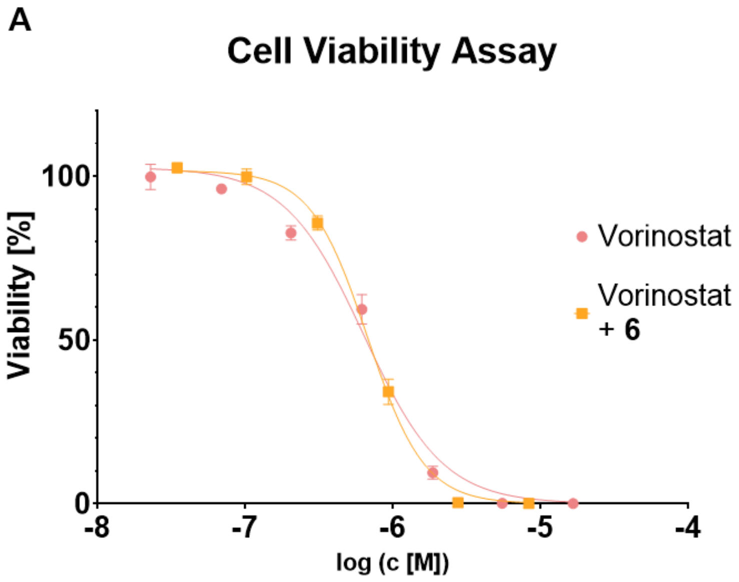

B

Compound	EC_{50} [µM] MM.1S
Vorinostat	0.59 ± 0.09
Vorinostat + **6**	0.73 ± 0.06

Figure S1. Antiproliferative activity of DCAF11-ligand **6** in combination (1:1) with vorinostat compared to the single treatment with vorinostat in MM.1S cells after 72 h. (A) Representative concentration response curves. (B) Mean ± standard deviation of EC_{50} determination of two independent experiments, each performed in duplicates.

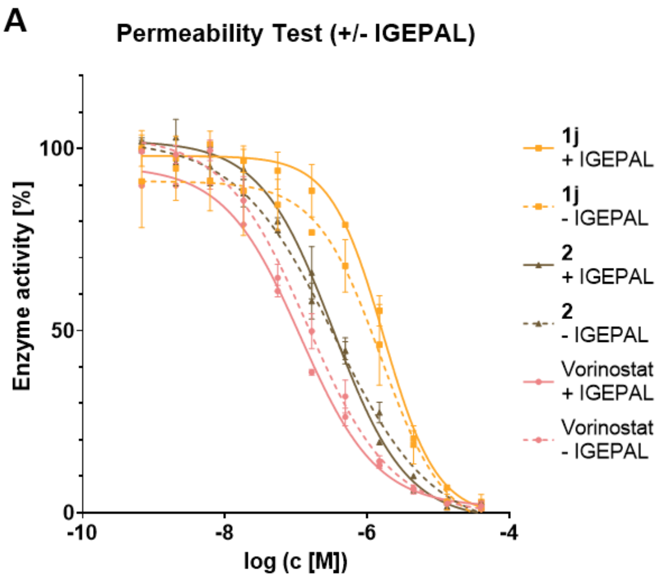

B

	IC_{50} + IGEPAL [µM]	IC_{50} - IGEPAL [µM]
**1j**	1.7 ± 0.1	1.7 ± 0.2
**2**	0.33 ± 0.01	0.41 ± 0.09
Vorinostat	0.13 ± 0.01	0.15 ± 0.01

Figure S2. Cell permeability test by a cellular HDAC inhibition assay performed in the presence and absence of the cell permeabilizing agent IGEPAL. MM.1S cells were treated with increasing concentrations of the respective compounds. (A) Representative concentration response curves. (B) Mean ± standard deviation of IC_{50} determination of two independent experiments, each performed in duplicates.

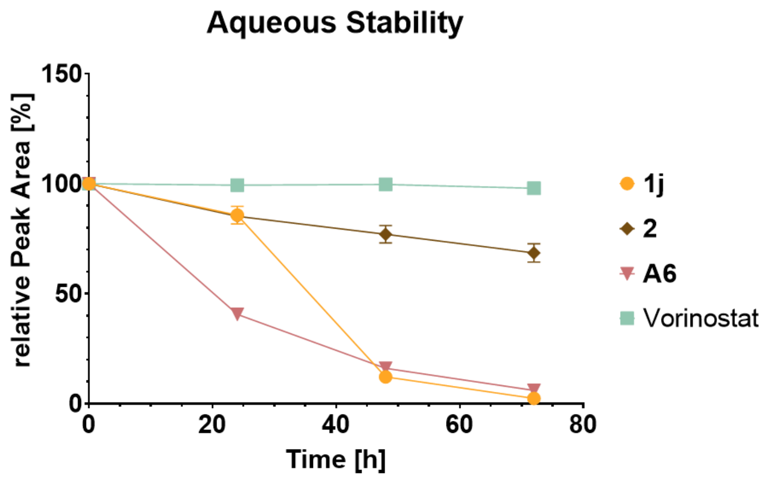

Figure S3. Aqueous stability of the DCAF11-recruting PROTACs (**1j** and **2**) compared to vorinostat and **A6**. Compounds were diluted in DPBS buffer (pH = 7.4; final DMSO concentration: 10%) and the remaining compound content was analyzed by HPLC. The peak area at 0 h was used for normalization. Mean ± standard deviation of n = 2 replicates.

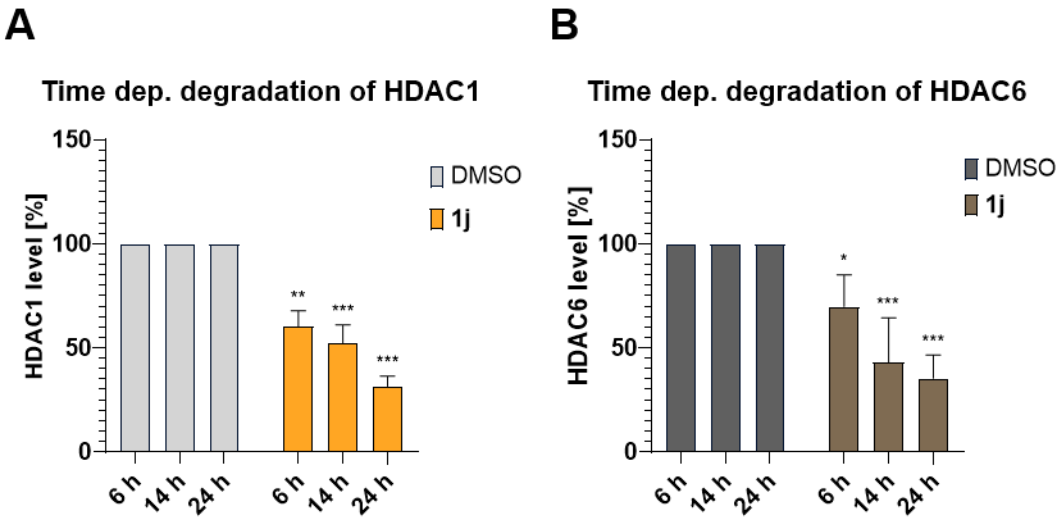

Figure S4. Analysis of time dependent degradation by **1j** of HDAC1 (A) and HDAC6 (B) by Simple Western™ immunoassay analysis of MM.1S cell lysates. MM.1S cells were treated with **1j** (10 µM) or vehicle (DMSO) for the indicated time period. Quantification of protein levels presented as mean ± standard deviation of n = 3 biological replicates. Significance compared to vehicle: * = p≤0.05, ** = p≤0.01, *** = p≤0.001.

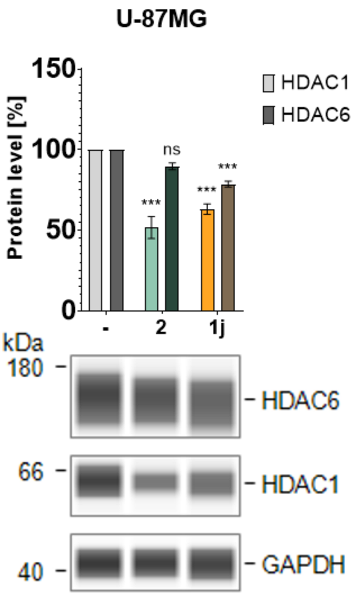

Figure S5. Analysis of HDAC1 and 6 degradation in U-87MG cells. Simple western immunoassay analysis of U-87MG cell lysates. The cells were treated with the indicated compound (10 µM) or vehicle (DMSO) for 24 h. Top: Quantification of HDAC levels, presented as mean ± standard deviation of n = 2 biological replicates; Significance compared to vehicle: ns = p≥0.05 and *** = p≤0.001. Bottom: Representative images of n = 2 biological replicates.

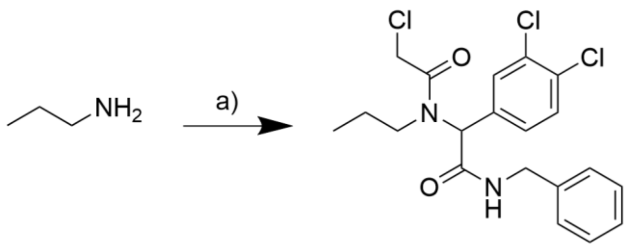

6
53%

Scheme S1. Synthesis of the DCAF11 ligand **6**. *Reaction conditions*: 3,4-dichlorobenzaldehyde, 2-chloroacetic acid, benzyl isocyanide, MeOH, rt, 72 h.

Table S1. Maximal degradation (D_{max}) of PROTAC **1j** and **2** of the respective HDAC isoforms.

	D_{max}[a]	
	**1j**	**2**
HDAC1	90 ± 6	74 ± 19
HDAC2	75 ± 13	51 ± 24
HDAC4	76 ± 13	40 ± 20
HDAC6	71 ± 8	26 ± 7

[a]MM.1S cells were treated with the indicated compounds (10 µM) or vehicle (DMSO) for 24 h. HDAC1, 2, 4, and 6 levels were analyzed by immunoblotting. The quantification of the D_{max} values is presented as mean ± standard deviation of n = 3 biological replicates.

2. Biological Experiments

No unexpected or unusually high safety hazards were encountered.

2.1. Cell Culture

The human multiple myeloma cell line MM.1S (CRL-2974) was obtained from American Type Culture Collection (ATCC, Manassas, VA, USA). The semi-adherent cells were cultivated in RPMI 1640 medium (Catalog#21875-034, Gibco, ThermoFisher Scientific Inc., Waltham, MA, USA) supplemented with 10% FBS (PAN Biotech GmbH; Aidenbach, Germany), 100 IU/mL penicillin, 0.1 mg/mL streptomycin (PAN Biotech GmbH) and 1 mM sodium pyruvate (ThermoFisher Scientific Inc.). The human breast cancer cell line MDA-MB-231 (HTB-26) and the human malignant glioma cell line U-87MG were cultivated in DMEM medium (Catalog#41966-029, Gibco, ThermoFisher Scientific Inc.) supplemented with 10% FBS (PAN Biotech GmbH), 100 IU/mL penicillin and 0.1 mg/mL streptomycin (PAN Biotech GmbH). All cells were cultured at 37 °C in a 5% CO_2 atmosphere. Incubation times, as part of assay protocols, were performed under these conditions as well. The semi-adherent MM.1S were detached mechanically by using a cell scraper and the adhered MDA-MB-132 were trypsinased by trypsin/EDTA (0.05%/0.02% in DPBS, PAN Biotech GmbH) whereas the U-87MG were detached by EDTA (0.46 g/L in DPBS, Catalog# P04-36500).

2.2. Simple Western™ Immunoassay

For the cell lysates, MM.1S (0.5×10^6 cells/mL) or U-87MG (0.18×10^6 cells/mL) cells were seeded and incubated with the indicated concentration of compound or vehicle (DMSO) for the given time. The adherent cells were cultured for 24 h under cell culture conditions for attachment before treatment. Cell lysis was performed with Cell Extraction Buffer (10 mM Tris, pH 7.4, 100 mM NaCl, 1 mM EDTA, 1 mM EGTA, 1 mM NaF, 20 mM $Na_4P_2O_7$, 2 mM Na_3VO_4, 1% Triton™ ×-100, 10% glycerol, 0.1% SDS, 0.5% deoxycholate; Catalog# FNN0011, Thermo Fisher Scientific Inc., Waltham, MA, USA) with addition of Halt Protease Inhibitor Cocktail (100×) (Catalog# 78429, Life Technologies GmbH, Carlsbad, CA, USA) and phenylmethanesulfonyl fluoride (Catalog# 10837091001, Sigma-Aldrich, St. Louis, MO, USA) according to manufacturer´s instructions. Protein content was determined by Pierce™ BCA Protein Assay Kit (Catalog# 23225, Thermo Fisher Scientific Inc.) according to manufacturer´s guidelines. Cell lysates were diluted to a final concentration of 1 mg/mL and denatured according to manufacturer's guidelines. Anti-HDAC1 (Catalog# 5356S, Cell Signaling Technology, Denver, MA, USA), anti-HDAC6 (Catalog#7558S, Cell Signaling Technology) and anti-GAPDH (Catalog# 2118S, Cell Signaling Technology) antibodies were used in a dilution from 1:50-1:1250. The 12-230 kDa Fluorescence Separation 8x25 Capillary Cartridges (Catalog# SM-FL004-1, Protein Simple, Bio-Techne, Minneapolis, MN, USA) were used for separation and the Anti-Mouse Detection Module (Catalog# DM-002, Protein Simple) was used for detection with addition of 20x Anti-Rabbit HRP

Conjugate (Catalog# 043-426, Protein Simple) according to manufacturer´s instructions. The assay was performed with the Jess Simple Western System (catalog# 004-650, Protein Simple), according to manufacturer´s settings. Evaluation and quantification was performed with the compass software (6.2.0, Protein Simple). GraphPad Prism (GraphPad Software, San Diego, CA, USA) was used for normalization, statistical analysis and bar graph creation. Significance testing was performed with a one-way analysis of variance (ANOVA). Dunnett's post-hoc test was employed to obtain significance levels of each mean compared to a reference mean value.

2.3. CellTiter-Glo® Cell Viability Assay

For the cell viability assay the automated pipetting robot system ASSIST PLUS (Model# 4505, Integra Biosciences, Bibertal, Germany) was used. MM.1S (2.5×10^3 cells/well) were seeded in white 384-well plates (Greiner Bio-One, Kremsmuenster, Austria, #781080). The cells were incubated with the respective compounds in increasing concentrations. For this purpose, the dilution series were prepared in 200-fold concentration in DMSO and further diluted to 10-fold concentration in medium. The final DMSO concentration was 0.5%. The toxicity of compounds was determined after 72 h using the CellTiter-Glo 2.0 Cell Viability Assay (Promega, Madison, WI, USA, #G9242) according to the manufacturer´s protocol. Subsequently, the luminescence was measured using a Tecan Spark (Tecan Group AG, Maennedorf, Swiss) and the half maximal effective concentration (EC_{50}) was determined by plotting dose response curves and nonlinear regression with GraphPad Prism (GraphPad Software, San Diego, CA, USA).

2.4. Cellular HDAC Inhibition Assay

The HDAC whole cell assay is based on the assay established by Ciossek et al.[1] and Bonfils et al.[2] with minor changes. MM.1S multiple myeloma cells were seeded in a concentration of 75×10^3 cells/well (total volume of 189 µL) in 96-well cell culture microplates (Catalog# 655086, Greiner Bio-One). The DMSO-stock solutions were used to perform a serial dilution in DMSO. Next, the cells were treated with 1 µL of the serial dilution (190 × concentrate) for 18 h. To investigate cell membrane penetration of the tested compounds, the substrate solution containing 3 mM Boc-Lys(ε-Ac)-AMC (Catalog# 233691-67-3, BLD pharma) was prepared with and without IGEPAL CA-630 (0.5%, Catalog# J61055, Alfa Aesar). 10 µL of the substrate solution was added and incubated for 3 h under cell culture conditions. The reaction was stopped with 100 µL of stop solution (50 mM Tris-HCl, 137 mM NaCl, 2.7 mM KCl, 1 mM $MgCl_2$, 1% IGEPAL CA-630, 10 µM Vorinostat, 2.0 mg/mL Trypsin) and incubated for 1.5 h under cell culture conditions. The fluorescence signal was measured by a FLUOstar OPTIMA microplate reader (BMG labtech, Ortenburg, Germany) at excitation of $\lambda = 355$ nm and emission of $\lambda = 460$ nm. All Compounds were tested in two independent experiments in duplicates and IC_{50} was determined by nonlinear regression with GraphPad Prim.

2.5. Aqueous Stability Assay

For stability testing in aqueous solution, a 10-fold concentrated DMSO solution of the respective compounds was diluted in DPBS to generate a 10 µM concentration (final DMSO content of 10%). Samples of the aqueous solutions were taken after 0, 24, 48, and 72 h at room temperature and the amounts of the parental compound and potential fragments were monitored by HPLC gradient A (see 3.1 General Information). The peak area of the 0 h sample was used for normalization. All compounds were tested in duplicates and GraphPad Prim was used for evaluation and data visualization.

2.6. Immunoblot

The cell lysis was performed as descripted in **3.2. Simple Western Immunoassay**. Samples were denatured by Laemmli 2× Concentrate (Catalog# S3401-10VL, Sigma-Aldrich) for 5 min at 95 °C, and Precision Plus Protein Unstained Standard was used as molecular weight marker (Catalog# 1610363, Bio-Rad, Hercules, CA, USA). SDS-PAGE was performed with 10 or 12% Mini-PROTEAN TGX Stain-Free Gel (Catalog# 458035, Bio-Rad) at 200 V for 50 min (Catalog# 458035, Bio-Rad). Afterwards, proteins were transferred with the Trans-Blot Turbo Transfer System (Catalog# 1704150, Bio-Rad) to Immobilon-FL PVDF membranes (Catalog# IPFL00005, Millipore Merck, Burlington, MA, USA) at 1.0 A for 30 min and treated with 5% milk-powder solution for 1 h at room temperature under slight agitation. Subsequently, the membranes were treated with anti-HDAC1 (Catalog# 5356S, Cell Signaling Technology), anti-HDAC2 (Catalog# sc-9959, Santa Cruz Biotechnology, Dallas, TX, USA), anti-HDAC4 (Catalog#7628S, Cell Signaling Technology), anti-HDAC6 (Catalog#7558S, Cell Signaling Technology), anti-acetyl-histone H3 (Catalog# 9677S, Cell Signaling Technology), anti-acetyl-α-tubulin (Catalog#5335, Cell Signaling Technology) or anti-GAPDH (Catalog# 2118S, Cell Signaling Technology) antibody solutions in 1:1000–1:8000 dilutions at 4 °C overnight. Treatment with HRP-conjugated secondary anti-mouse (Catalog# 1030-05, SouthernBiotech, Birminham, AL, USA) and anti-rabbit (Catalog# 4030-05, SouthernBiotech) antibody solution was performed for 1.5 h and membranes were developed with clarity western ECL substrate (Catalog# 1705061, Bio-Rad). The ChemiDoc XRS+ System (Catalog# 1708265, Bio-Rad) was used for detection and Image Lab Software 6.1 (Bio-Rad) for quantification. GraphPad Prism (GraphPad Software) was used for normalization, statistical analysis, and bar graph creation. Significance testing was performed with a one-way analysis of variance (ANOVA). Dunnett's post-hoc test was employed to obtain significance levels of each mean compared to a reference mean value.

2.7. HDAC Enzyme Inhibition Assay

In vitro inhibitory activities against HDAC1, 2, and 6 were measured using a previously published protocol.[3] *In vitro* inhibitory activities against HDAC4 were measured using a previously published protocol with slight modifications.[4] For test compounds and controls, serial dilutions of the respective DMSO stock solution in DMSO were prepared, and 1.0 µL/well of this serial dilution were transferred

into OptiPlate-96 black microplates (PerkinElmer, Waltham, MN, USA). A volume of 4 µL/well assay buffer (50 mM Tris−HCl, pH 8.0, 137 mM NaCl, 2.7 mM KCl, 1.0 mM MgCl2·6H2O, 0.1 mg/mL BSA) was added. In the case of HDAC1, 2, and 6, a volume of 35 µL/well of the fluorogenic substrate ZMAL (Z- Lys(Ac)-AMC,[5] 21.43 µM in assay buffer) and 10 µL/well enzyme solution were added. In the case of HDAC4, 35 µL/well of the fluorogenic substrate Boc-Lys(Tfa)-AMC (42.86 µM in assay buffer, Catalog# 4060676, Bachem, Budendorf, Swiss) were added, followed by 10 µL/well of enzyme solution. Human recombinant HDAC1 (Catalog# 50051, BPS Bioscience, San Diego, CA, USA), HDAC2 (Catalog# 50052, BPS Bioscience), HDAC4 (Catalog# 50004, BPS Bioscience), or HDAC6 (Catalog# 50006, BPS Bioscience) were used. The total assay volume of 50 µL (max. 2% DMSO) was incubated at 37 °C for 90 min. Subsequently, 50 µL/well of trypsin (0.4 mg/mL) in trypsin buffer (50 mM Tris−HCl, pH 8.0, 100 mM NaCl) was added, followed by additional 30 min of incubation at 37 °C. Fluorescence (excitation λ = 355 nm, emission λ = 460 nm) was measured using a FLUOstar OPTIMA microplate reader (BMG labtech, Ortenburg, Germany). The half maximal inhibitory concentration (IC_{50}) was determined by plotting dose response curves and nonlinear regression with GraphPad Prism (GraphPad Software).

2.8. MTT Cell Viability Assay

U-87MG cells (3×10^3 cells/well) or MDA-MB-231 cells (5×10^3 cells/well) were seeded in 96-well flat-bottomed plates. After 24 h the cells were incubated with the stock solutions of the respective compounds dissolved in DMSO (highest DMSO concentration, 0.5%) to reach the final concentrations as indicated. Controls received DPBS or DMSO. Following a 71 h incubation period 20 µl of a freshly prepared 5 mg/ml MTT (3-(4,5-dimethylthiazol-2-yl)-2,5-diphenyltetrazolium bromide, AppliChem GmbH, Darmstadt, Germany) solution were added into each well. The supernatant was discarded after 1 h incubation at 37 °C and the precipitated formazan was dissolved in 200 µl DMSO/well. Absorption was measured at 570 nm with background subtraction at 690 nm using a photometric microplate reader (Thermo Scientific Multiskan EX, Thermo Fisher Scientific). Data was further subtracted by the DMSO control and normalized on DPBS. The half-maximal effective concentration (EC_{50}) was determined by plotting dose response curves and nonlinear regression with GraphPad Prism (GraphPad Software).

2.9. Cell Cycle Analysis

MM.1S cells (3×10^3 cells/mL) were seeded in twelve well plates (Starlab GmbH, Hamburg, Germany) and incubated with the indicated concentration of compound or vehicle (DMSO) for 48 h under cell culture conditions. Subsequently, the cells were washed with DPBS (Catalog# P04-36500, PAN Biotech), resuspended in 1 mL EtOH/DPBS (7/3 *v*/*v*), and fixed for 30 min at 4 °C. The samples were rehydrated with DPBS and treated with 5 µg/mL RNAse (Catalog# EN0531, ThermoFisher Scientific Inc.) for 15 min at room temperature. The staining was performed with 3 µM propidium iodide (in DPBS) for 15 min at room temperature and analyzed by flow cytometry (Guava easyCyteTM, Luminex,

Austin, TX, USA). The data was analyzed by FlowJo v10.5.3 Software (BD Life Sciences, Franklin Lakes, NJ, USA), using the Watson Pragmatic algorithm.[6] GraphPad Prism (GraphPad Software) was used for normalization, statistical analysis, and bar graph creation. Significance testing was performed with a one-way analysis of variance (ANOVA). Dunnett's post-hoc test was employed to obtain significance levels of each mean compared to a reference mean value.

2.10. Annexin V/PI Assay

To determine apoptosis, MM1.S cells (0.5×10^6 cells/mL) were seeded in sterile 24-well plates (CytoOne, Hamburg, Germany) and incubated for 48 h at 37 °C and 5% CO_2 under humidified air with 10 µM of the respective compounds or vehicle control (DMSO). Final DMSO concentration was 0.1%. After 48 h, cells were washed with ice-cold DPBS, diluted in 100 µL staining buffer (HEPES 0.01 M, NaCl 0.14 M, $CaCl_2 \times 2\ H_2O$ 2.5 mM) and transferred to a 96 well plate. Staining was performed with 5 µL FITC Annexin V (Catalog#640945, Biolegend, San Diego, CA, USA) and 10 µL of propidium iodide (Catalog#421301, Biolegend) per well. Fluorescence was measured by flow cytometry (Guava® easyCyteTM, Luminex, Austin, Texas). GraphPad Prism (GraphPad Software) was used for normalization, statistical analysis, and bar graph creation. Significance testing was performed with a one-way analysis of variance (ANOVA). Dunnett's post-hoc test was employed to obtain significance levels of each mean compared to a reference mean value.

2.11. Clonogenic growth assay

5×10^5 MDA-MB-231 cells were seeded into T25 cell culture flasks and stimulated with 10 µM of respective compounds or vehicle (0.7% DMSO) for 48 hours. Subsequently, cells were harvested and plated in 6-well plates at a density of 750 cells per well in triplicates. The cell culture medium was changed after five days. Nine days after plating, the colonies were carefully washed with 500 µL DPBS and stained with 500 µL crystal violet solution (0.5% in methanol). After 30 minutes of incubation at room temperature, the wells were gently rinsed with deionized water. After counting the formed colonies, GraphPad Prism (GraphPad Software) was used for bar graph creation. Significance testing was performed with a one-way analysis of variance (ANOVA). Dunnett's post-hoc test was employed to obtain significance levels of each mean compared to a reference mean value.

3. Chemical Experiments

No unexpected or unusually high safety hazards were encountered.

3.1. General Information

Chemicals were purchased from ABCR, Acros Organics, BLDpharm, Carl Roth, Fisher Scientific, Fluorochem, Sigma Aldrich, Tokyo Chemical Industry, and VWR Chemicals. Fmoc protection was performed according to our previously published protocol.[7] Technical grade solvents were distilled prior to use. For all HPLC purposes, acetonitrile in HPLC-grade quality (HiPerSolv CHROMANORM, VWR) was used. Water was purified with a PURELAB flex® (ELGA VEOLIA). Acros Organics silica gel 60 (70–230 mesh) was taken for preparative column chromatography. Uncorrected melting points were measured on a Gallenkamp Melting Point Device (MPD350.BM3.5). A Thermo Fisher Scientific UltiMateTM 3000 UHPLC system with a Nucleodur 100-5 C18 (250 × 4.6 mm, Macherey Nagel) with a flow rate of 1 mL/min and a temperature of 25 °C or a 100-5 C18 (100 × 3 mm, Macherey Nagel) with a flow rate of 0.5 mL/min and a temperature of 25 °C with an appropriate gradient were used. For preparative purposes a AZURA Prep. 500/1000 gradient system with a Nucleodur 110-5 C18 HTec (150 × 32 mm, Macherey Nagel) column with 20 mL/min was used. Detection was implemented by UV absorption measurement at a wavelength of λ = 220 nm and λ = 250 nm. Bidest. H_2O (A) and MeCN (B) were used as eluents with an addition of 0.1% TFA for eluent A. The purity of all final compounds was 95% or higher. Purity was determined *via* HPLC with the Nucleodur 100-5 C18 (250 x 4.6 mm, Macherey Nagel) at 250 nm. The following gradients were applied: A (95% A and 5% B for 5 min, to 5% A and 95% B in 5 min, 5% A and 95% B for 12 min), B (95% A and 5% B for 5 min, to 5% A and 95% B in 10 min, 5% A and 95% B for 12 min). HR-ESI-MS spectra were recorded on a Bruker micrOTOF-Q mass spectrometer coupled with a HPLC Dionex UltiMate 3000 or a LTQ Orbitrap XL. NMR spectra were recorded on a Bruker Avance DRX 500 (500 MHz ^{1}H NMR, 126 MHz ^{13}C NMR) and a Bruker Avance III 600 (600 MHz ^{1}H NMR, 151 MHz ^{13}C NMR). Chemical shifts are given in parts per million (ppm) referring to the signal center using the solvent peaks for reference, DMSO-d_6 (2.49/39.7). The multiplicity of each signal is reported as singlet (s), doublet (d), triplet (t), multiplet (m) or combinations thereof. Multiplicities and coupling constants are reported as measured and might disagree with the expected values. Due to the well-known phenomenon of cis/trans-amide bond rotamers in peptoids,[8] ^{1}H and ^{13}C NMR signals can occur as two distinct sets of signals. ^{1}H NMR signals marked with an asterisk (*) correspond to peaks assigned to the minor rotamer conformation. Due to solvent exchange C-N*H*-OH signals are partly not detectable in ^{1}H NMR spectra.

3.2. General Procedures

General Procedure A (Amide coupling on resin)

This procedure was performed as previously published.[7,9] This reaction was performed in a scale of 0.21 – 3.72 mmol. The preloaded resin **3** (loading: 0.618 – 0.681 mmol/g, prepared according to our previously published protocol[7,9]) was swelled in DMF for 1 h. After deprotection of the Fmoc group by treatment with the deprotection solution (20% piperidine in DMF) two times for 5 min, the resin was washed with DMF (4 × 5 mL), MeOH (4 × 5 mL) and DMF (4 × 5 mL). In parallel, the respective Fmoc-protected acid (2.00 eq), HATU (2.00 eq), HOBt*H_2O (2.00 eq), and DIPEA (3.00 eq) were dissolved in DMF and stirred for 5 min. This solution was added to the resin and the amide coupling was performed for 18 h. Subsequently, the resin was washed with DMF (5 × 5 mL) and CH_2Cl_2 (10 × 5 mL) and the completion of the reaction was confirmed *via* TNBS test, conducted according to manufacturer´s protocol, and by HPLC analysis after test cleavage (**general procedure C**). The modified resin was dried *in vacuo*.

General Procedure B (Ugi four-component reaction on resin)

The Ugi four-component reaction on resin was performed in a scale of 0.08-0.12 mmol. The preloaded resin was swelled and deprotected as described in **general procedure A**. The resin was washed with DMF (4 × 5 mL), MeOH (4 × 5 mL) and DMF/MeOH (1/1 *v/v*, 4 × 5 mL). In parallel, 3,4-dichlorobenzaldehyde (4.00 eq.), 2-chloroacetic acid (4.00 eq.) and benzyl isocyanide (4.00 eq.) was dissolved in DMF/MeOH (1/1 *v/v*). The solution was added to the resin and the mixture was shaken for 72 h. Subsequently, the resin was washed with DMF (5 × 5 mL) and CH_2Cl_2 (10 × 5 mL) and the completion of the reaction was confirmed *via* TNBS test, conducted according to manufacturer´s protocol, and by HPLC analysis after test cleavage (**general procedure C**). The modified resin was dried *in vacuo*.

General Procedure C (cleavage from resin)

This procedure was performed as previously published.[7,9] For confirmation of completion of the reaction, dried resin (2-3 mg) was shaken with the cleavage solution (CH_2Cl_2/TFA, 95/5 *v/v*; 100 µL/mg) for 1 h at room temperature. After filtration, the solution was adjusted to 1 mL with MeCN and analyzed by HPLC.

The large scale cleavage for purification of the final compounds was carried out in the same way using CH_2Cl_2/TFA/TIPS, 90/5/5 (*v/v/v*), but after 1 h of incubation the cleavage solution was removed under reduced pressure and the crude product was dissolved in DMSO/acetone (15/85 *v/v*) for purification by preparative HPLC.

3.3. Preparation of Compounds

4-(4-{*N*-[2-(Benzylamino)-1-(3,4-dichlorophenyl)-2-oxoethyl]-2-chloroacetamido}cyclohexane-1-carboxamido)-*N*-[7-(hydroxyamino)-7-oxoheptyl]benzamide·TFA (1a)

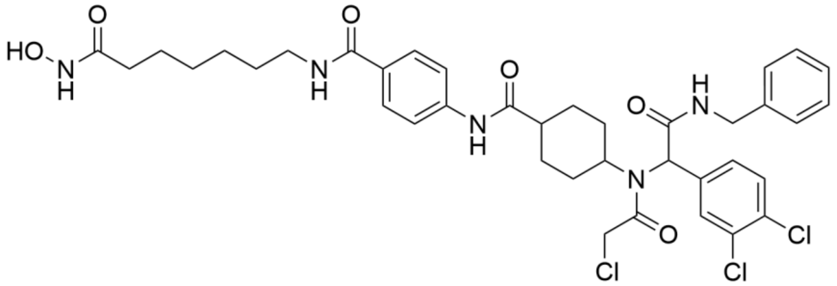

Fmoc-protected 4-aminobenzoic acid (0.96 g, 2.60 mmol, 2.00 eq) was coupled to **3** (2.11 g, 1.30 mmol, 1.00 eq) according to **general procedure A** using HATU (0.99 g, 2.60 mmol, 2.00 eq), HOBt*H_2O (0.40 g, 2.60 mmol, 2.00 eq), and DIPEA (680 µL, 3.90 mmol, 3.00 eq) dissolved in DMF (3.3 mL). The second coupling cycle was performed according to **general procedure A** using the resin bound precursor **4** (300 mg, 0.17 mmol, 1.00 eq), Fmoc-protected 4-aminocyclohexane-1-carboxylic acid (125 mg, 0.34 mmol, 2.00 eq), HATU (130 mg, 0.34 mmol, 2.00 eq), HOBt*H_2O (52 mg, 0.34 mmol, 2.00 eq), and DIPEA (89 µL, 0.51 mmol, 3.00 eq) dissolved in DMF (0.4 mL). Subsequently, the Ugi four-component reaction was performed according to **general procedure B** using the resin bound precursor **5a** (145 mg, 0.08 mmol, 1.00 eq), 3,4-dichlorobenzaldehyde (57 mg, 0.32 mmol, 4.00 eq), 2-chloroacetic acid (30 mg, 0.32 mmol, 4.00 eq), and benzyl isocyanide (45 µL, 0.32 mmol, 4.00 eq) in DMF/MeOH (1/1 *v*/*v*, 0.3 mL). Cleavage was performed according to **general procedure C** and purification by preparative HPLC afforded the TFA salt of **1a** as an amorphous white powder (20 mg, 22 µmol).

Yield 28%; **^{1}H NMR** (500 MHz, DMSO-d_6) δ = 10.31 (s, 1H), 10.03/9.93* (s/s, 1H), 8.30 – 8.22 (m, 1H), 7.82 – 7.47 (m, 7H), 7.46 – 7.15 (m, 6H), 5.19/4.99* (s/s, 1H), 4.82 – 4.61 (m, 1H), 4.48 – 4.21 (m, 3H), 3.87 – 3.73 (m, 2H), 3.25 – 3.17 (m, 2H), 2.73 – 2.55 (m, 1H), 2.33 – 2.21 (m, 1H), 2.12 – 1.77 (m, 6H), 1.58 – 1.37 (m, 6H), 1.33 – 1.22 (m, 4H); **^{13}C NMR** (126 MHz, DMSO-d_6) δ = 174.0, 169.1, 167.8, 167.6, 165.5, 158.1 (q, ^2J = 36.6 Hz, TFA), 141.7, 139.2, 138.5, 129.8, 128.9, 128.8, 128.6, 128.5, 128.0, 127.8, 127.8, 127.0, 126.5, 118.5, 60.1, 59.8, 57.3, 43.4, 42.7, 40.1, 32.2, 30.5, 29.0, 28.5, 28.3, 27.8, 27.0, 26.2, 25.0. **HRMS (ESI)** *m/z* $[M+H]^+$ calcd for $C_{38}H_{44}Cl_3N_5O_6$ 772.2430, found 772.2445; **HPLC** (gradient B), t_R = 15.50 min, 96.7% purity.

4-[(1*r*,4*r*)-4-({*N*-[2-(Benzylamino)-1-(3,4-dichlorophenyl)-2-oxoethyl]-2-chloroacetamido}methyl) cyclohexane-1-carboxamido]-*N*-[7-(hydroxyamino)-7-oxoheptyl]benzamide (1b)

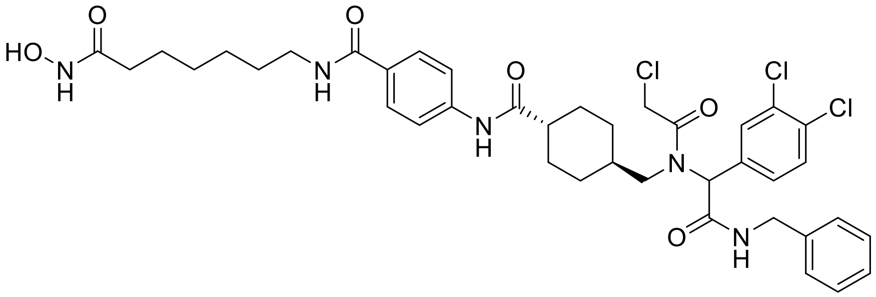

Fmoc-protected 4-aminobenzoic acid (0.96 g, 2.60 mmol, 2.00 eq) was coupled to **3** (2.11 g, 1.30 mmol, 1.00 eq) according to **general procedure A** using HATU (0.99 g, 2.60 mmol, 2.00 eq), HOBt*H_2O (0.40 g, 2.60 mmol, 2.00 eq), and DIPEA (680 µL, 3.90 mmol, 3.00 eq) dissolved in DMF (3.3 mL). The second coupling cycle was performed according to **general procedure A** using the resin bound precursor **4** (300 mg, 0.17 mmol, 1.00 eq), Fmoc-4-AMCHC-OH (129 mg, 0.34 mmol, 2.00 eq), HATU (130 mg, 0.34 mmol, 2.00 eq), HOBt*H_2O (52 mg, 0.34 mmol, 2.00 eq), and DIPEA (89 µL, 0.51 mmol, 3.00 eq) dissolved in DMF (0.4 mL). Subsequently, the Ugi four-component reaction was performed according to **general procedure B** using the resin bound precursor **5b** (142 mg, 0.08 mmol, 1.00 eq), 3,4-dichlorobenzaldehyde (57 mg, 0.32 mmol, 4.00 eq), 2-chloroacetic acid (30 mg, 0.32 mmol, 4.00 eq), and benzyl isocyanide (45 µL, 0.32 mmol, 4.00 eq) in DMF/MeOH (1/1 *v*/*v*, 0.3 mL). Cleavage was performed according to **general procedure C** and purification by preparative HPLC afforded **1b** as an amorphous white powder (6 mg, 8 µmol).

Yield 10%; **^{1}H NMR** (600 MHz, DMSO-d_6) δ = 10.30 (s, 1H), 9.98 (s, 1H), 8.85*/8.26 (s/t, J = 5.6 Hz, 1H), 7.76 (d, J = 8.6 Hz, 2H), 7.73 – 7.57 (m, 4H), 7.45 – 7.19 (m, 6H), 5.63*/5.52 (s/s, 1H), 4.53 – 4.25 (m, 4H), 3.32 – 3.14 (m, 4H), 2.28 – 2.11 (m, 2H), 1.93 (t, J = 7.4 Hz, 2H), 1.85 – 1.68 (m, 2H), 1.67 – 1.44 (m, 6H), 1.32 – 1.10 (m, 6H), 0.99 – 0.80 (m, 2H); **^{13}C NMR** (151 MHz, DMSO-d_6) δ = 174.3, 169.1, 167.8, 166.9, 165.5, 141.8, 139.2, 137.6, 131.6, 130.7, 130.6, 130.3, 130.0, 128.9, 128.2, 127.8, 127.1, 126.7, 118.1, 63.3, 53.5, 44.6, 42.7, 42.4, 40.1, 38.9, 36.3, 32.2, 29.1, 28.8, 28.6, 28.5, 28.3, 26.2, 25.1. **HRMS (ESI)** *m/z* $[M+H]^+$ calcd for $C_{39}H_{46}Cl_3N_5O_6$ 786.2586, found 786.2627; **HPLC** (gradient B), t_R = 15.54 min, 96.0% purity.

4-[5-(2-Chloroacetyl)-4-(3,4-dichlorophenyl)-3-oxo-1-phenyl-8,11-dioxa-2,5-diazatetradecan-14-amido]-*N*-[7-(hydroxyamino)-7-oxoheptyl]benzamide·TFA (1c)

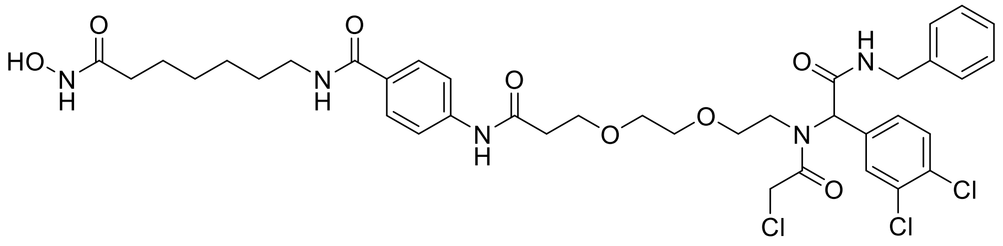

Fmoc-protected 4-aminobenzoic acid (0.96 g, 2.60 mmol, 2.00 eq) was coupled to **3** (2.11 g, 1.30 mmol, 1.00 eq) according to **general procedure A** using HATU (0.99 g, 2.60 mmol, 2.00 eq), HOBt*H_2O (0.40 g, 2.60 mmol, 2.00 eq), and DIPEA (680 µL, 3.90 mmol, 3.00 eq) dissolved in DMF (3.3 mL). The second coupling cycle was performed according to **general procedure A** using the resin bound precursor **4** (300 mg, 0.17 mmol, 1.00 eq), Fmoc-*N*-amido-PEG2-acid (136 mg, 0.34 mmol, 2.00 eq), HATU (130 mg, 0.34 mmol, 2.00 eq), HOBt*H_2O (52 mg, 0.34 mmol, 2.00 eq), and DIPEA (89 µL, 0.51 mmol, 3.00 eq) dissolved in DMF (0.4 mL). Subsequently, the Ugi four-component reaction was performed according to **general procedure B** using the resin bound precursor **5c** (163 mg, 0.08 mmol, 1.00 eq), 3,4-dichlorobenzaldehyde (58 mg, 0.32 mmol, 4.00 eq), 2-chloroacetic acid (31 mg, 0.32 mmol, 4.00 eq), and benzyl isocyanide (45 µL, 0.32 mmol, 4.00 eq) in DMF/MeOH (1/1 *v*/*v*, 0.4 mL). Cleavage was performed according to **general procedure C** and purification by preparative HPLC afforded the TFA salt of **1c** as an amorphous white powder (33 mg, 36 µmol).

Yield 44%; **^{1}H NMR** (600 MHz, DMSO-d_6) δ = 10.31 (s, 1H), 10.10 (s, 1H), 8.85*/8.53 (s/t, J = 6.0 Hz, 1H), 8.28 (t, J = 5.6 Hz, 1H), 7.78 (d, J = 8.5 Hz, 2H), 7.64 (d, J = 8.5 Hz, 2H), 7.62 (d, J = 8.4 Hz, 1H), 7.51 (s, 1H), 7.36 – 7.19 (m, 6H), 5.77/5.65* (s/s, 1H), 4.65 – 4.50 (m, 2H), 4.35 – 4.27 (m, 2H), 3.65 (t, J = 6.3 Hz, 2H), 3.58 – 3.51 (m, 2H), 3.44 – 3.39 (m, 2H), 3.35 – 3.17 (m, 6H), 2.56 (t, J = 6.2 Hz, 2H), 1.94 (t, J = 7.4 Hz, 2H), 1.53 – 1.45 (m, 4H), 1.32 – 1.22 (m, 4H); **^{13}C NMR** (151 MHz, DMSO-d_6) δ = 169.4, 169.1, 167.8, 167.3, 165.5, 158.2 (q, ^2J = 37.0 Hz, TFA), 141.5, 139.0, 137.3, 131.0, 130.8, 130.6, 130.4, 129.5, 129.1, 128.2, 127.9, 127.2, 126.8, 118.1, 114.5 (q, ^1J = 290.8 Hz, TFA), 69.7, 69.3, 68.7, 66.5, 61.7, 46.3, 42.8, 42.4, 40.1, 37.1, 32.2, 29.1, 28.3, 26.2, 25.1. **HRMS (ESI)** *m/z* $[M+H]^+$ calcd for $C_{38}H_{46}Cl_3N_5O_8$ 806.2485, found 806.2478; **HPLC** (gradient A), t_R = 12.08 min, 99.2% purity.

4-[5-(2-Chloroacetyl)-4-(3,4-dichlorophenyl)-3-oxo-1-phenyl-8,11,14-trioxa-2,5-diazaheptadecan-17-amido]-*N*-[7-(hydroxyamino)-7-oxoheptyl]benzamide·TFA (1d)

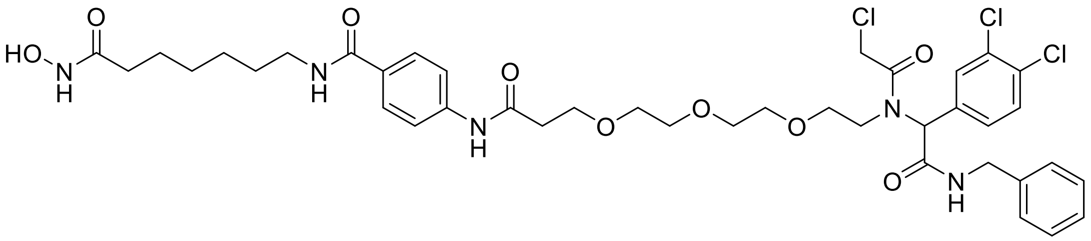

Fmoc-protected 4-aminobenzoic acid (0.96 g, 2.60 mmol, 2.00 eq) was coupled to **3** (2.11 g, 1.30 mmol, 1.00 eq) according to **general procedure A** using HATU (0.99 g, 2.60 mmol, 2.00 eq), HOBt*H_2O (0.40 g, 2.60 mmol, 2.00 eq), and DIPEA (680 µL, 3.90 mmol, 3.00 eq) dissolved in DMF (3.3 mL). The second coupling cycle was performed according to **general procedure A** using the resin bound precursor **4** (300 mg, 0.17 mmol, 1.00 eq), Fmoc-*N*-amido-PEG3-acid (151 mg, 0.34 mmol, 2.00 eq), HATU (130 mg, 0.34 mmol, 2.00 eq), HOBt*H_2O (52 mg, 0.34 mmol, 2.00 eq), and DIPEA (89 µL, 0.51 mmol, 3.00 eq) dissolved in DMF (0.4 mL). Subsequently, the Ugi four-component reaction was performed according to **general procedure B** using the resin bound precursor **5d** (170 mg, 0.08 mmol, 1.00 eq), 3,4-dichlorobenzaldehyde (60 mg, 0.33 mmol, 4.00 eq), 2-chloroacetic acid (32 mg, 0.33 mmol, 4.00 eq), and benzyl isocyanide (46 µL, 0.33 mmol, 4.00 eq) in DMF/MeOH (1/1 *v*/*v*, 0.4 mL). Cleavage was performed according to **general procedure C** and purification by preparative HPLC afforded the TFA salt of **1d** as an amorphous white powder (35 mg, 36 µmol).

Yield 43%; **^{1}H NMR** z(500 MHz, DMSO-d_6) δ = 10.31 (s, 1H), 10.10 (s, 1H), 8.84*/8.51 (s/t, J = 6.0 Hz, 1H), 8.27 (t, J = 5.6 Hz, 1H), 7.78 (d, J = 8.7 Hz, 2H), 7.68 – 7.48 (m, 4H), 7.36 – 7.18 (m, 6H), 5.77/5.66* (s/s, 1H), 4.66 – 4.52 (m, 2H), 4.36 – 4.28 (m, 2H), 3.69 (t, J = 6.2 Hz, 2H), 3.62 – 3.12 (m, 14H), 2.57 (t, J = 6.3 Hz, 2H), 1.94 (t, J = 7.4 Hz, 2H), 1.55 – 1.44 (m, 4H), 1.34 – 1.21 (m, 4H); **^{13}C NMR** (126 MHz, DMSO-d_6) δ = 169.5, 169.1, 167.8, 167.3, 165.5, 158.2 (q, ^2J = 37.3 Hz, TFA), 141.5, 139.0, 137.3, 131.1, 130.8, 130.6, 130.4, 129.5, 129.1, 128.2, 127.9, 127.2, 126.8, 118.1, 115.2 (q, ^1J = 290.3 Hz, TFA), 69.7, 69.6, 69.6, 69.5, 68.6, 66.5, 61.7, 46.4, 42.8, 42.4, 40.1, 37.2, 32.2, 29.1, 28.3, 26.2, 25.1. **HRMS (ESI)** *m/z* $[M+H]^+$ calcd for $C_{40}H_{50}Cl_3N_5O_9$ 850.2747, found 850.2751; **HPLC** (gradient A), t_R = 12.09 min, 98.9% purity.

4-[5-(2-Chloroacetyl)-4-(3,4-dichlorophenyl)-3-oxo-1-phenyl-8,11,14,17-tetraoxa-2,5-diazaicosan-20-amido]-*N*-[7-(hydroxyamino)-7-oxoheptyl]benzamide (1e)

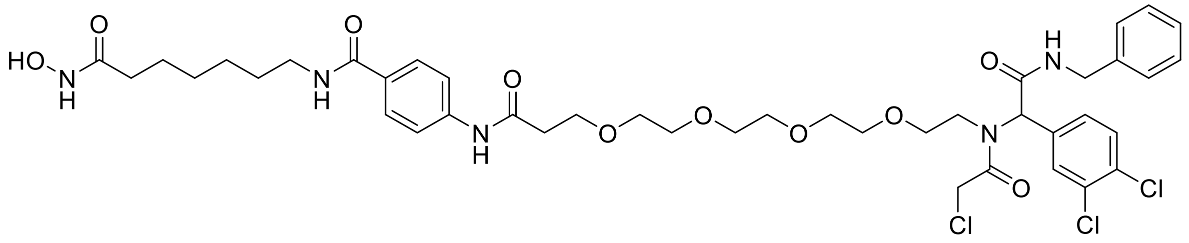

Fmoc-protected 4-aminobenzoic acid (0.96 g, 2.60 mmol, 2.00 eq) was coupled to **3** (2.11 g, 1.30 mmol, 1.00 eq) according to **general procedure A** using HATU (0.99 g, 2.60 mmol, 2.00 eq), HOBt*H_2O (0.40 g, 2.60 mmol, 2.00 eq), and DIPEA (680 µL, 3.90 mmol, 3.00 eq) dissolved in DMF (3.3 mL). The second coupling cycle was performed according to **general procedure A** using the resin bound precursor **4** (300 mg, 0.17 mmol, 1.00 eq), Fmoc-*N*-amido-PEG4-acid (166 mg, 0.34 mmol, 2.00 eq), HATU (130 mg, 0.34 mmol, 2.00 eq), HOBt*H_2O (52 mg, 0.34 mmol, 2.00 eq), and DIPEA (89 µL, 0.51 mmol, 3.00 eq) dissolved in DMF (0.4 mL). Subsequently, the Ugi four-component reaction was performed according to **general procedure B** using the resin bound precursor **5e** (184 mg, 0.09 mmol, 1.00 eq), 3,4-dichlorobenzaldehyde (61 mg, 0.34 mmol, 4.00 eq), 2-chloroacetic acid (32 mg, 0.34 mmol, 4.00 eq), and benzyl isocyanide (47 µL, 0.34 mmol, 4.00 eq) in DMF/MeOH (1/1 *v*/*v*, 0.4 mL). Cleavage was performed according to **general procedure C** and purification by preparative HPLC afforded **1e** as an amorphous white powder (43 mg, 48 µmol).

Yield 57%; **^{1}H NMR** (600 MHz, DMSO-d_6) δ = 10.31 (s, 1H), 10.11 (s, 1H), 8.63*/8.51 (s/t, J = 6.0 Hz, 1H), 8.28 (t, J = 5.6 Hz, 1H), 7.78 (d, J = 8.7 Hz, 2H), 7.67 – 7.49 (m, 4H), 7.36 – 7.19 (m, 6H), 5.77/5.65* (s/s, 1H), 4.65 – 4.54 (m, 2H), 4.38 – 4.27 (m, 2H), 3.70 (t, J = 6.2 Hz, 2H), 3.63 – 3.34 (m, 14H), 3.28 – 3.16 (m, 4H), 2.57 (t, J = 6.3 Hz, 2H), 1.94 (t, J = 7.4 Hz, 2H), 1.53 – 1.45 (m, 4H), 1.33 – 1.22 (m, 4H); **^{13}C NMR** (151 MHz, DMSO-d_6) δ = 169.5, 169.1, 167.8, 167.3, 165.5, 141.5, 139.0, 137.3, 131.1, 130.8, 130.6, 130.4, 129.5, 129.1, 128.2, 127.9, 127.2, 126.8, 118.1, 69.7, 69.7, 69.7, 69.6, 69.6, 69.5, 68.6, 66.5, 61.7, 46.4, 42.8, 42.4, 40.1, 37.2, 32.2, 29.1, 28.3, 26.2, 25.1. **HRMS (ESI)** *m/z* $[M+H]^+$ calcd for $C_{42}H_{54}Cl_3N_5O_{10}$ 894.3009, found 894.3009; **HPLC** (gradient A), t_R = 12.08 min, 97.9% purity.

4-(2-{*N*-[2-(Benzylamino)-1-(3,4-dichlorophenyl)-2-oxoethyl]-2-chloroacetamido}acetamido)-*N*-[7-(hydroxyamino)-7-oxoheptyl]benzamide (1f)

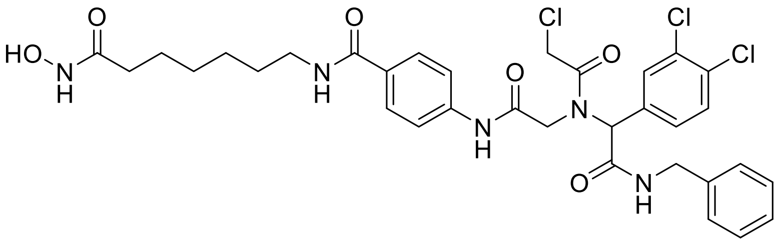

Fmoc-protected 4-aminobenzoic acid (1.38 g, 3.72 mmol, 2.00 eq) was coupled to **3** (2.73 g, 1.86 mmol, 1.00 eq) according to **general procedure A** using HATU (1.41 g, 3.72 mmol, 2.00 eq), HOBt*H_2O (0.57 g, 3.72 mmol, 2.00 eq), and DIPEA (971 µL, 5.58 mmol, 3.00 eq) dissolved in DMF (4.67 mL). The second coupling cycle was performed according to **general procedure A** using the resin bound precursor **4** (308 mg, 0.21 mmol, 1.00 eq), Fmoc-Gly-OH (130 mg, 0.43 mmol, 2.00 eq), HATU (163 mg, 0.43 mmol, 2.00 eq), HOBt*H_2O (66 mg, 0.43 mmol, 2.00 eq), and DIPEA (112 µL, 0.64 mmol, 3.00 eq) dissolved in DMF (0.5 mL). Subsequently, the Ugi four-component reaction was performed according to **general procedure B** using the resin bound precursor **5f** (160 mg, 0.10 mmol, 1.00 eq), 3,4-dichlorobenzaldehyde (74 mg, 0.41 mmol, 4.00 eq), 2-chloroacetic acid (39 mg, 0.41 mmol, 4.00 eq), and benzyl isocyanide (58 µL, 0.41 mmol, 4.00 eq) in DMF/MeOH (1/1 *v*/*v*, 0.4 mL). Cleavage was performed according to **general procedure C** and purification by preparative HPLC afforded **1f** as an amorphous white powder (24 mg, 35 µmol).

Yield 36%; **^{1}H NMR** (500 MHz, DMSO-d_6) δ = 10.30 (s, 1H), 10.11 (s, 1H), 9.17*/8.94 (s/t, J = 5.9 Hz, 1H), 8.28 (t, J = 5.6 Hz, 1H), 7.76 (d, J = 8.8 Hz, 2H), 7.54 (d, J = 8.3 Hz, 1H), 7.49 – 7.39 (m, 3H), 7.38 – 7.27 (m, 3H), 7.26 – 7.18 (m, 3H), 6.16/5.83* (s/s, 1H), 4.59 – 4.45 (m, 2H), 4.42 – 4.23 (m, 4H), 3.25 – 3.18 (m, 2H), 1.94 (t, J = 7.4 Hz, 2H), 1.53 – 1.44 (m, 4H), 1.33 – 1.22 (m, 4H); **^{13}C NMR** (126 MHz, DMSO-d_6) δ = 169.1, 168.0, 167.8, 167.1, 165.4, 140.7, 138.7, 136.1, 131.1, 131.0, 131.0, 130.4, 129.7, 129.4, 128.3, 127.8, 127.3, 126.9, 118.3, 59.6, 48.8, 42.8, 42.3, 40.1, 32.2, 29.0, 28.3, 26.2, 25.0. **HRMS (ESI)** *m/z* $[M+H]^+$ calcd for $C_{33}H_{36}Cl_3N_5O_6$ 704.1804, found 704.1769; **HPLC** (gradient A), t_R = 11.93 min, 97.9% purity.

4-(4-{*N*-[2-(Benzylamino)-1-(3,4-dichlorophenyl)-2-oxoethyl]-2-chloroacetamido}butanamido)-*N*-[7-(hydroxyamino)-7-oxoheptyl]benzamide (1g)

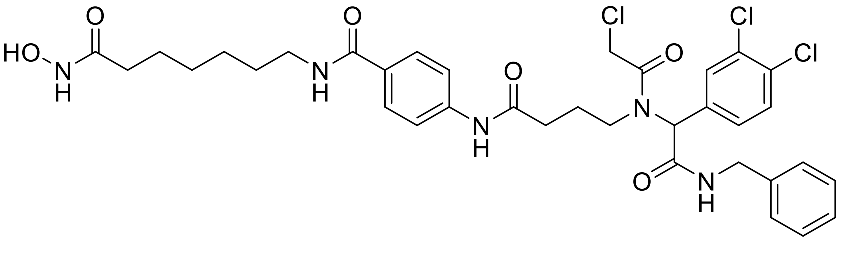

Fmoc-protected 4-aminobenzoic acid (1.38 g, 3.72 mmol, 2.00 eq) was coupled to **3** (2.73 g, 1.86 mmol, 1.00 eq) according to **general procedure A** using HATU (1.41 g, 3.72 mmol, 2.00 eq), HOBt*H_2O (0.57 g, 3.72 mmol, 2.00 eq), and DIPEA (971 µL, 5.58 mmol, 3.00 eq) dissolved in DMF (4.67 mL). The second coupling cycle was performed according to **general procedure A** using the resin bound precursor **4** (308 mg, 0.21 mmol, 1.00 eq), Fmoc-GABA-OH (142 mg, 0.42 mmol, 2.00 eq), HATU (159 mg, 0.42 mmol, 2.00 eq), HOBt*H_2O (64 mg, 0.42 mmol, 2.00 eq), and DIPEA (109 µL, 0.63 mmol, 3.00 eq) dissolved in DMF (0.5 mL). Subsequently, the Ugi four-component reaction was performed according to **general procedure B** using the resin bound precursor **5g** (163 mg, 0.10 mmol, 1.00 eq), 3,4-dichlorobenzaldehyde (74 mg, 0.41 mmol, 4.00 eq), 2-chloroacetic acid (39 mg, 0.41 mmol, 4.00 eq), and benzyl isocyanide (58 µL, 0.41 mmol, 4.00 eq) in DMF/MeOH (1/1 *v*/*v*, 0.4 mL). Cleavage was performed according to **general procedure C** and purification by preparative HPLC afforded **1g** as an amorphous white powder (39 mg, 53 µmol).

Yield 55%; **^{1}H NMR** (600 MHz, DMSO-d_6) δ = 10.31 (s, 1H), 10.01 (s, 1H), 8.84*/8.59 (s/t, J = 6.0 Hz, 1H), 8.28 (t, J = 5.7 Hz, 1H), 7.78 (d, J = 8.4 Hz, 2H), 7.64 – 7.49 (m, 4H), 7.35 – 7.19 (m, 6H), 5.83/5.67* (s/s, 1H), 4.61 – 4.44 (m, 2H), 4.41 – 4.25 (m, 2H), 3.41 – 3.30 (m, 2H), 3.25 – 3.19 (m, 2H), 2.21 (d, J = 7.0 Hz, 2H), 1.94 (t, J = 7.4 Hz, 2H), 1.82 – 1.36 (m, 6H), 1.33 – 1.22 (m, 4H); **^{13}C NMR** (151 MHz, DMSO-d_6) δ = 170.7, 169.1, 167.9, 166.8, 165.5, 141.5, 139.0, 137.1, 131.2, 131.0, 130.7, 130.5, 129.6, 129.0, 128.2, 127.9, 127.2, 126.8, 118.1, 61.2, 45.9, 42.4, 42.4, 40.1, 33.0, 32.2, 29.1, 28.3, 26.2, 25.1, 24.9. **HRMS (ESI)** *m/z* $[M+H]^+$ calcd for $C_{35}H_{40}Cl_3N_5O_6$ 732.2117, found 732.2094; **HPLC** (gradient A), t_R = 11.98 min, 98.7% purity.

4-(6-{*N*-[2-(Benzylamino)-1-(3,4-dichlorophenyl)-2-oxoethyl]-2-chloroacetamido}hexanamido)-*N*-[7-(hydroxyamino)-7-oxoheptyl]benzamide·TFA (1h)

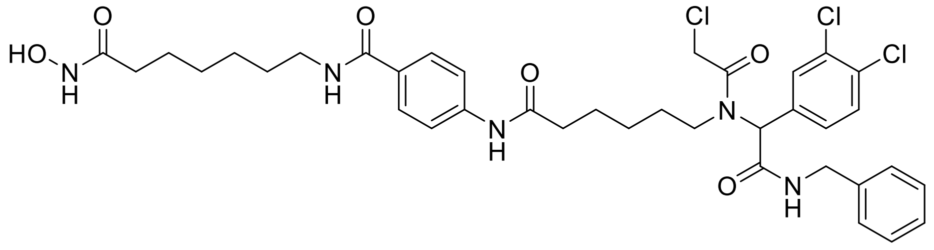

Fmoc-protected 4-aminobenzoic acid (1.38 g, 3.72 mmol, 2.00 eq) was coupled to **3** (2.73 g, 1.86 mmol, 1.00 eq) according to **general procedure A** using HATU (1.41 g, 3.72 mmol, 2.00 eq), HOBt*H_2O (0.57 g, 3.72 mmol, 2.00 eq), and DIPEA (971 µL, 5.58 mmol, 3.00 eq) dissolved in DMF (4.67 mL). The second coupling cycle was performed according to **general procedure A** using the resin bound precursor **4** (302 mg, 0.21 mmol, 1.00 eq), Fmoc-6-Ahx-OH (148 mg, 0.41 mmol, 2.00 eq), HATU (156 mg, 0.41 mmol, 2.00 eq), HOBt*H_2O (63 mg, 0.41 mmol, 2.00 eq), and DIPEA (107 µL,

0.62 mmol, 3.00 eq) dissolved in DMF (0.5 mL). Subsequently, the Ugi four-component reaction was performed according to **general procedure B** using the resin bound precursor **5h** (164 mg, 0.10 mmol, 1.00 eq), 3,4-dichlorobenzaldehyde (74 mg, 0.41 mmol, 4.00 eq), 2-chloroacetic acid (39 mg, 0.41 mmol, 4.00 eq), and benzyl isocyanide (58 µL, 0.41 mmol, 4.00 eq) in DMF/MeOH (1/1 *v*/*v*, 0.4 mL). Cleavage was performed according to **general procedure C** and purification by preparative HPLC afforded the TFA salt of **1h** as an amorphous white powder (38 mg, 44 µmol).

Yield 45%; ^{1}**H NMR** (600 MHz, DMSO-d_6) δ = 10.31 (s, 1H), 10.01 (s, 1H), 8.86*/8.57 (s/t, *J* = 6.0 Hz, 1H), 8.27 (t, *J* = 5.6 Hz, 1H), 7.77 (d, *J* = 8.7 Hz, 2H), 7.68 – 7.61 (m, 3H), 7.52 (s, 1H), 7.37 – 7.16 (m, 6H), 5.81/5.65* (s/s, 1H), 4.55 – 4.24 (m, 4H), 3.31 (t, *J* = 7.4 Hz, 2H), 3.25 – 3.18 (m, 2H), 2.22 (t, *J* = 7.5 Hz, 2H), 1.94 (t, *J* = 7.4 Hz, 2H), 1.53 – 1.02 (m, 14H); ^{13}**C NMR** (151 MHz, DMSO-d_6) δ = 171.4, 169.1, 167.9, 166.6, 165.5, 158.0 (q, ^{2}J = 34.4 Hz, TFA), 141.7, 139.1, 137.3, 131.1, 130.9, 130.7, 130.5, 129.6, 128.9, 128.2, 127.9, 127.2, 126.8, 118.1, 61.3, 46.5, 42.6, 42.4, 40.1, 36.3, 32.2, 29.1, 28.9, 28.3, 26.2, 25.8, 25.1, 24.5. **HRMS (ESI)** *m/z* $[M+H]^+$ calcd for $C_{37}H_{44}Cl_3N_5O_6$ 760.2430, found 760.2415; **HPLC** (gradient A), t_R = 12.15 min, 97.9% purity.

4-(8-{*N*-[2-(Benzylamino)-1-(3,4-dichlorophenyl)-2-oxoethyl]-2-chloroacetamido}octanamido)-*N*-[7-(hydroxyamino)-7-oxoheptyl]benzamide·TFA (1i)

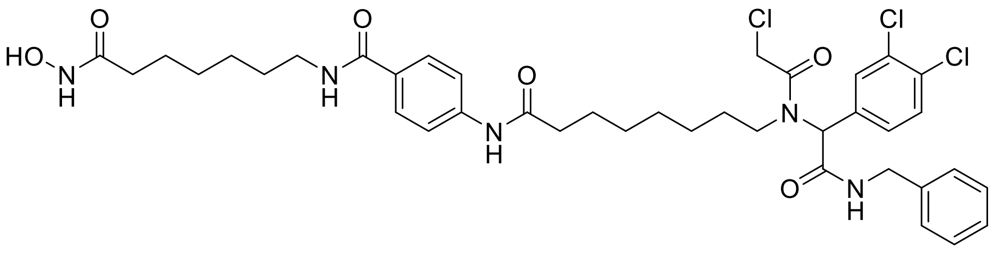

Fmoc-protected 4-aminobenzoic acid (1.01 g, 2.73 mmol, 2.00 eq) was coupled to **3** (1.99 g, 1.37 mmol, 1.00 eq) according to **general procedure A** using HATU (1.04 g, 2.73 mmol, 2.00 eq), HOBt*H_2O (0.42 g, 2.73 mmol, 2.00 eq), and DIPEA (714 µL, 4.10 mmol, 3.00 eq) dissolved in DMF (3.42 mL). The second coupling cycle was performed according to **general procedure A** using the resin bound precursor **4** (1.55 g, 0.95 mmol, 1.00 eq), Fmoc-8-Aoc-OH (0.73 g, 1.91 mmol, 2.00 eq), HATU (0.73 g, 2.91 mmol, 2.00 eq), HOBt*H_2O (0.29 g, 1.91 mmol, 2.00 eq), and DIPEA (498 µL, 2.86 mmol, 3.00 eq) dissolved in DMF (2.4 mL). Subsequently, the Ugi four-component reaction was performed according to **general procedure B** using the resin bound precursor **5i** (200 mg, 0.12 mmol, 1.00 eq), 3,4-dichlorobenzaldehyde (84 mg, 0.47 mmol, 4.00 eq), 2-chloroacetic acid (44 mg, 0.47 mmol, 4.00 eq), and benzyl isocyanide (65 µL, 0.47 mmol, 4.00 eq) in DMF/MeOH (1/1 *v*/*v*, 0.4 mL). Cleavage was performed according to **general procedure C** and purification by preparative HPLC afforded the TFA salt of **1i** as an amorphous white powder (39 mg, 47 µmol).

Yield 40%; **^{1}H NMR** (600 MHz, DMSO-d_6) δ = 10.32 (s, 1H), 10.03 (s, 1H), 8.85*/8.58 (s/t, *J* = 6.1 Hz, 1H), 8.27 (t, *J* = 5.6 Hz, 1H), 7.77 (d, *J* = 8.6 Hz, 2H), 7.64 (d, *J* = 8.5 Hz, 3H), 7.60 – 7.49 (m, 1H), 7.35 – 7.20 (m, 6H), 5.83/5.65* (s/s, 1H), 4.53 – 4.42 (m, 2H), 4.36 – 4.25 (m, 2H), 3.37 – 3.24 (m, 2H), 3.24 – 3.19 (m, 2H), 2.28 (t, *J* = 7.5 Hz, 2H), 1.94 (t, *J* = 7.4 Hz, 2H), 1.56 – 0.93 (m, 18H); **^{13}C NMR** (151 MHz, DMSO-d_6) δ = 171.5, 169.1, 167.9, 166.7, 165.5, 158.3 (q, ^2J = 37.8 Hz, TFA), 141.7, 139.1, 137.3, 131.2, 130.9, 130.7, 130.5, 129.7, 128.9, 128.2, 127.9, 127.2, 126.8, 118.1, 115.2 (q, ^1J = 289.7 Hz, TFA), 62.0, 61.2, 46.5, 42.5, 40.1, 36.4, 32.2, 29.1, 28.4, 28.3, 28.2, 26.2, 25.9, 25.5, 25.1, 24.8. **HRMS (ESI)** *m/z* $[M+H]^+$ calcd for $C_{39}H_{48}Cl_3N_5O_6$ 788.2743, found 788.2741; **HPLC** (gradient A), t_R = 12.45 min, 98.7% purity.

4-(12-{*N*-[2-(Benzylamino)-1-(3,4-dichlorophenyl)-2-oxoethyl]-2-chloroacetamido} dodecanamido)-*N*-[7-(hydroxyamino)-7-oxoheptyl]benzamide (1j)

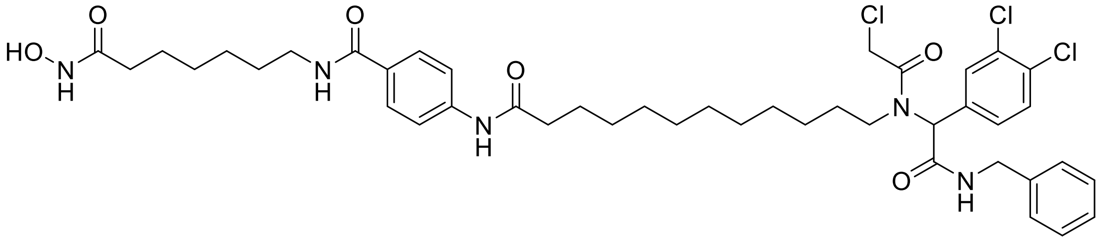

Fmoc-protected 4-aminobenzoic acid (1.38 g, 3.72 mmol, 2.00 eq) was coupled to **3** (2.73 g, 1.86 mmol, 1.00 eq) according to **general procedure A** using HATU (1.41 g, 3.72 mmol, 2.00 eq), HOBt*H_2O (0.57 g, 3.72 mmol, 2.00 eq), and DIPEA (971 µL, 5.58 mmol, 3.00 eq) dissolved in DMF (4.67 mL). The second coupling cycle was performed according to **general procedure A** using the resin bound precursor **4** (304 mg, 0.21 mmol, 1.00 eq), Fmoc-12-Ado-OH (186 mg, 0.41 mmol, 2.00 eq), HATU (156 mg, 0.41 mmol, 2.00 eq), HOBt*H_2O (63 mg, 0.41 mmol, 2.00 eq), and DIPEA (107 µL, 0.62 mmol, 3.00 eq) dissolved in DMF (0.5 mL). Subsequently, the Ugi four-component reaction was performed according to **general procedure B** using the resin bound precursor **5j** (170 mg, 0.10 mmol, 1.00 eq), 3,4-dichlorobenzaldehyde (74 mg, 0.41 mmol, 4.00 eq), 2-chloroacetic acid (39 mg, 0.41 mmol, 4.00 eq), and benzyl isocyanide (58 µL, 0.41 mmol, 4.00 eq) in DMF/MeOH (1/1 *v*/*v*, 0.4 mL). Cleavage was performed according to **general procedure C** and purification by preparative HPLC afforded **1j** as an amorphous white powder (34 mg, 40 µmol).

Yield 43%; **^{1}H NMR** (600 MHz, DMSO-d_6) δ = 10.31 (s, 1H), 10.03 (s, 1H), 8.83*/8.58 (s/t, *J* = 6.0 Hz, 1H), 8.27 (t, *J* = 5.7 Hz, 1H), 7.77 (d, *J* = 8.4 Hz, 2H), 7.69 – 7.61 (m, 3H), 7.59 – 7.49 (m, 1H), 7.35 – 7.19 (m, 6H), 5.84/5.65* (s/s, 1H), 4.54 – 4.40 (m, 2H), 4.38 – 4.25 (m, 2H), 3.25 – 3.18 (m, 2H), 2.31 (t, *J* = 7.4 Hz, 2H), 1.94 (t, *J* = 7.4 Hz, 2H), 1.66 – 0.64 (m, 28H); **^{13}C NMR** (151 MHz, DMSO-d_6) δ = 171.6, 169.1, 168.0, 166.7, 165.5, 141.7, 139.0, 137.3, 131.3, 130.9, 130.8, 130.5, 129.8, 128.9, 127.9, 127.2, 126.8, 118.1, 61.1, 46.4, 42.5, 42.4, 40.1, 36.4, 32.2, 29.1, 28.9, 28.8, 28.8, 28.7, 28.7,

28.6, 28.3, 28.2, 26.2, 25.9, 25.1, 25.0. **HRMS (ESI)** *m/z* $[M+H]^+$ calcd for $C_{43}H_{56}Cl_3N_5O_6$ 844.3369, found 844.3361; **HPLC** (gradient A), t_R = 13.61 min, 96.5% purity.

4-(12-{*N*-[2-(Benzylamino)-1-(3,4-dichlorophenyl)-2-oxoethyl]formamido}dodecanamido)-*N*-[7-(hydroxyamino)-7-oxoheptyl]benzamide (1j-nc)

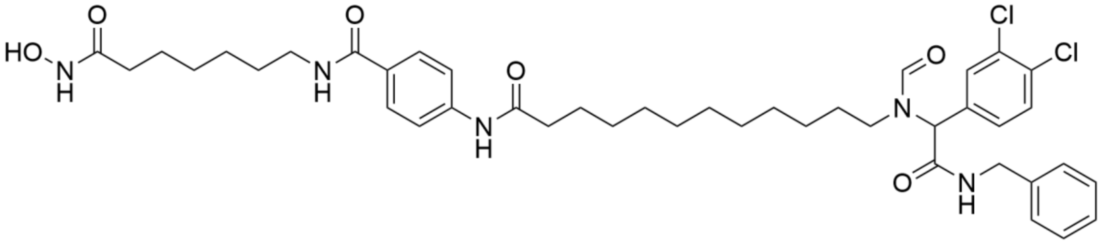

Fmoc-protected 4-aminobenzoic acid (1.38 g, 3.72 mmol, 2.00 eq) was coupled to **3** (2.73 g, 1.86 mmol, 1.00 eq) according to **general procedure A** using HATU (1.41 g, 3.72 mmol, 2.00 eq), HOBt*H_2O (0.57 g, 3.72 mmol, 2.00 eq), and DIPEA (971 µL, 5.58 mmol, 3.00 eq) dissolved in DMF (4.67 mL). The second coupling cycle was performed according to **general procedure A** using the resin bound precursor **4** (302 mg, 0.19 mmol, 1.00 eq), Fmoc-12-Ado-OH (174 mg, 0.39 mmol, 2.00 eq), HATU (147 mg, 0.39 mmol, 2.00 eq), HOBt*H_2O (59 mg, 0.39 mmol, 2.00 eq), and DIPEA (101 µL, 0.75 mmol, 3.00 eq) dissolved in DMF (0.5 mL). Subsequently, the Ugi four-component reaction was performed according to **general procedure B** with a slide modification, using the resin bound precursor **5j** (148 mg, 0.08 mmol, 1.00 eq), 3,4-dichlorobenzaldehyde (60 mg, 0.34 mmol, 4.00 eq), formic acid (13 µL, 0.34 mmol, 4.00 eq), and benzyl isocyanide (47 µL, 0.34 mmol, 4.00 eq) in DMF/MeOH (1/1 *v*/*v*, 0.3 mL). Cleavage was performed according to **general procedure C** and purification by preparative HPLC afforded **1j-nc** as an amorphous white powder (24 mg, 30 µmol).

Yield 38%; ^{1}**H NMR** (600 MHz, DMSO-d_6) δ = 10.31 (s, 1H), 10.03 (s, 1H), 8.87/8.63* (t/t, *J* = 5.8/6.0 Hz, 1H), 8.27 (t, *J* = 5.7 Hz, 1H), 8.20*/8.10 (s/s, 1H), 7.77 (d, *J* = 8.5 Hz, 2H), 7.68 – 7.62 (m, 3H), 7.55 – 7.50 (m, 1H), 7.34 – 7.19 (m, 6H), 5.75*/5.38 (s/s, 1H), 4.38 – 4.28 (m, 2H), 3.31 – 3.18 (m, 4H), 2.31 (t, *J* = 7.4 Hz, 2H), 1.94 (t, *J* = 7.4 Hz, 2H), 1.62 – 1.55 (m, 2H), 1.52 – 1.45 (m, 4H), 1.32 – 0.97 (m, 20H); ^{13}**C NMR** (151 MHz, DMSO-d_6) δ = 171.8, 169.3, 168.3, 168.0, 165.7, 163.9, 163.4, 141.9, 139.2, 138.9, 137.8, 137.5, 131.4, 131.2, 131.1, 131.1, 131.0, 130.9, 130.7, 130.6, 129.7, 129.1, 128.9, 128.5, 128.4, 128.1, 127.6, 127.4, 127.2, 127.0, 118.2, 62.4, 58.5, 46.2, 43.6, 42.8, 42.6, 40.2, 36.6, 32.4, 29.8, 29.2, 29.1, 29.0, 28.9, 28.9, 28.8, 28.7, 28.5, 27.3, 26.4, 26.4, 25.9, 25.2, 25.2f. **HRMS (ESI)** *m/z* $[M+H]^+$ calcd for $C_{42}H_{55}Cl_2N_5O_6$ 796.3602, found 796.3605; **HPLC** (gradient A), t_R = 13.43 min, 99.9% purity.

4-{*N*-[2-(Benzylamino)-1-(3,4-dichlorophenyl)-2-oxoethyl]-2-chloroacetamido}-*N*-[7-(hydroxyamino)-7-oxoheptyl]benzamide (2)

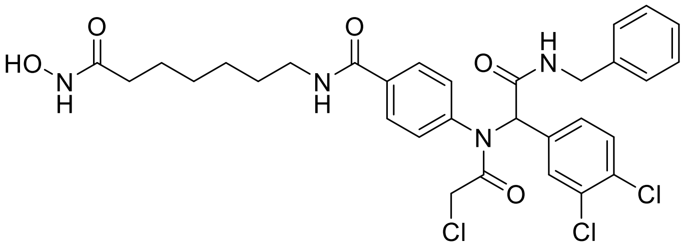

Fmoc-protected 4-aminobenzoic acid (1.38 g, 3.72 mmol, 2.00 eq) was coupled to **3** (2.73 g, 1.86 mmol, 1.00 eq) according to **general procedure A** using HATU (1.41 g, 3.72 mmol, 2.00 eq), HOBt*H_2O (0.57 g, 3.72 mmol, 2.00 eq), and DIPEA (971 µL, 5.58 mmol, 3.00 eq) dissolved in DMF (4.7 mL). Subsequently, the Ugi four-component reaction was performed according to **general procedure B** using the resin bound precursor **4** (143 mg, 0.10 mmol, 1.00 eq), 3,4-dichlorobenzaldehyde (66 mg, 0.37 mmol, 4.00 eq), 2-chloroacetic acid (35 mg, 0.37 mmol, 4.00 eq), and benzyl isocyanide (51 µL, 0.37 mmol, 4.00 eq) in DMF/MeOH (1/1 *v/v*, 0.3 mL). Cleavage was performed according to **general procedure C** and purification by preparative HPLC afforded **2** as an amorphous white powder (24 mg, 37 µmol).

Yield 42%; **^{1}H NMR** (500 MHz, DMSO-d_6) δ = 10.30 (s, 1H), 8.76 (t, J = 6.0 Hz, 1H), 8.42 (t, J = 5.6 Hz, 1H), 7.72 (d, J = 8.2 Hz, 2H), 7.46 – 7.18 (m, 9H), 7.09 – 7.01 (m, 1H), 6.08 (s, 1H), 4.40 – 4.26 (m, 2H), 4.02 – 3.91 (m, 2H), 3.24 – 3.17 (m, 2H), 1.93 (t, J = 7.4 Hz, 2H), 1.51 – 1.44 (m, 4H), 1.30 – 1.21 (m, 4H); **^{13}C NMR** (126 MHz, DMSO-d_6) δ = 169.1, 168.4, 165.4, 164.9, 140.4, 139.0, 135.3, 134.3, 132.2, 130.8, 130.5, 130.5, 130.4, 130.1, 128.2, 127.7, 127.1, 126.8, 63.3, 42.9, 42.4, 40.1, 32.2, 28.9, 28.3, 26.2, 25.0. **HRMS (ESI)** *m/z* $[M+H]^+$ calcd for $C_{31}H_{33}Cl_3N_4O_5$ 647.1589, found 647.1568; **HPLC** (gradient A), t_R = 12.07 min, 97.1% purity.

***N*-benzyl-2-(2-chloro-*N*-propylacetamido)-2-(3,4-dichlorophenyl)acetamide (6)**

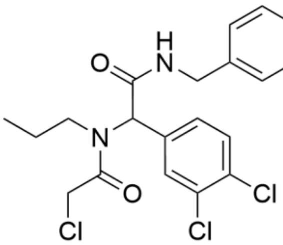

A round bottom flask was prepared with molecular sieve (100 mg; 4 Å) and dry MeOH (4 mL). n-Propylamine (157 µL, 1.89 mmol, 1.20 eq) and 3,4-dichlorobenzaldehyde (340 mg, 1.89 mmol, 1.20 eq) were added and stirred for 30 min at room temperature, before 2-chloroacetic acid (150 mg, 1.57 mmol, 1.00 eq) was added to the mixture. After additional 10 min, benzyl isocyanide (220 µL, 1.57 mmol, 1.00 eq) was added and the resulting solution was stirred for 72 h at room temperature. The

resulting precipitate was filtered, washed with MeOH (20 mL), dissolved in CH_2Cl_2 (20 mL) to remove the molecular sieve. The solvent was removed under reduced pressure and the resulting solid was dried *in vacuo*. DCAF11 ligand **6** was afforded as a with powder (358 mg, 0,837 mmol). Yield 53%; mp. 175 – 177 °C; Rf = 0.39 (cyclohexane/EtOAc, 2/1, *v*/*v*); **^{1}H NMR** (500 MHz, DMSO-d_6) δ = 8.55 (t, *J* = 5.9 Hz, 1H), 7.64 (d, *J* = 8.4 Hz, 1H), 7.53 (s, 1H), 7.37 – 7.19 (m, 6H), 5.81 (s, 1H), 4.56 – 4.23 (m, 4H), 3.30 – 3.24 (m, 2H), 1.52 – 0.79 (m, 2H), 0.68 – 0.55 (m, 3H); **^{13}C NMR** (126 MHz, DMSO-d_6) δ = 167.9, 166.7, 139.0, 137.4, 131.1, 130.8, 130.6, 130.4, 129.6, 128.2, 127.2, 126.8, 61.2, 48.2, 42.5, 42.4, 22.5, 10.8. **HRMS (ESI)** *m/z* $[M+Na]^+$ calcd for $C_{20}H_{21}Cl_3N_2O_2$ 449.0561, found 449.0537; **HPLC** (gradient A), t_R = 13.57 min, 99.0% purity.

3.4. NMR Data of compounds in biological testing

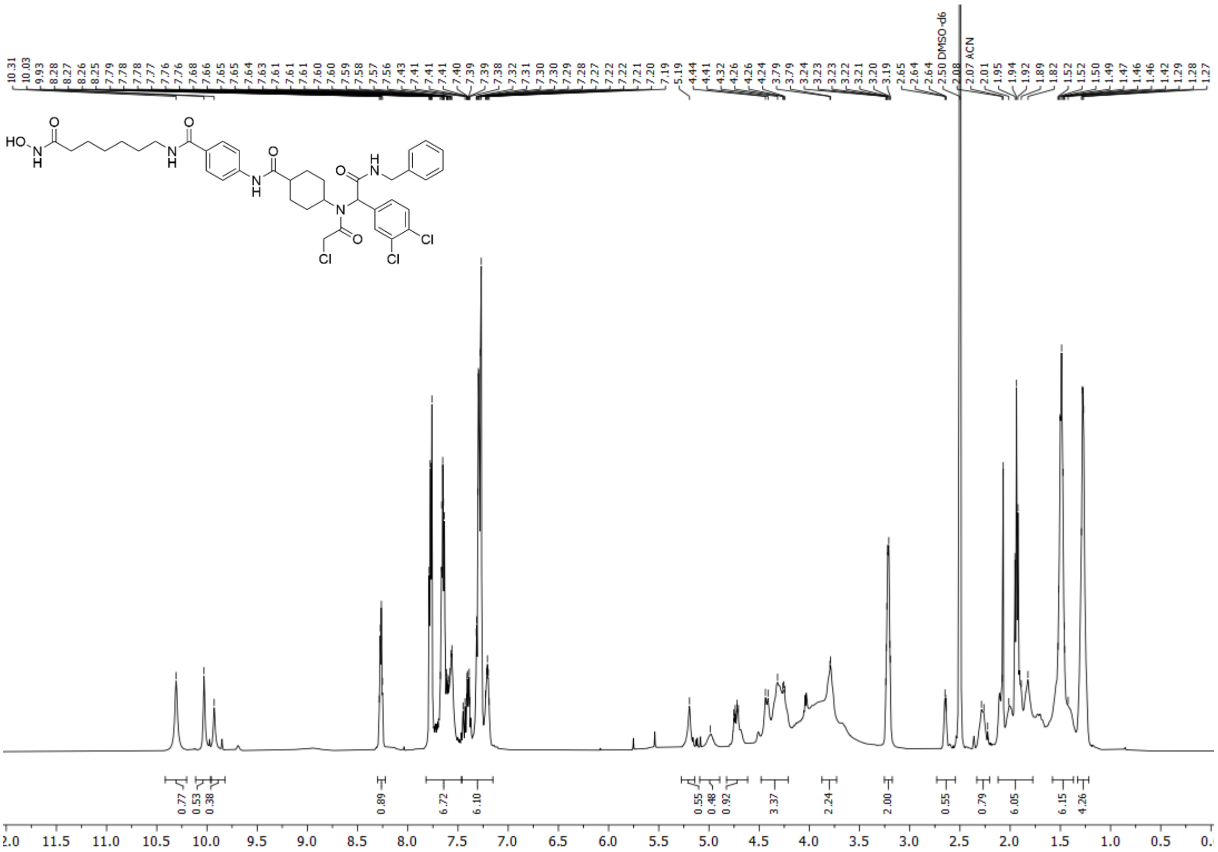

^{1}**H NMR** spectrum of **1a** (500 MHz, DMSO-d_6).

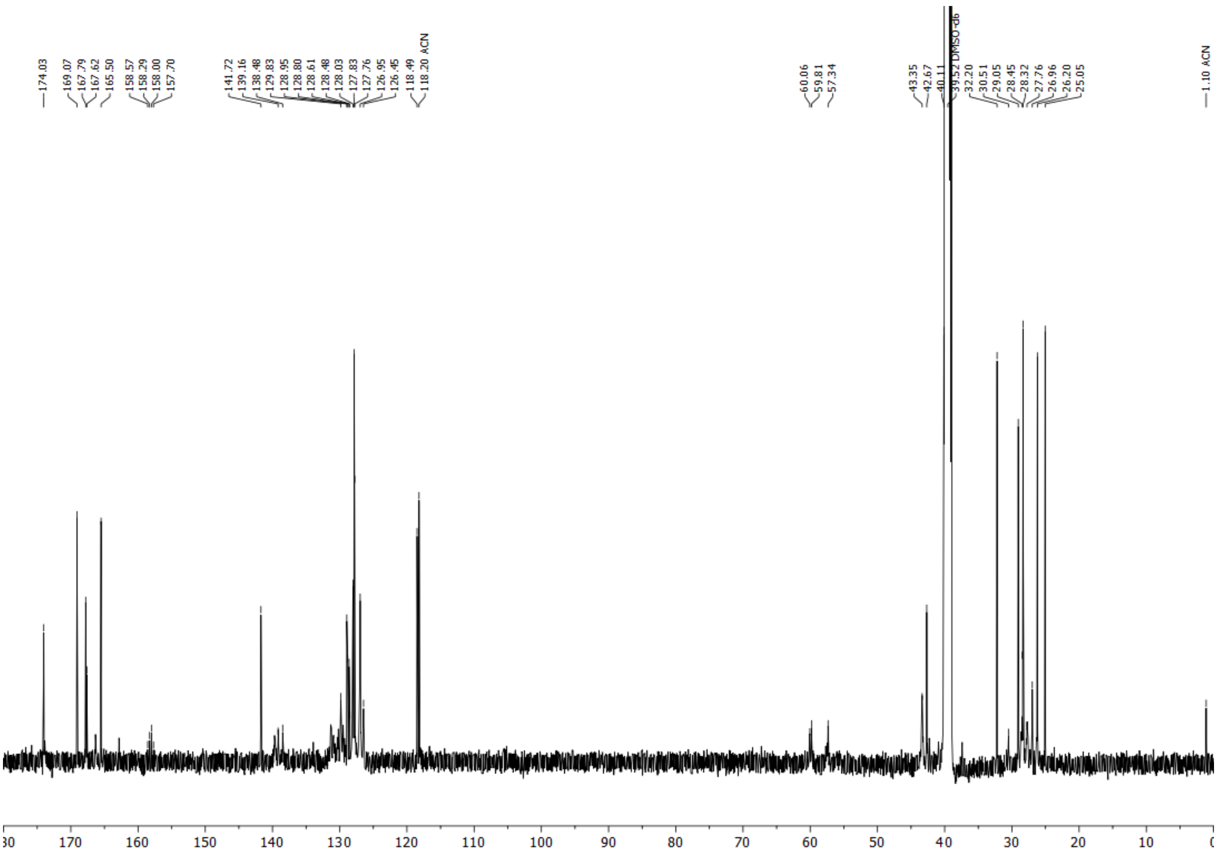

^{13}**C NMR** spectrum of **1a** (126 MHz, DMSO-d_6).

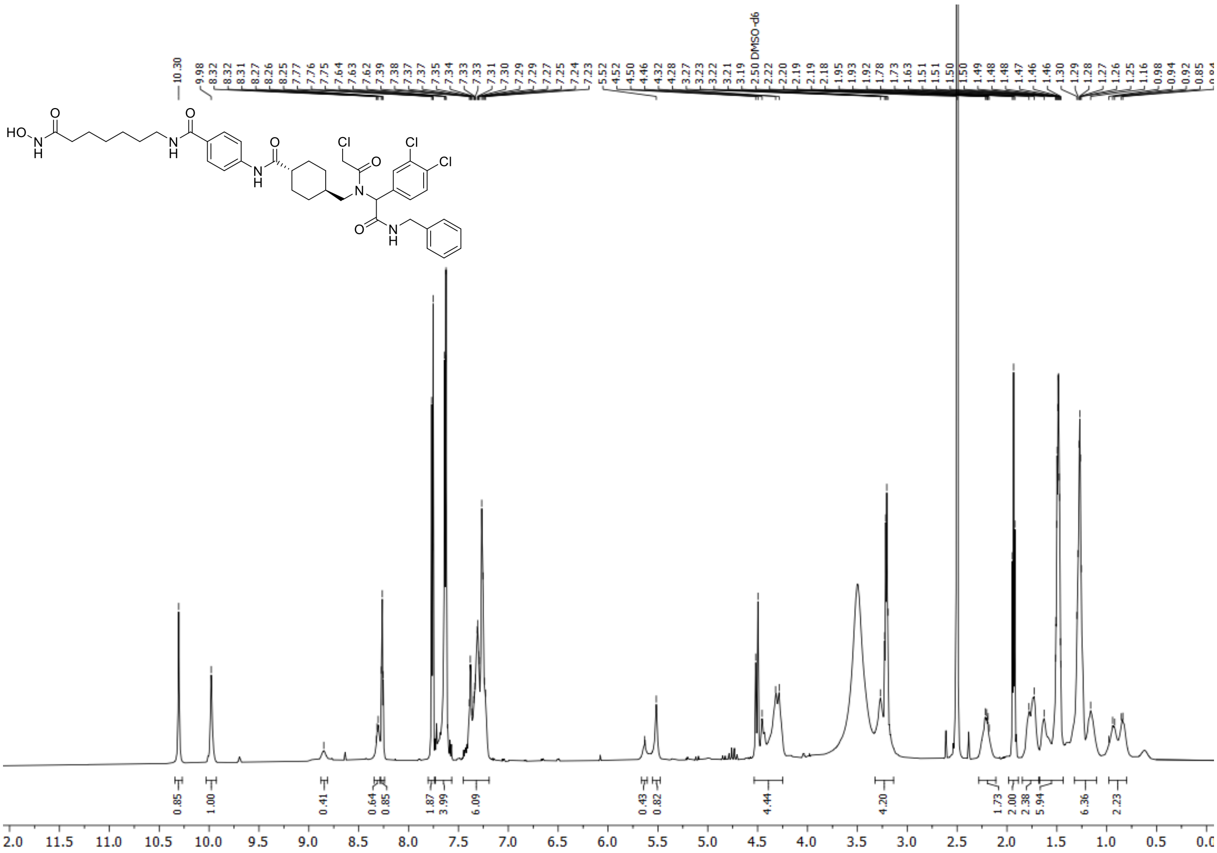

^{1}H NMR spectrum of **1b** (600 MHz, DMSO-d_6).

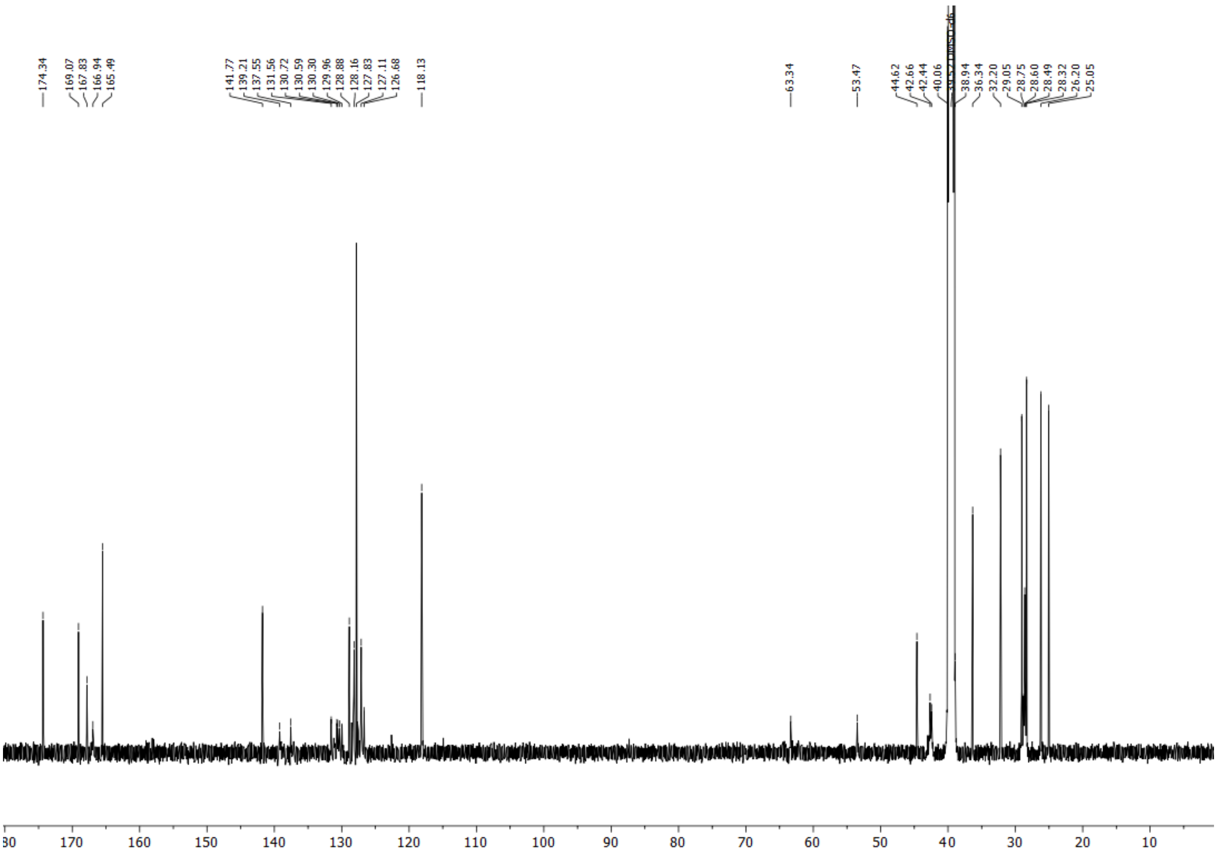

^{13}C NMR spectrum of **1b** (151 MHz, DMSO-d_6).

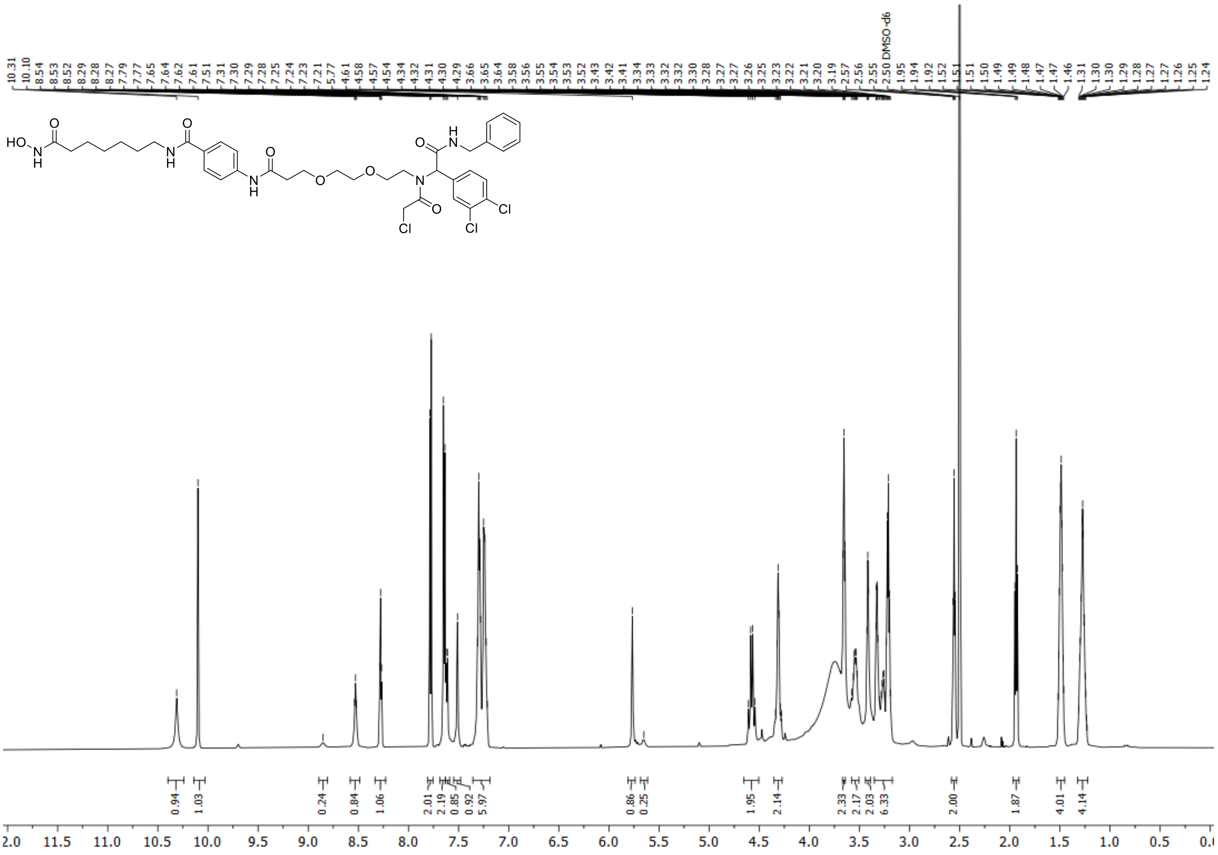

^{1}**H NMR** spectrum of **1c** (600 MHz, DMSO-d_6).

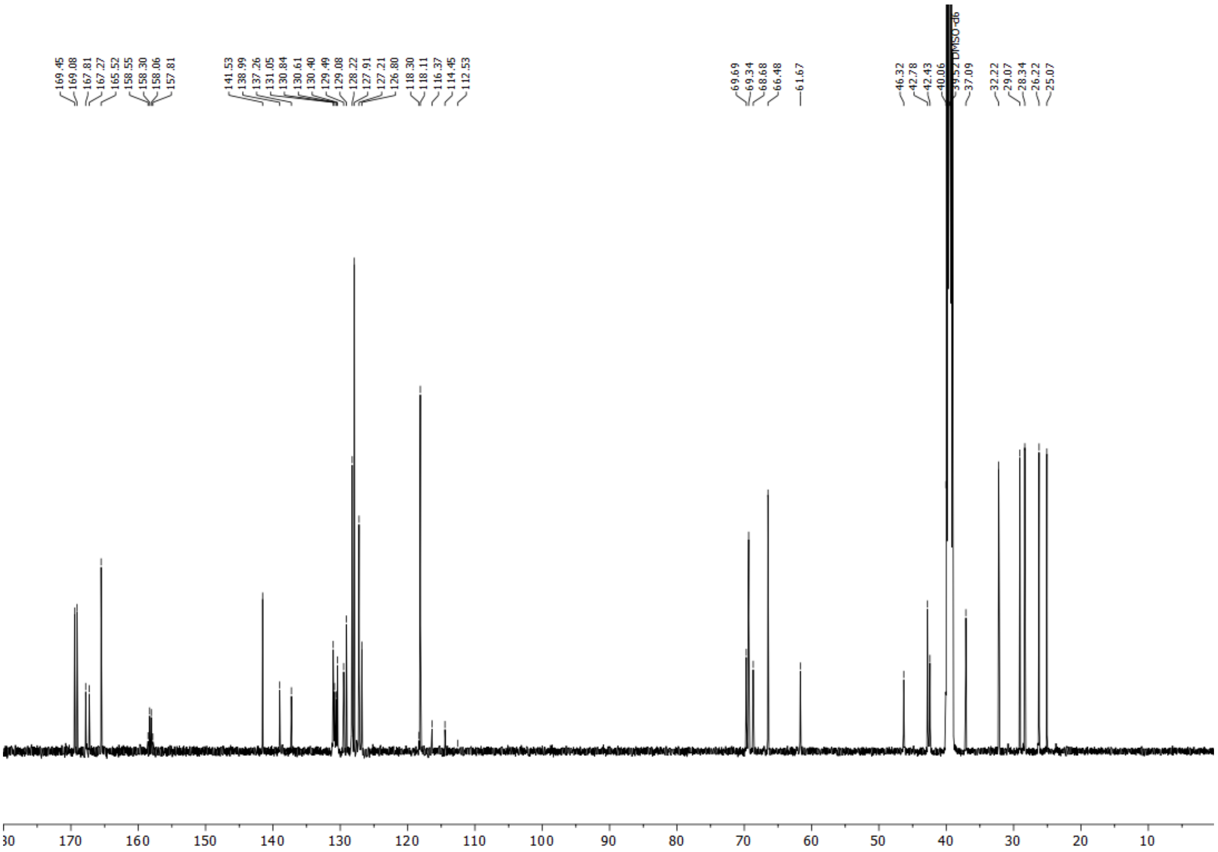

^{13}**C NMR** spectrum of **1c** (151 MHz, DMSO-d_6).

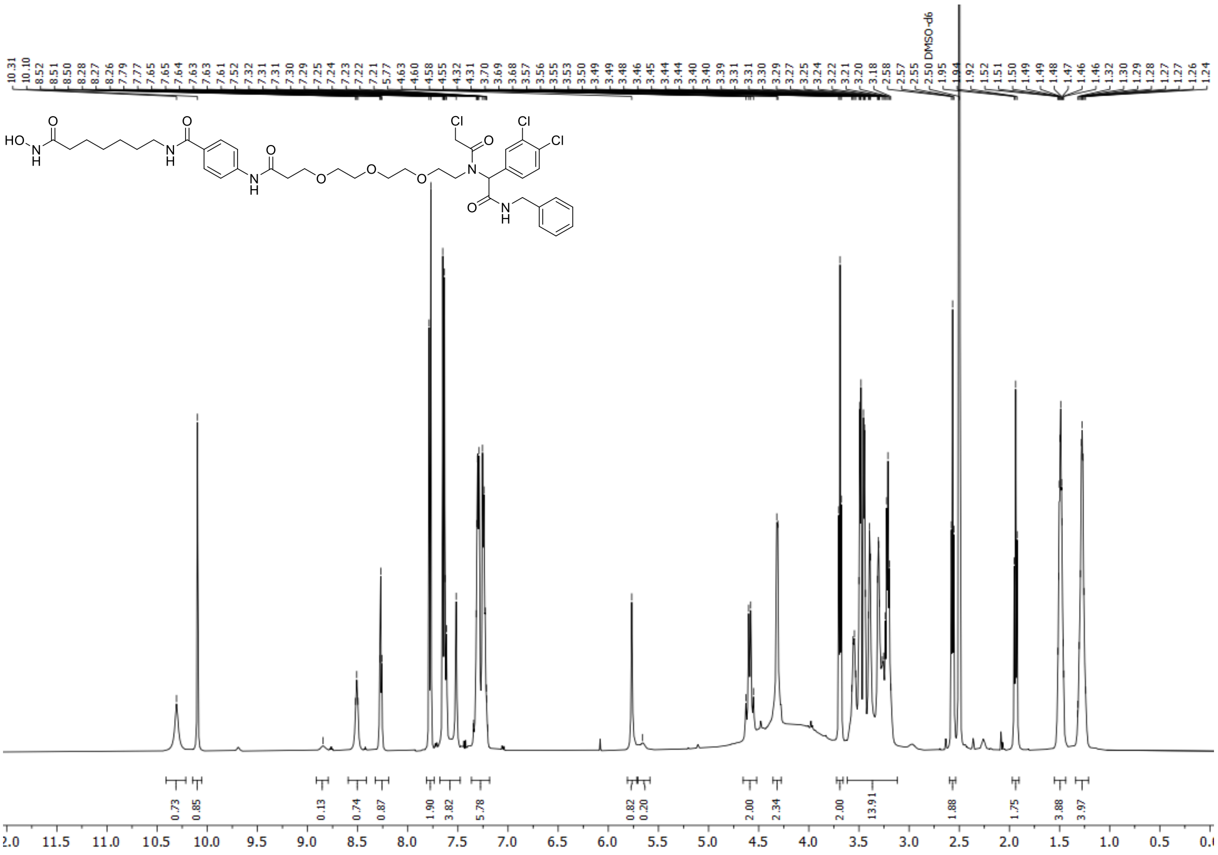

^{1}H NMR spectrum of **1d** (500 MHz, DMSO-d_6).

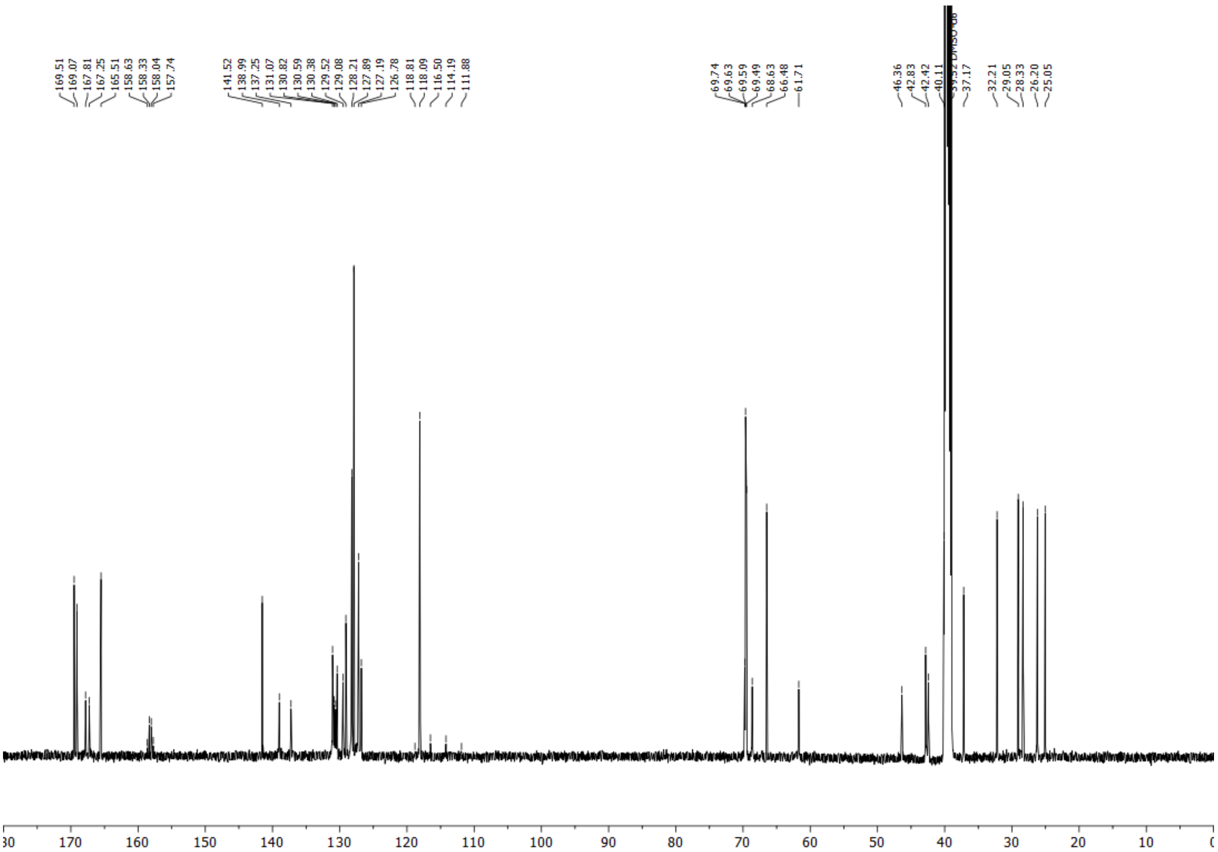

^{13}C NMR spectrum of **1d** (126 MHz, DMSO-d_6).

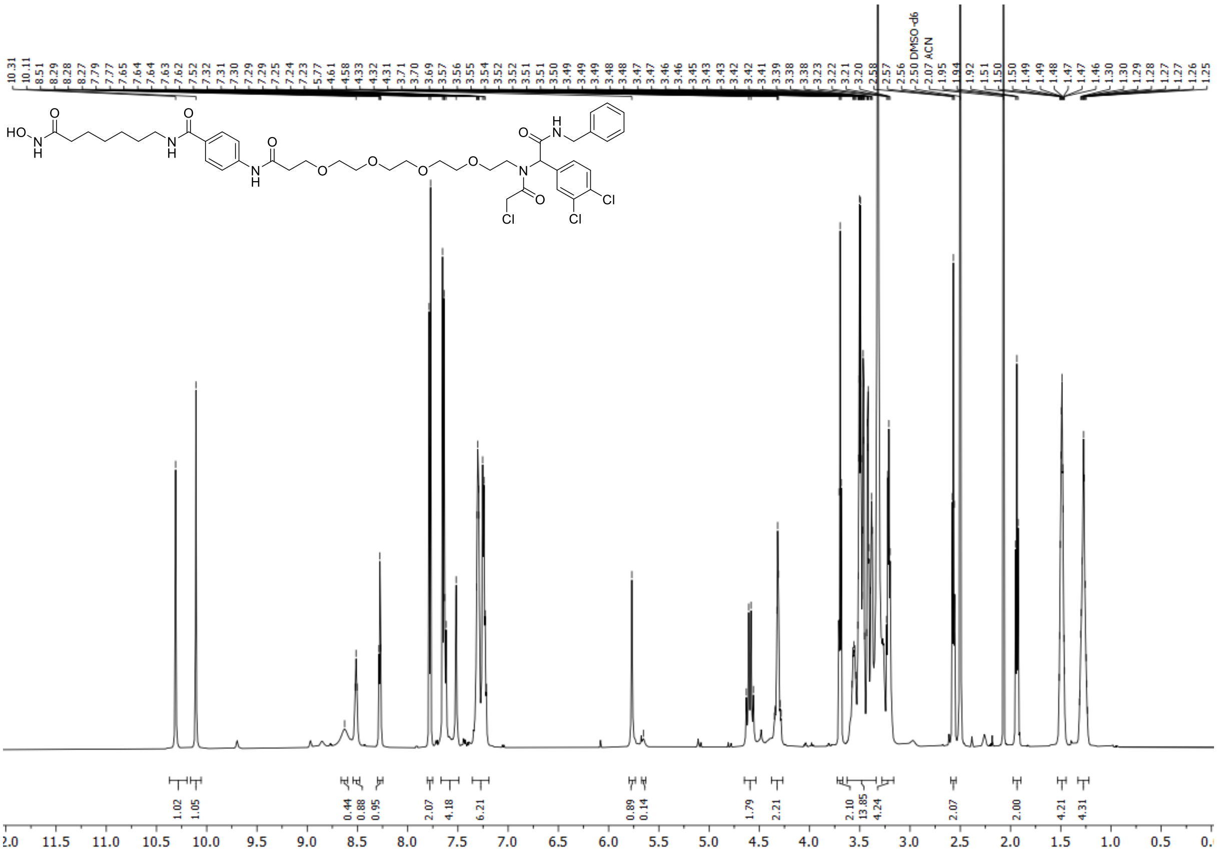

^{1}H NMR spectrum of **1e** (600 MHz, DMSO-d_6).

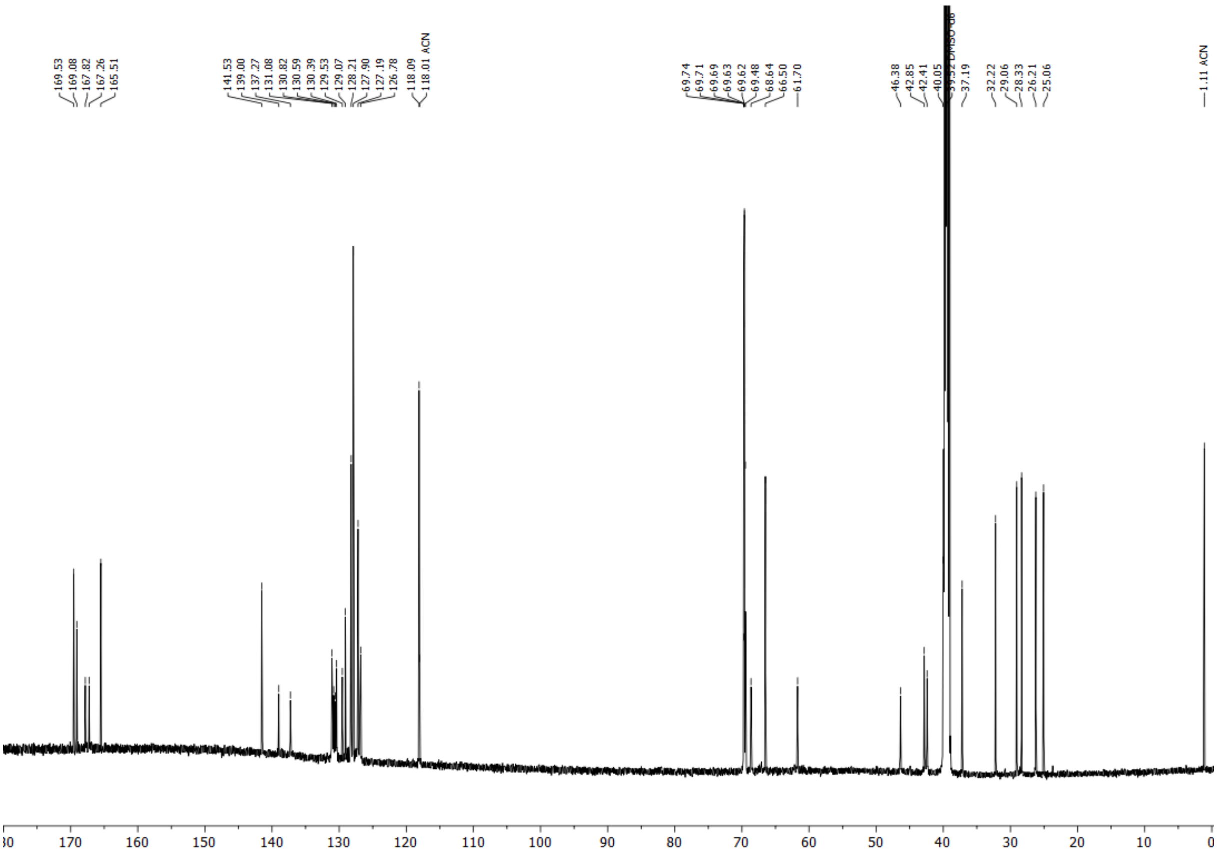

^{13}C NMR spectrum of **1e** (151 MHz, DMSO-d_6).

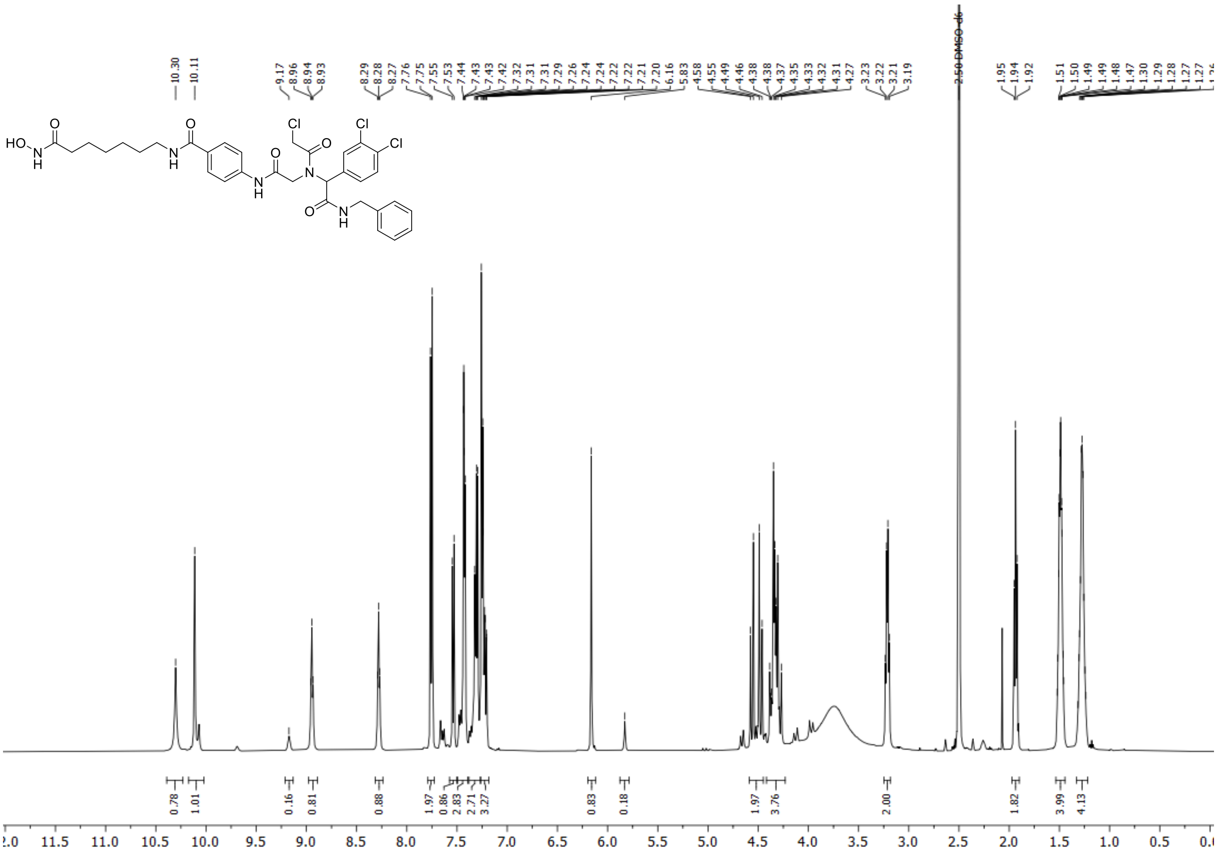

¹H NMR spectrum of **1f** (500 MHz, DMSO-d_6).

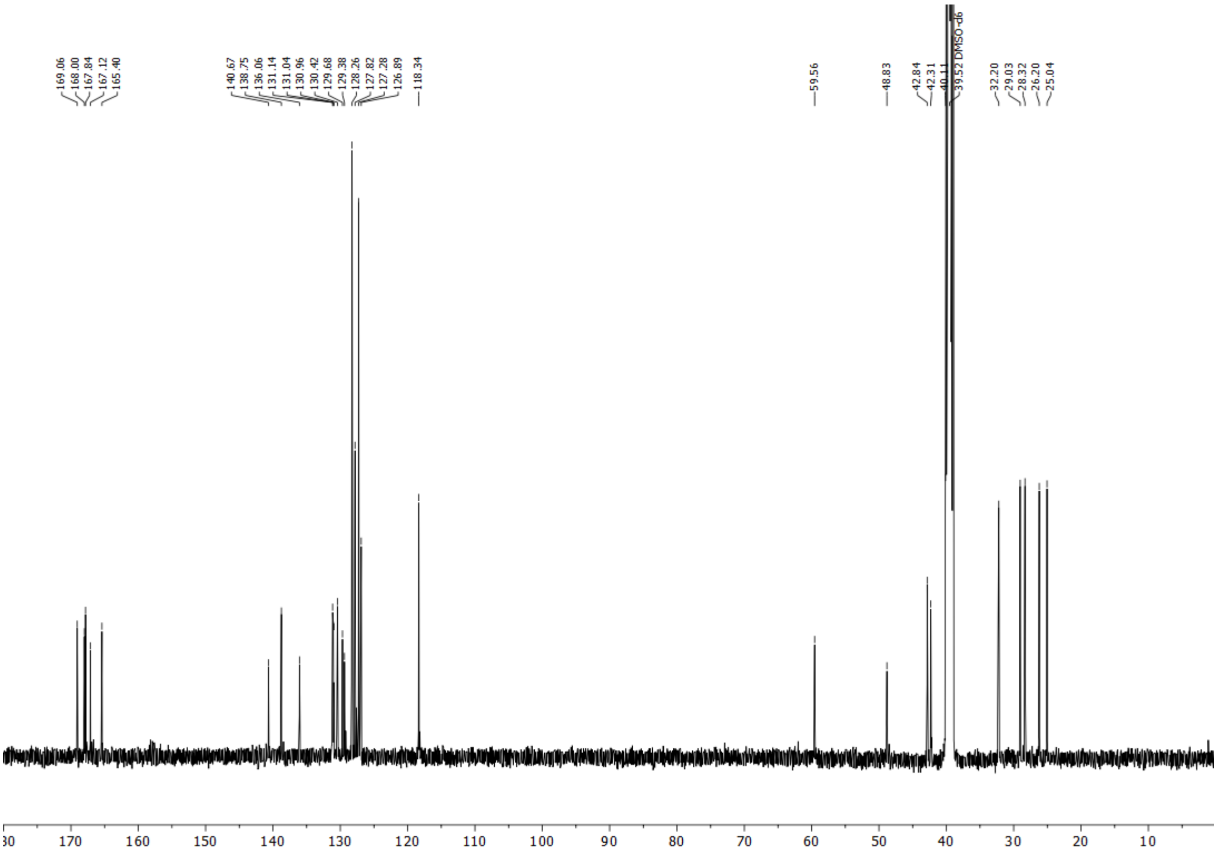

¹³C NMR spectrum of **1f** (126 MHz, DMSO-d_6).

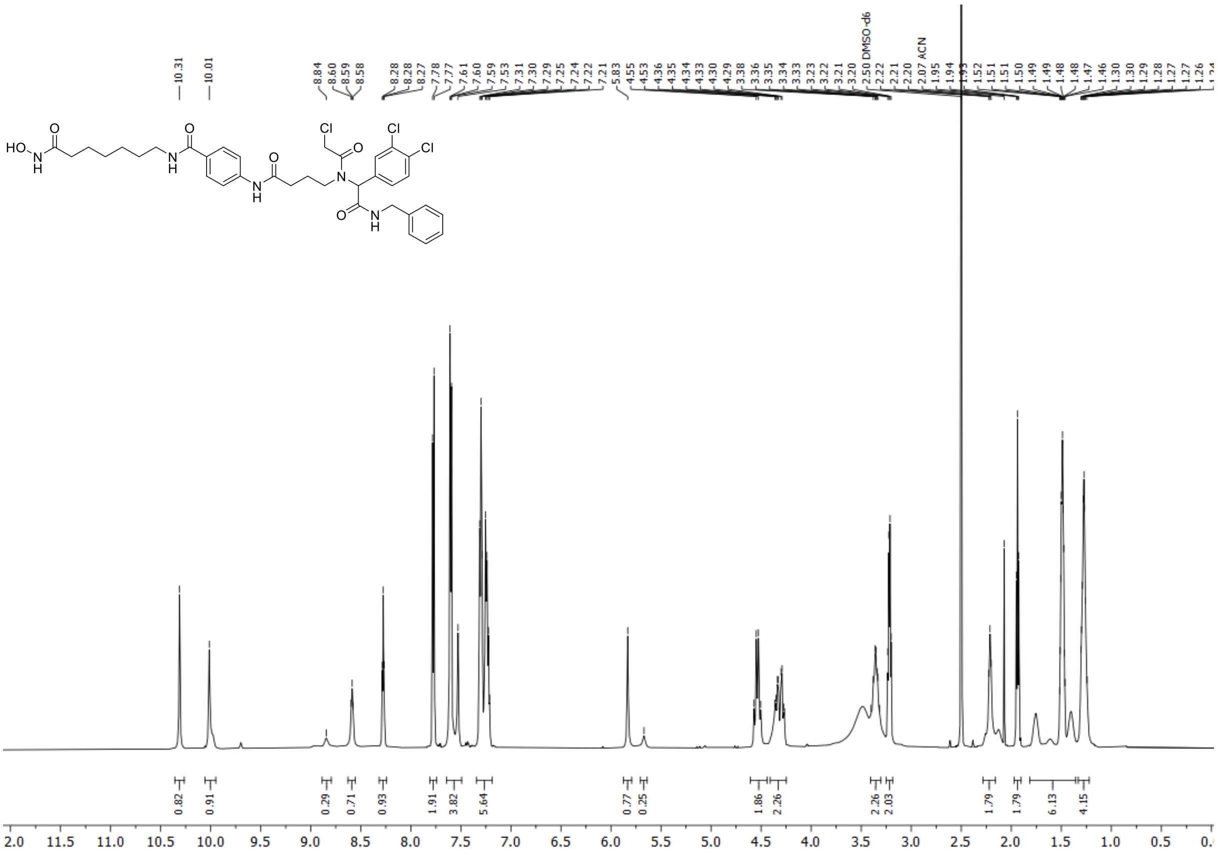

^{1}H NMR spectrum of **1g** (600 MHz, DMSO-d_6).

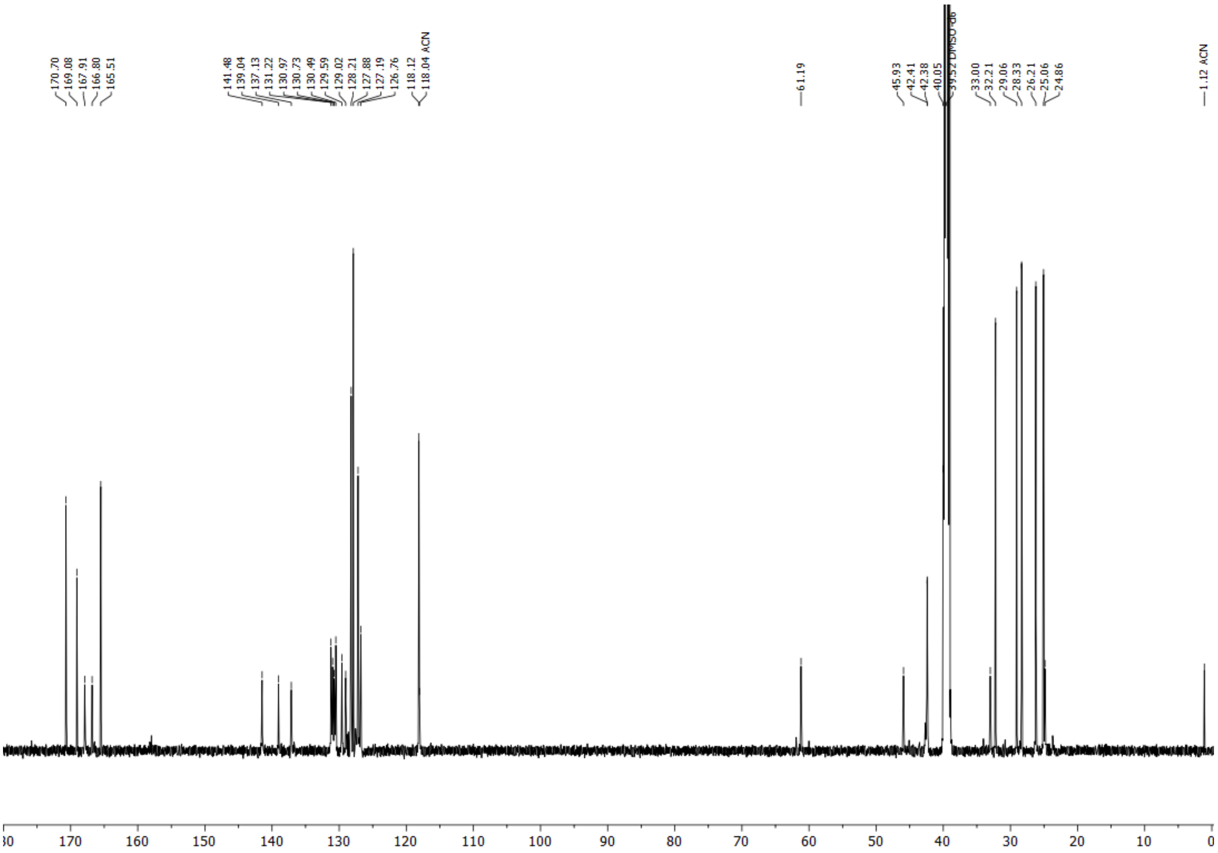

^{13}C NMR spectrum of **1g** (151 MHz, DMSO-d_6).

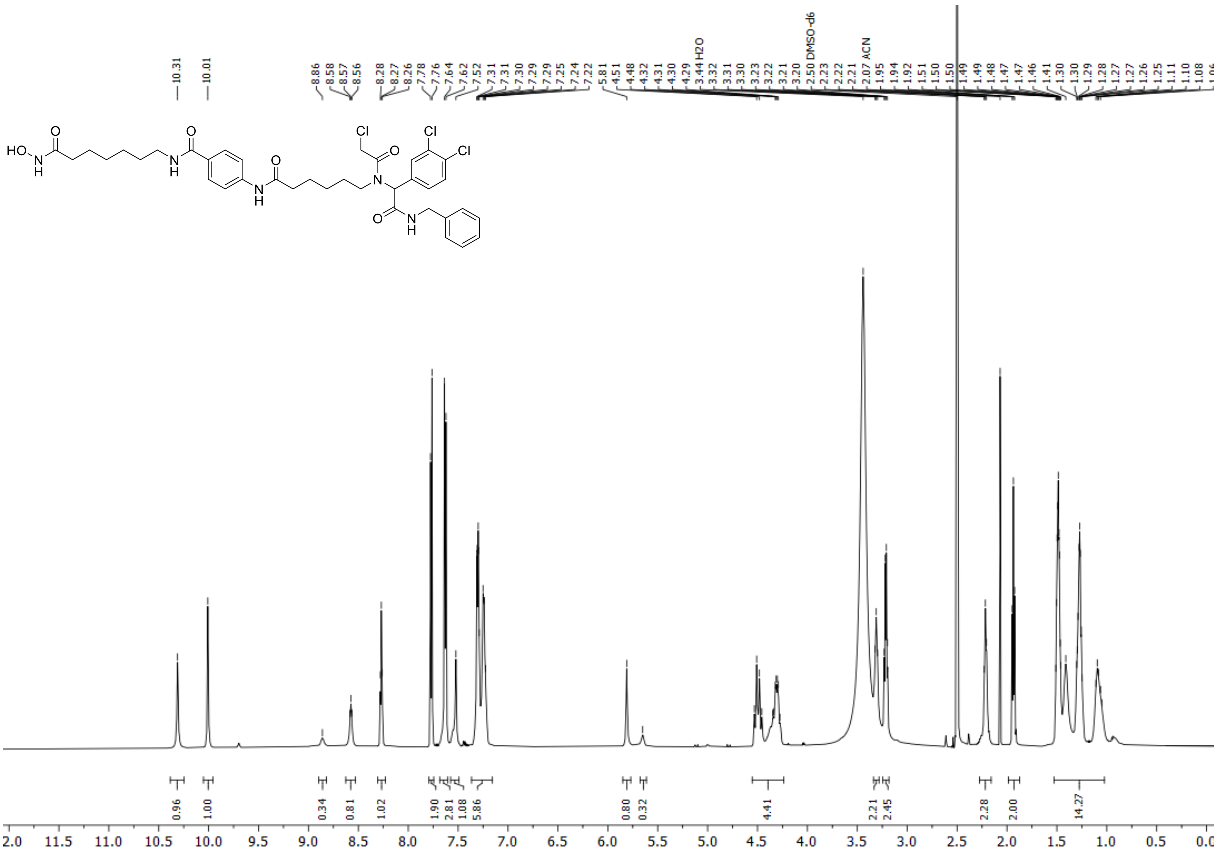

^{1}H NMR spectrum of **1h** (600 MHz, DMSO-d_6).

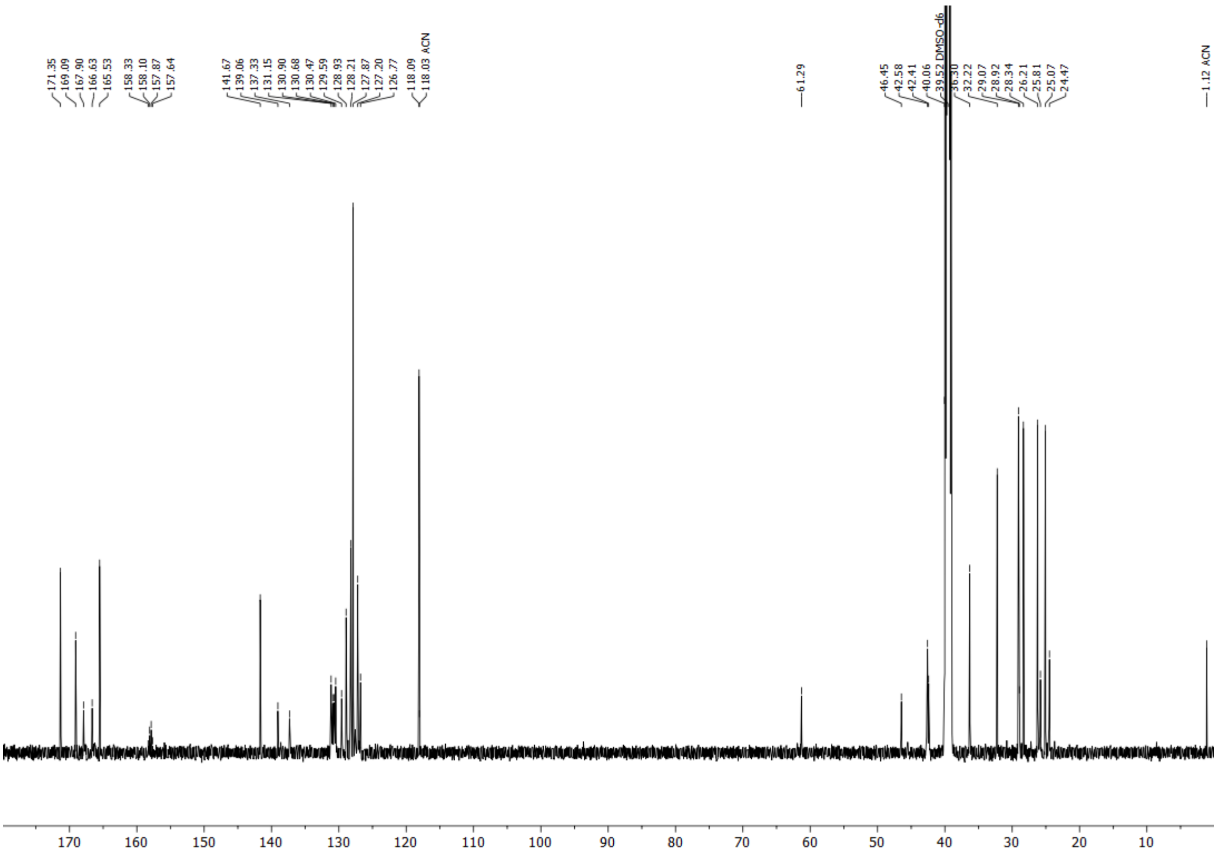

^{13}C NMR spectrum of **1h** (151 MHz, DMSO-d_6).

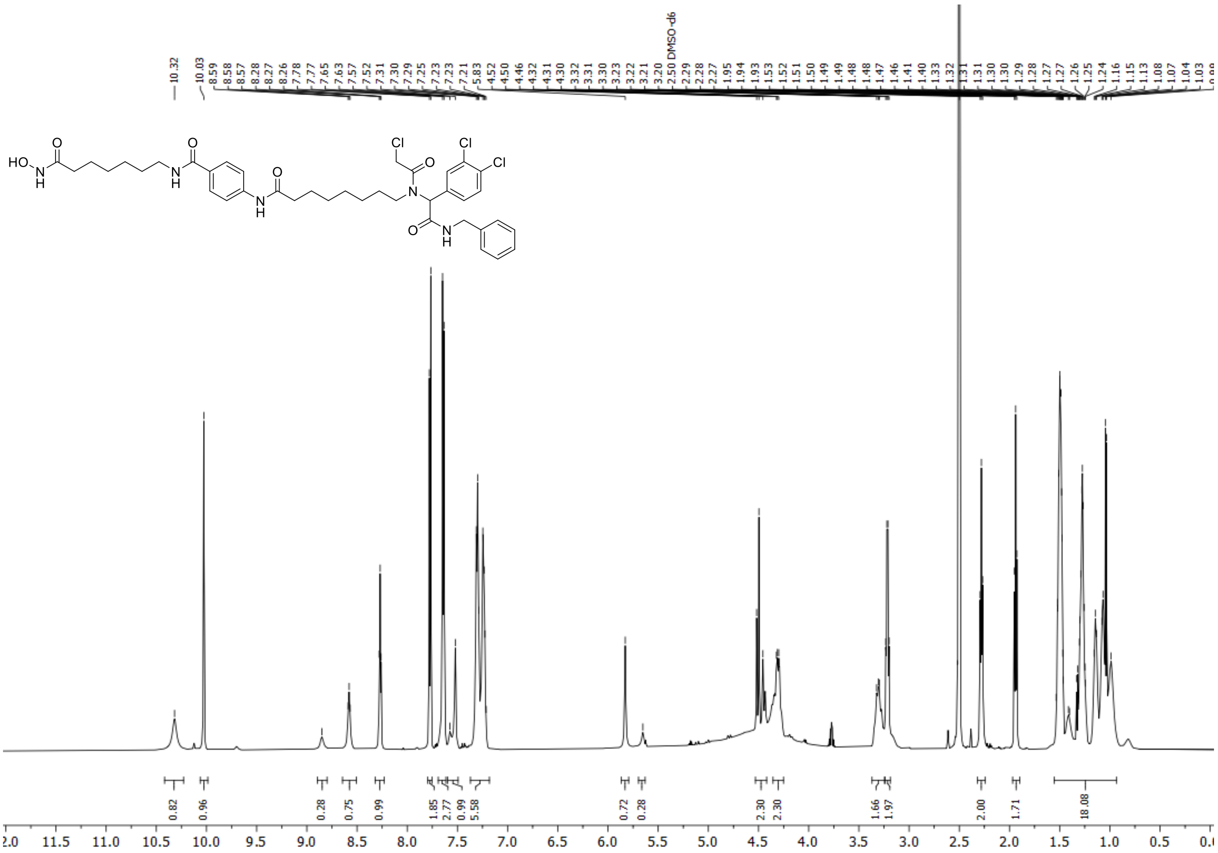

^{1}H NMR spectrum of **1i** (600 MHz, DMSO-d_6).

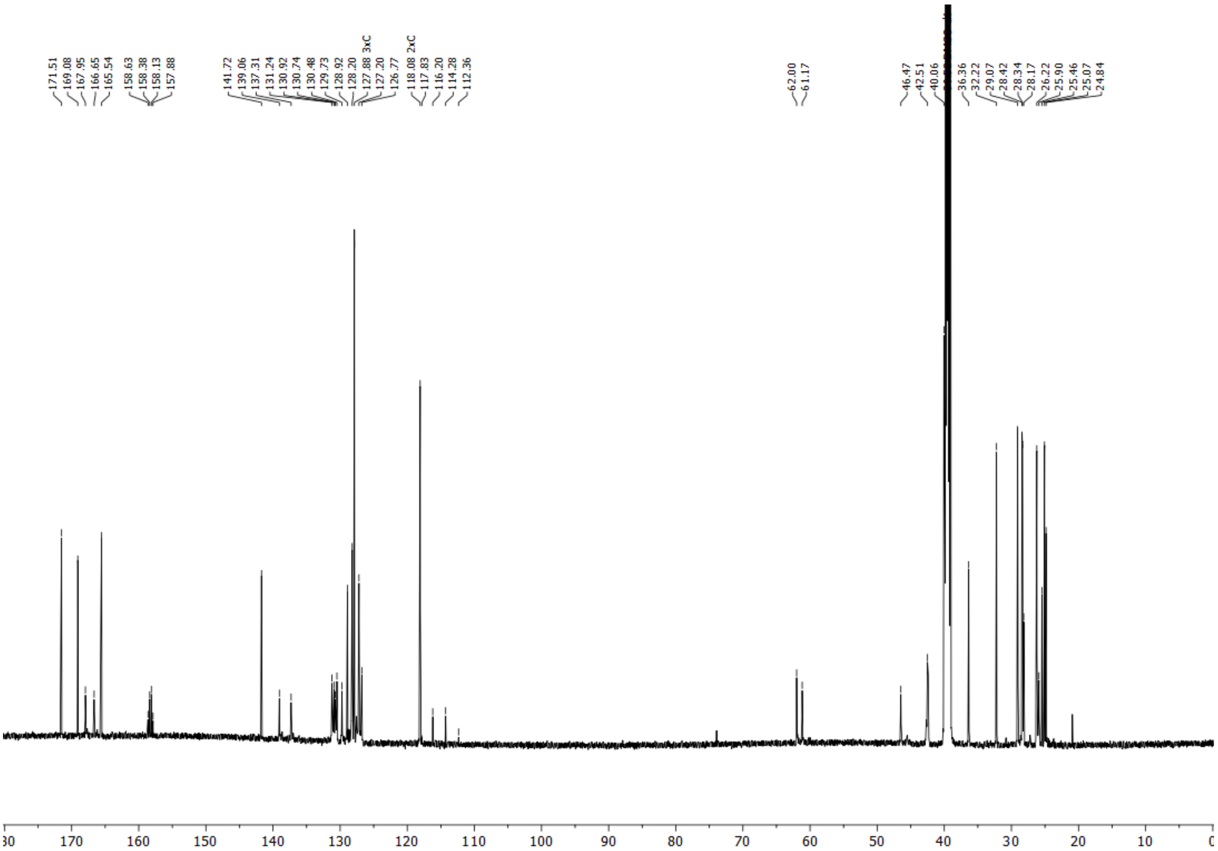

^{13}C NMR spectrum of **1i** (151 MHz, DMSO-d_6).

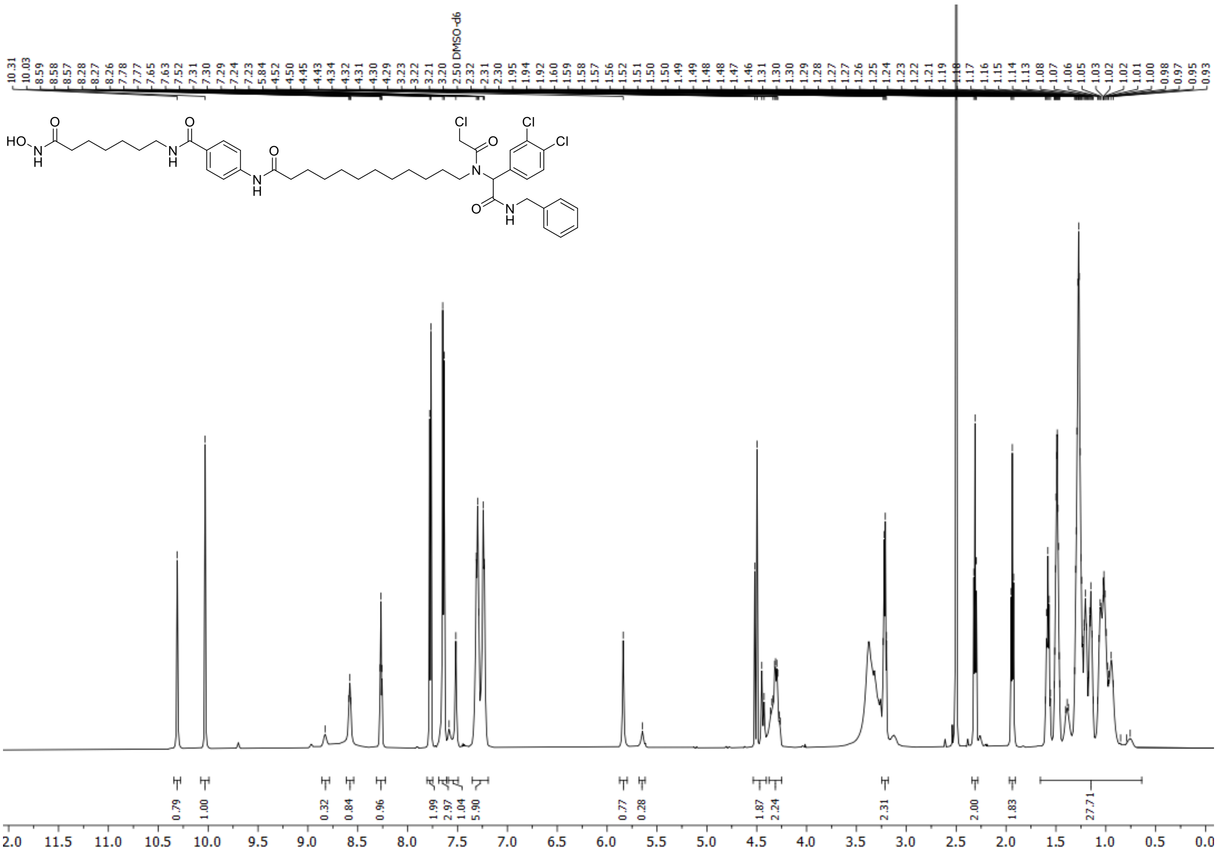

^{1}H NMR spectrum of **1j** (600 MHz, DMSO-*d*6).

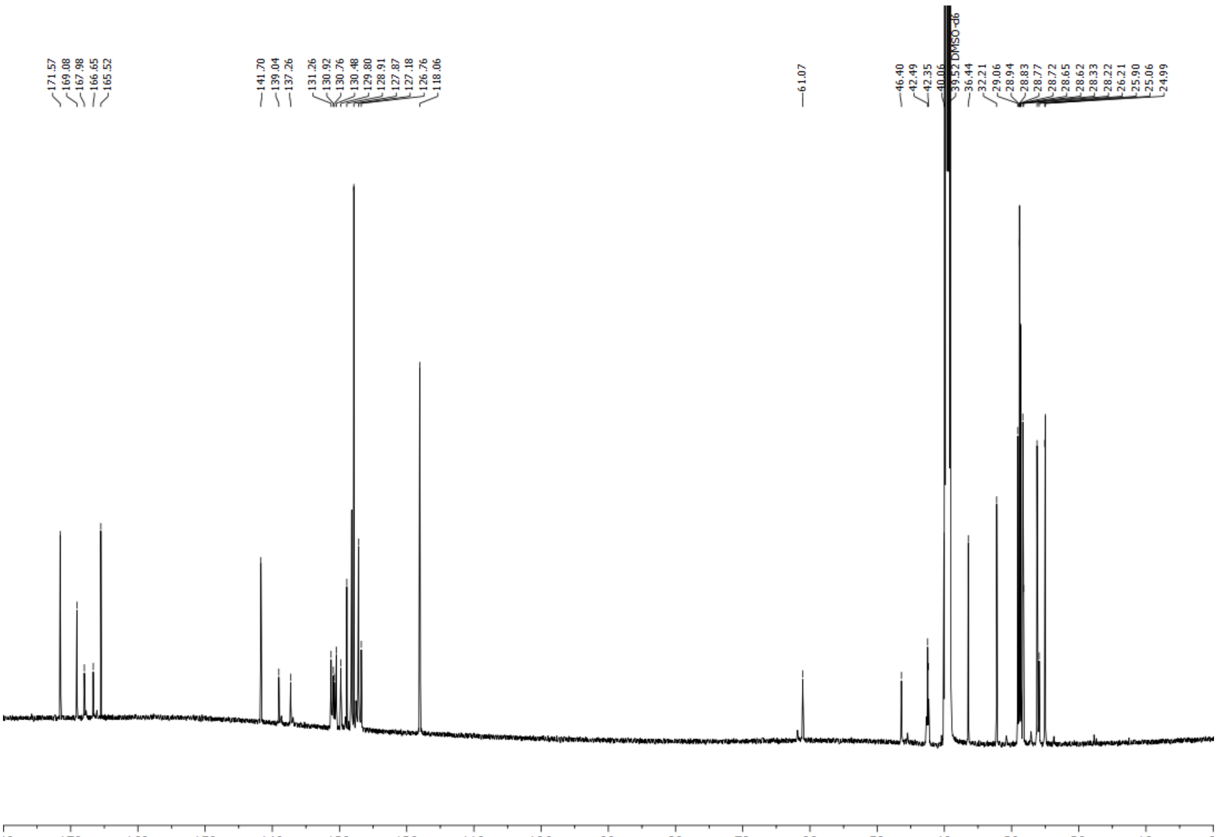

^{13}C NMR spectrum of **1j** (151 MHz, DMSO-*d*6).

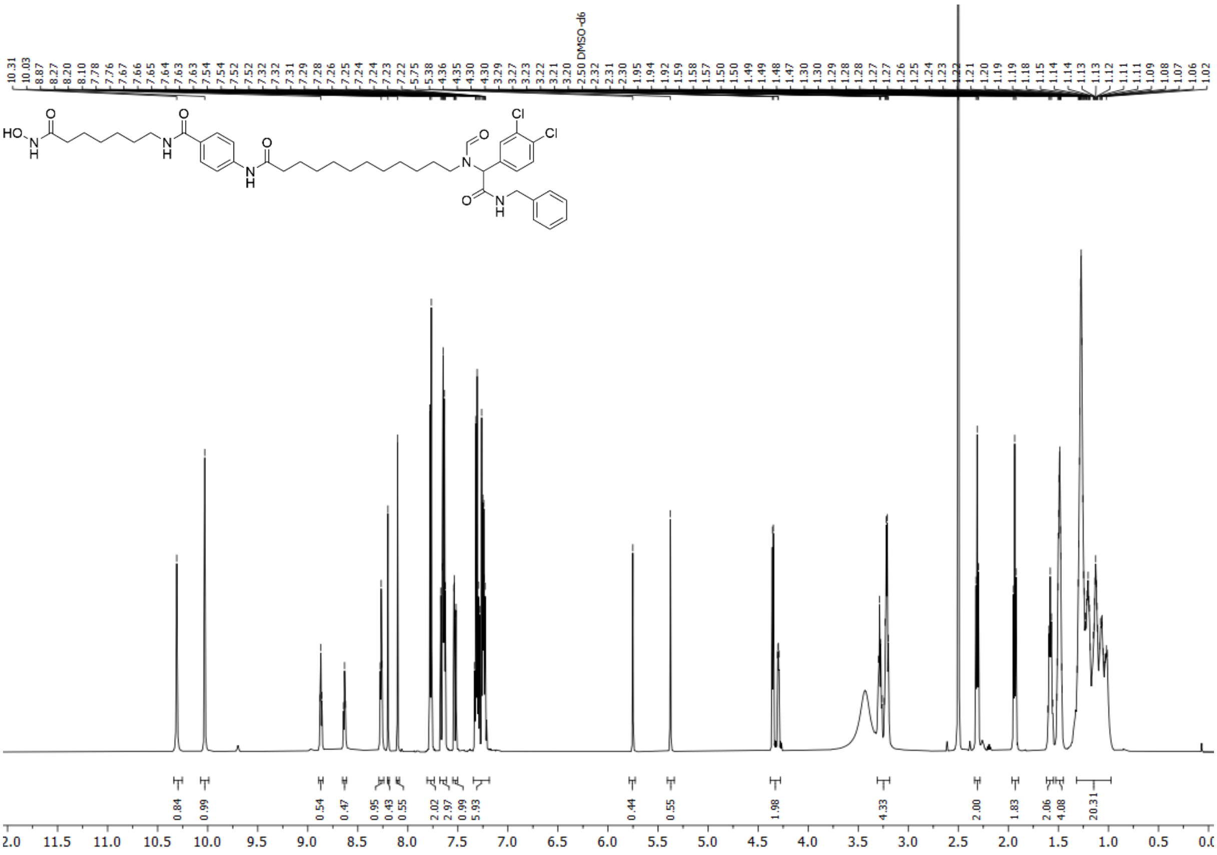

^{1}H NMR spectrum of **1j-nc** (600 MHz, DMSO-d_6).

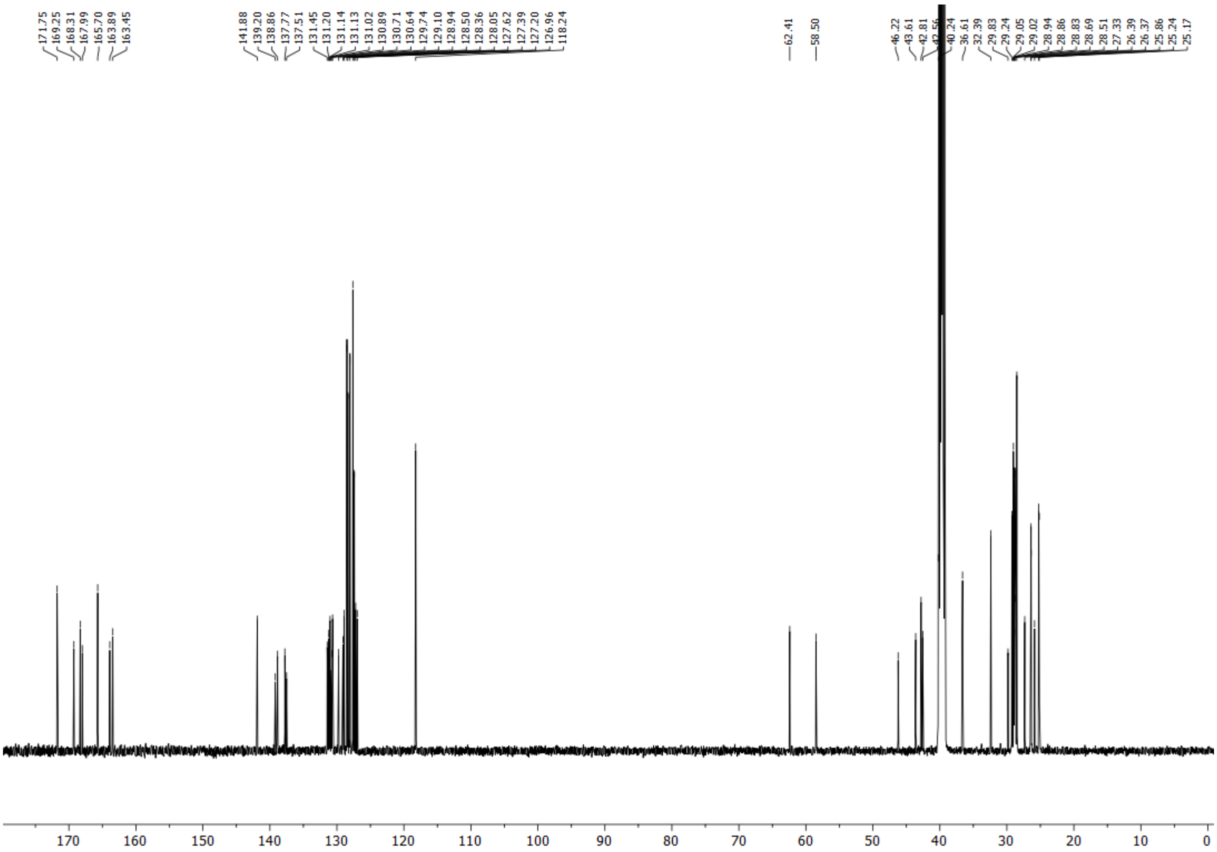

^{13}C NMR spectrum of **1j-nc** (151 MHz, DMSO-d_6).

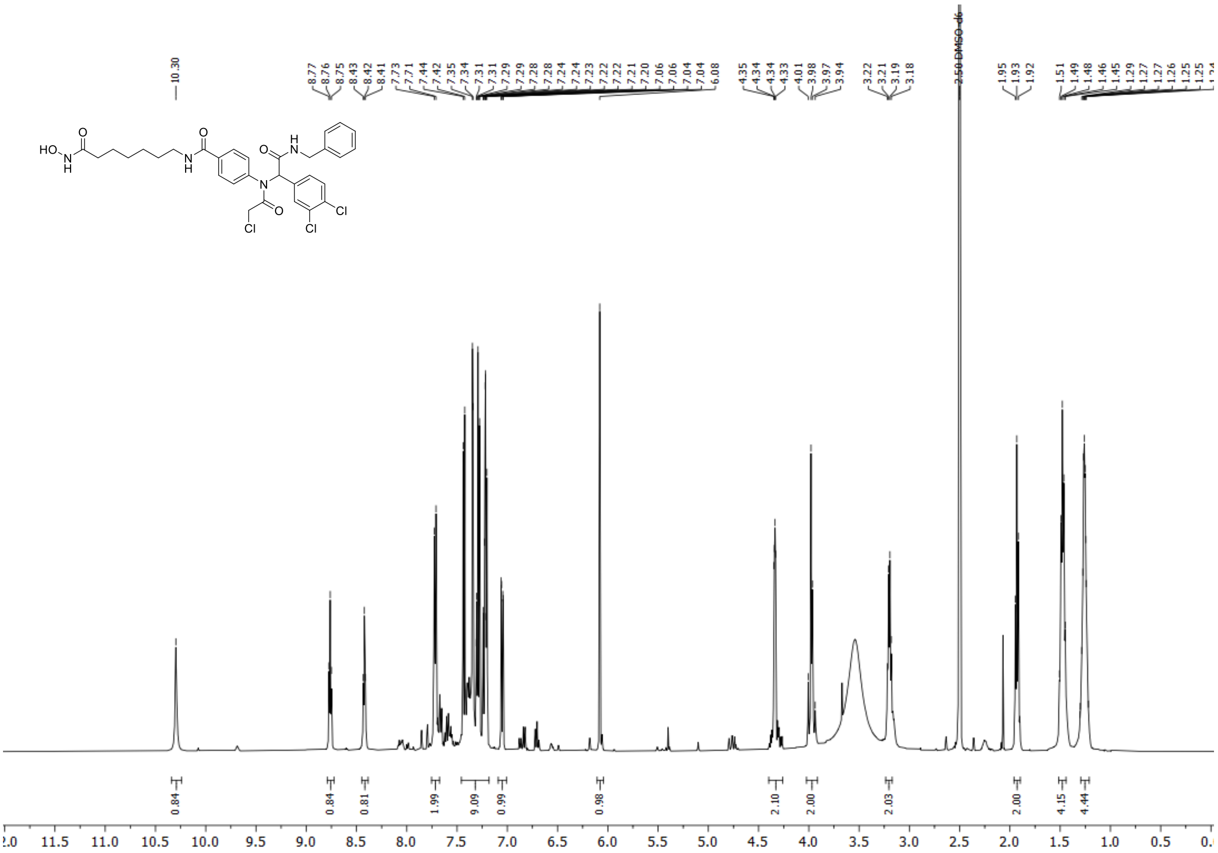

^{1}H NMR spectrum of **2** (500 MHz, DMSO-d_6).

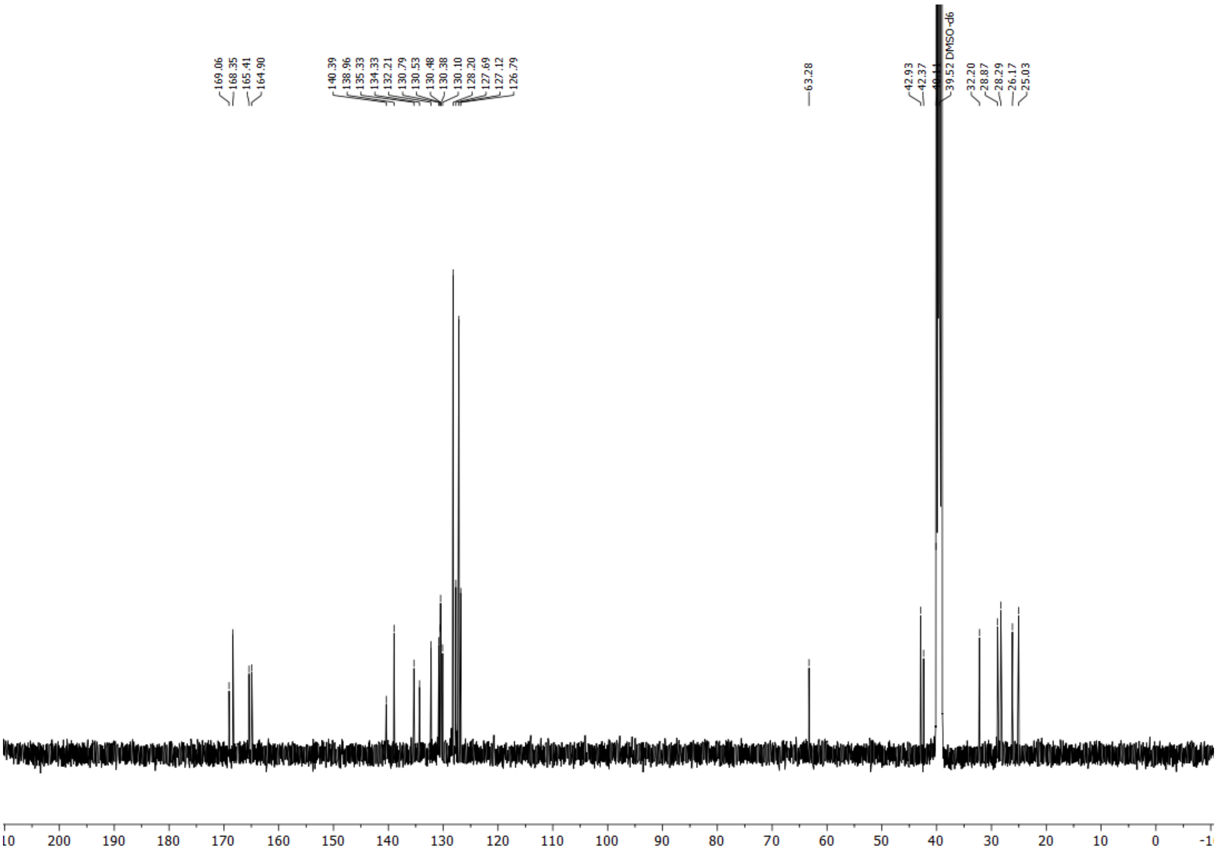

^{13}C NMR spectrum of **2** (126 MHz, DMSO-d_6).

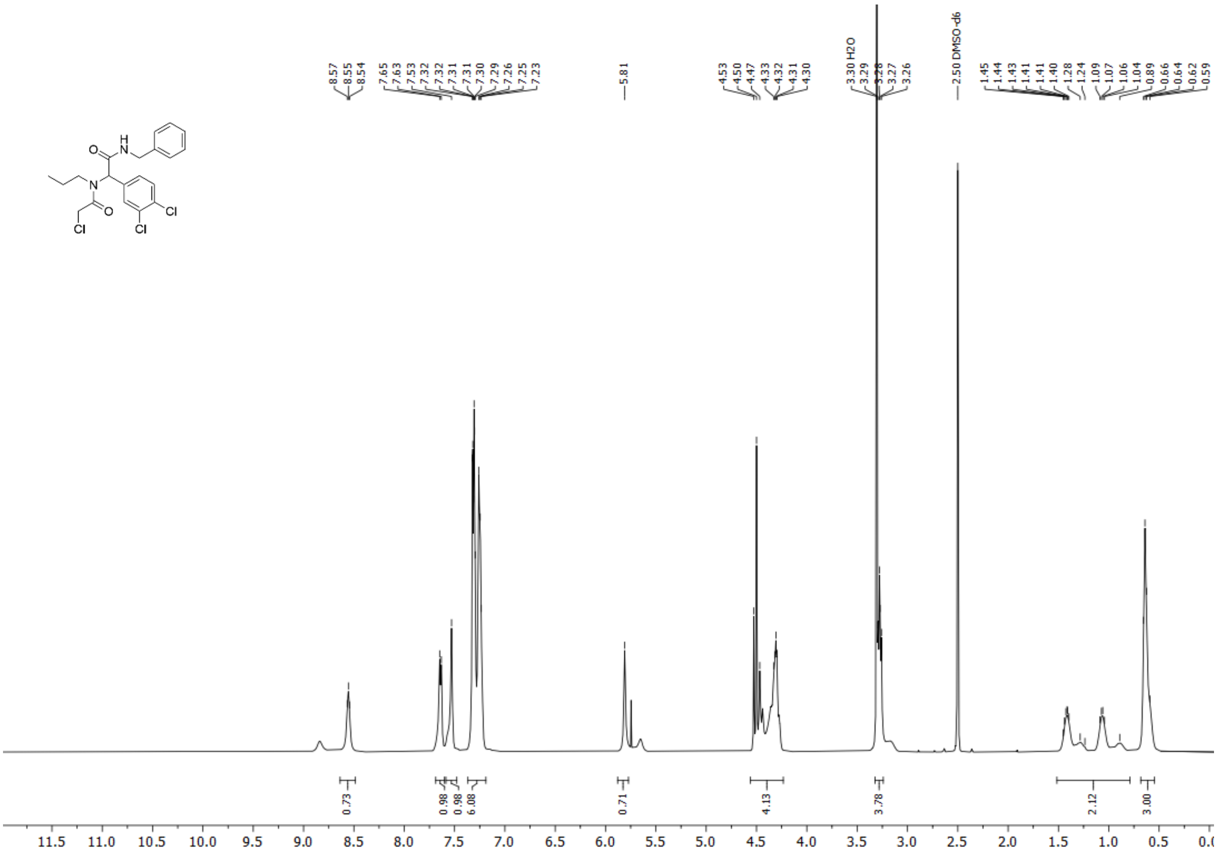

^{1}H NMR spectrum of **6** (500 MHz, DMSO-d_6).

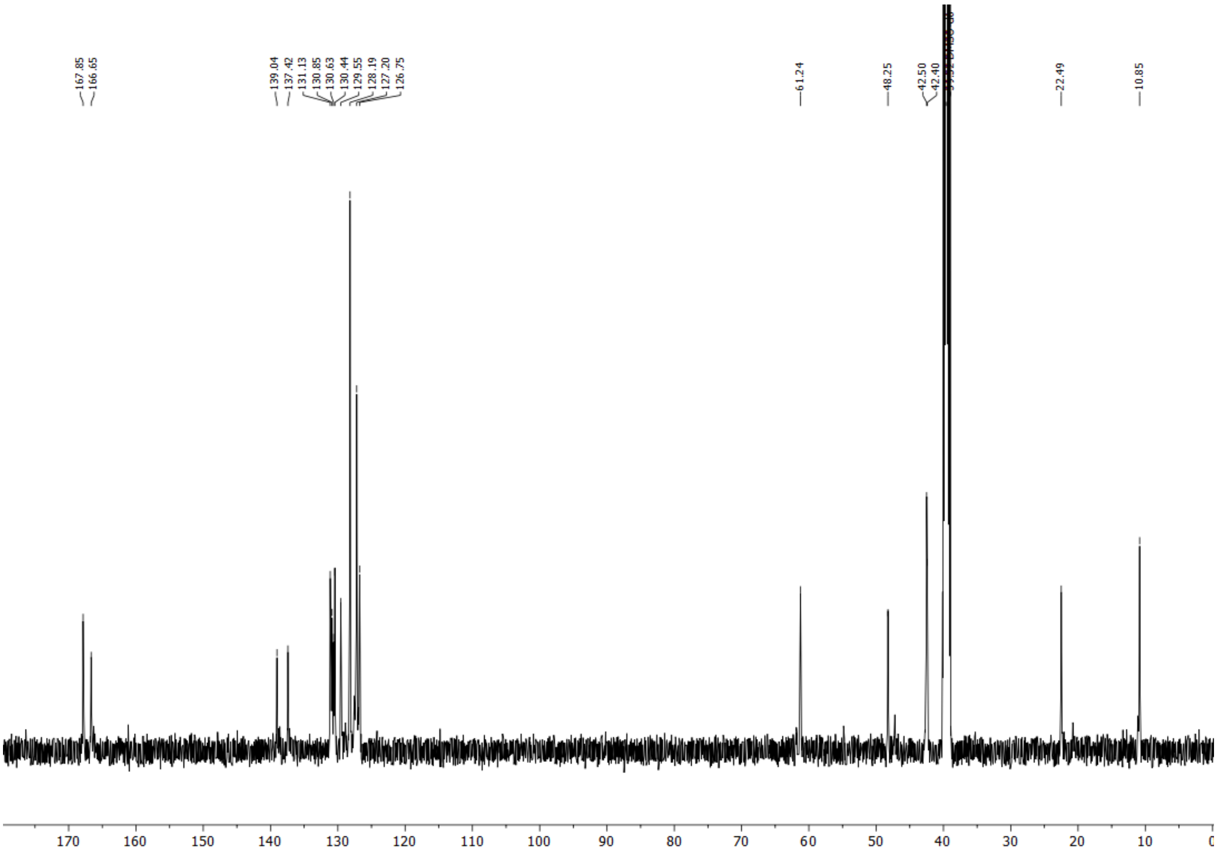

^{13}C NMR spectrum of **6** (126 MHz, DMSO-d_6).

3.5. HPLC chromatograms of target compounds

Chromatogram

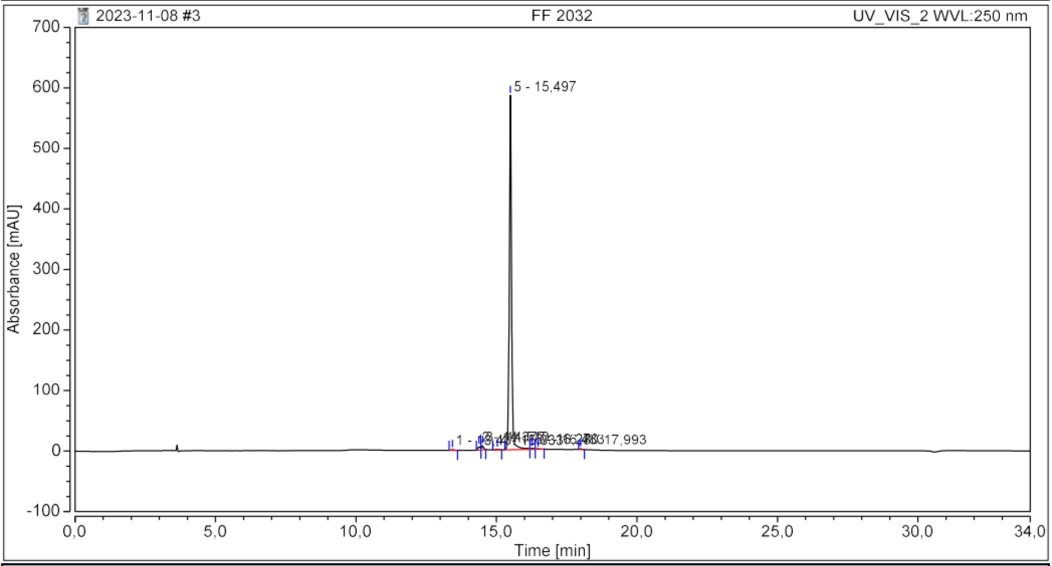

Integration Results

No.	Peak Name	Retention Time min	Area mAU*min	Height mAU	Relative Area %	Relative Height %	Amount n.a.
1		13,437	0,118	1,401	0,22	0,23	n.a.
2		14,377	0,512	6,725	0,94	1,11	n.a.
3		14,510	0,551	6,906	1,01	1,14	n.a.
4		15,033	0,100	1,108	0,18	0,18	n.a.
5		15,497	52,540	585,140	96,74	96,62	n.a.
6		16,270	0,254	1,929	0,47	0,32	n.a.
7		16,483	0,146	1,083	0,27	0,18	n.a.
8		17,993	0,093	1,338	0,17	0,22	n.a.
**Total:**			**54,313**	**605,630**	**100,00**	**100,00**	

HPLC chromatogram of **1a** (gradient B, purity: 96.7%).

Chromatogram

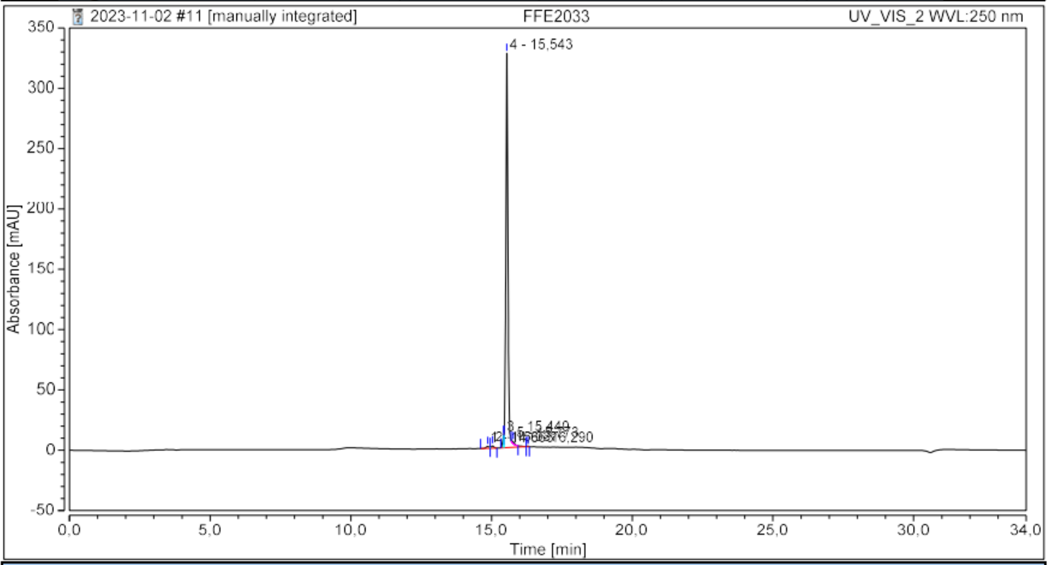

Integration Results

No.	Peak Name	Retention Time min	Area mAU*min	Height mAU	Relative Area %	Relative Height %	Amount n.a.
1		14,863	0,217	1,712	0,72	0,50	n.a.
2		15,037	0,220	2,127	0,74	0,62	n.a.
3		15,440	0,543	10,363	1,81	3,02	n.a.
4		15,543	28,790	326,830	96,04	95,13	n.a.
5		15,773	0,182	2,123	0,61	0,62	n.a.
6		16,290	0,024	0,392	0,08	0,11	n.a.
**Total:**			**29,977**	**343,547**	**100,00**	**100,00**	

HPLC chromatogram of **1b** (gradient B, purity: 96.0%).

Chromatogram

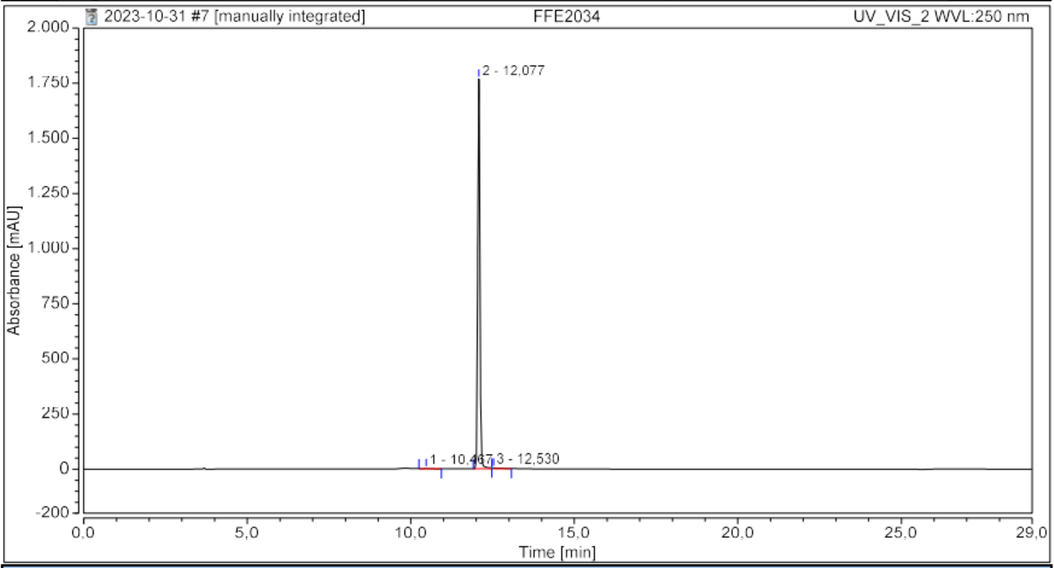

Integration Results

No.	Peak Name	Retention Time min	Area mAU*min	Height mAU	Relative Area %	Relative Height %	Amount n.a.
1		10,467	0,021	0,438	0,02	0,02	n.a.
2		12,077	133,190	1766,184	99,24	99,79	n.a.
3		12,530	0,998	3,251	0,74	0,18	n.a.
**Total:**			**134,209**	**1769,873**	**100,00**	**100,00**	

HPLC chromatogram of **1c** (gradient A, purity: 99.2%).

Chromatogram

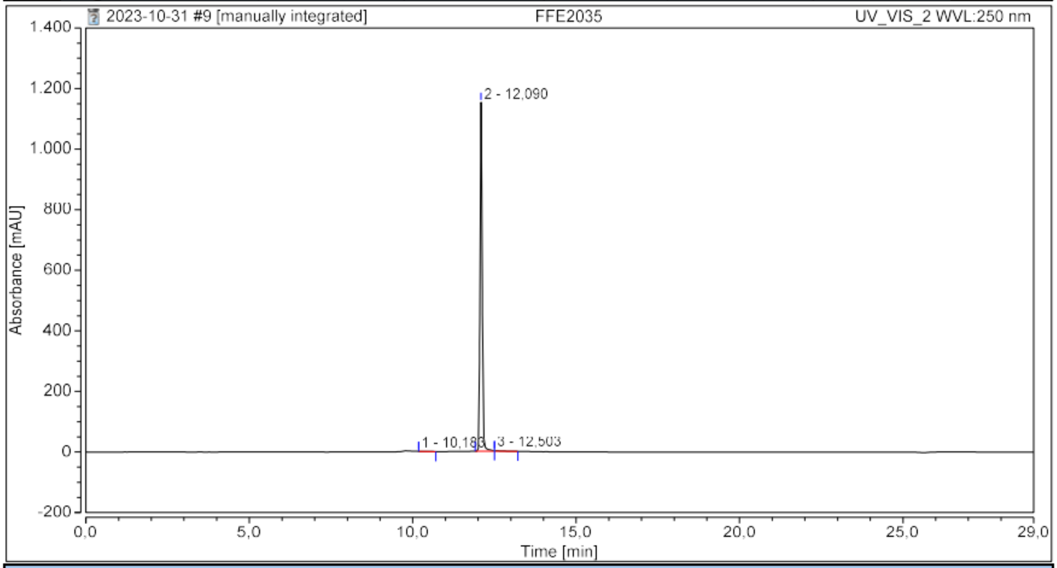

Integration Results

No.	Peak Name	Retention Time min	Area mAU*min	Height mAU	Relative Area %	Relative Height %	Amount n.a.
1		10,183	0,306	0,751	0,34	0,07	n.a.
2		12,090	89,258	1151,844	98,91	99,74	n.a.
3		12,503	0,678	2,252	0,75	0,19	n.a.
**Total:**			**90,242**	**1154,847**	**100,00**	**100,00**	

HPLC chromatogram of **1d** (gradient A, purity: 98.9%).

Chromatogram

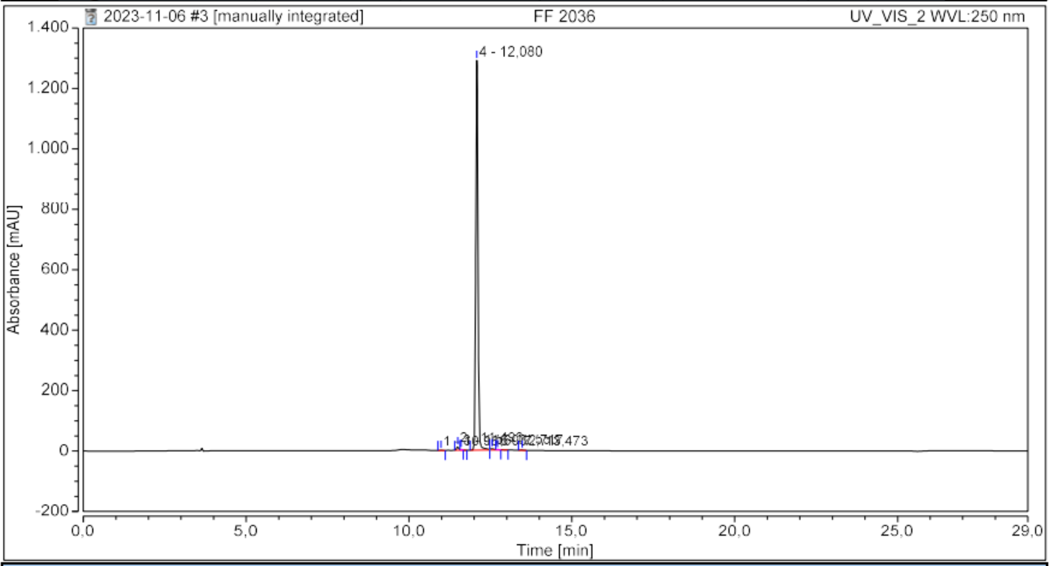

Integration Results

No.	Peak Name	Retention Time min	Area mAU*min	Height mAU	Relative Area %	Relative Height %	Amount n.a.
1		10,980	0,057	0,791	0,06	0,06	n.a.
2		11,493	1,007	12,469	1,00	0,95	n.a.
3		11,603	0,079	1,562	0,08	0,12	n.a.
4		12,080	98,773	1290,418	97,93	98,36	n.a.
5		12,553	0,780	4,846	0,77	0,37	n.a.
6		12,717	0,031	0,508	0,03	0,04	n.a.
7		13,473	0,135	1,315	0,13	0,10	n.a.
**Total:**			**100,862**	**1311,908**	**100,00**	**100,00**	

HPLC chromatogram of **1e** (gradient A, purity: 97.9%).

Chromatogram

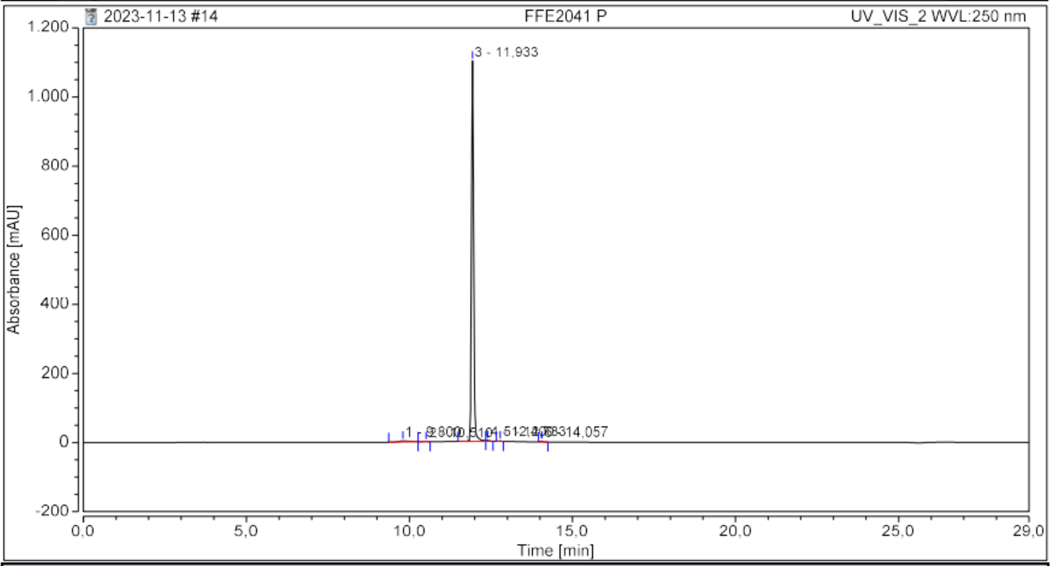

Integration Results

No.	Peak Name	Retention Time min	Area mAU*min	Height mAU	Relative Area %	Relative Height %	Amount n.a.
1		9,800	1,104	3,127	1,28	0,28	n.a.
2		10,510	0,107	0,786	0,12	0,07	n.a.
3		11,933	84,662	1101,043	97,94	99,04	n.a.
4		12,400	0,243	2,365	0,28	0,21	n.a.
5		12,783	0,119	1,693	0,14	0,15	n.a.
6		14,057	0,211	2,711	0,24	0,24	n.a.
**Total:**			**86,446**	**1111,726**	**100,00**	**100,00**	

HPLC chromatogram of **1f** (gradient A, purity: 97.9%).

Chromatogram

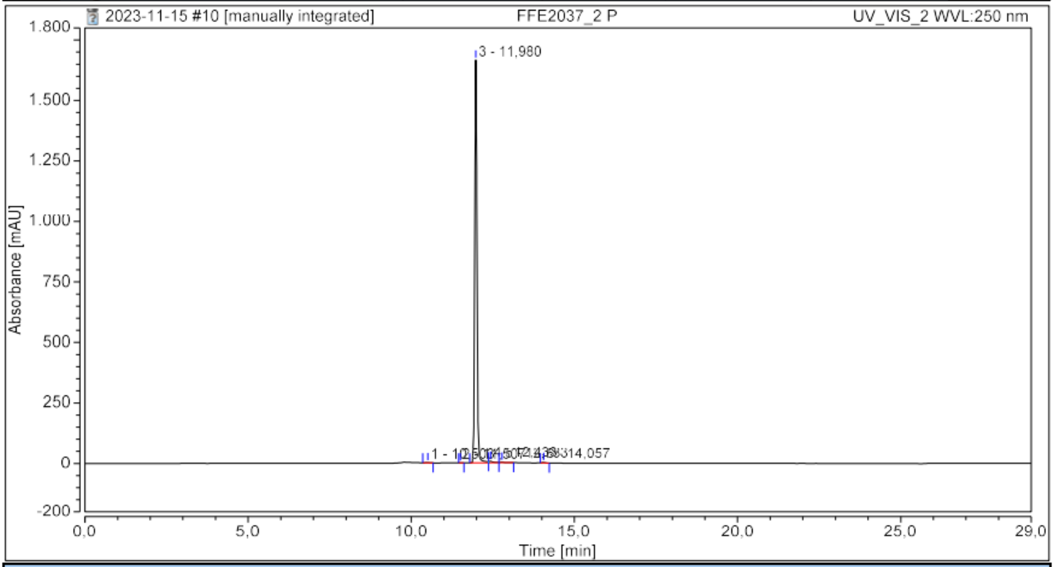

Integration Results

No.	Peak Name	Retention Time<br>min	Area<br>mAU*min	Height<br>mAU	Relative Area<br>%	Relative Height<br>%	Amount<br>n.a.
1		10,503	0,105	0,681	0,08	0,04	n.a.
2		11,507	0,035	0,605	0,03	0,04	n.a.
3		11,980	128,061	1664,337	98,69	99,23	n.a.
4		12,433	0,974	6,297	0,75	0,38	n.a.
5		12,783	0,364	2,427	0,28	0,14	n.a.
6		14,057	0,226	2,881	0,17	0,17	n.a.
**Total:**			**129,766**	**1677,228**	**100,00**	**100,00**	

HPLC chromatogram of **1g** (gradient A, purity: 98.7%).

Chromatogram

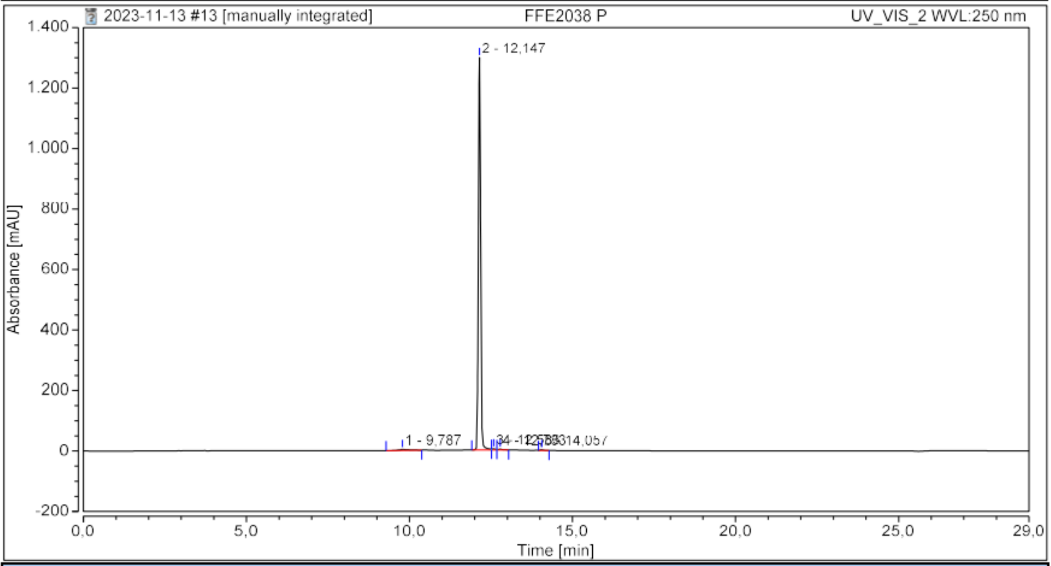

Integration Results

No.	Peak Name	Retention Time<br>min	Area<br>mAU*min	Height<br>mAU	Relative Area<br>%	Relative Height<br>%	Amount<br>n.a.
1		9,787	1,180	3,192	1,13	0,24	n.a.
2		12,147	102,423	1297,680	97,90	99,03	n.a.
3		12,583	0,471	4,115	0,45	0,31	n.a.
4		12,783	0,336	2,733	0,32	0,21	n.a.
5		14,057	0,214	2,701	0,20	0,21	n.a.
**Total:**			**104,623**	**1310,421**	**100,00**	**100,00**	

HPLC chromatogram of **1h** (gradient A, purity: 97.9%).

Chromatogram

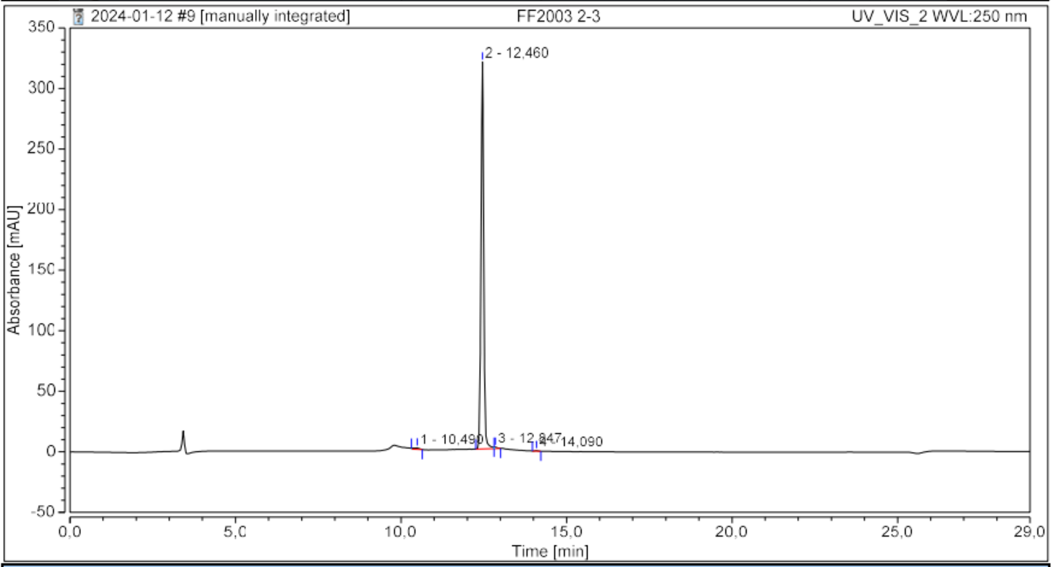

Integration Results

No.	Peak Name	Retention Time min	Area mAU*min	Height mAU	Relative Area %	Relative Height %	Amount n.a.
1		10,490	0,235	1,073	0,75	0,33	n.a.
2		12,460	31,039	319,464	98,74	99,20	n.a.
3		12,847	0,124	1,148	0,39	0,36	n.a.
4		14,090	0,037	0,365	0,12	0,11	n.a.
**Total:**			**31,436**	**322,050**	**100,00**	**100,00**	

HPLC chromatogram of **1i** (gradient A, purity: 98.7%).

Chromatogram

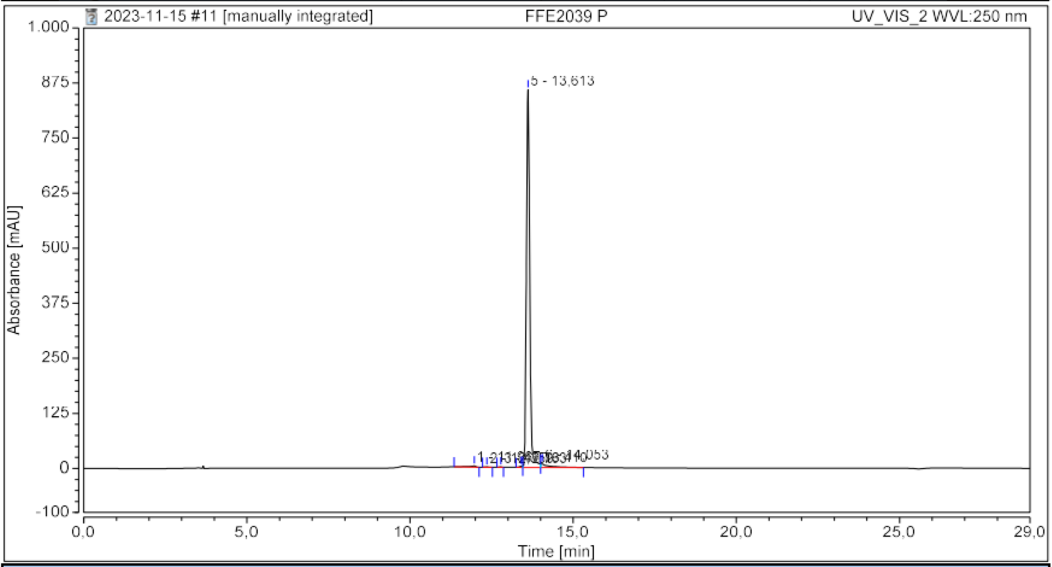

Integration Results

No.	Peak Name	Retention Time min	Area mAU*min	Height mAU	Relative Area %	Relative Height %	Amount n.a.
1		11,967	0,775	2,829	0,75	0,32	n.a.
2		12,350	0,096	0,711	0,09	0,08	n.a.
3		12,783	0,069	0,941	0,07	0,11	n.a.
4		13,410	0,272	2,680	0,26	0,31	n.a.
5		13,613	100,077	857,180	96,47	98,21	n.a.
6		14,053	2,450	8,502	2,36	0,97	n.a.
**Total:**			**103,738**	**872,843**	**100,00**	**100,00**	

HPLC chromatogram of **1j** (gradient A, purity: 96.5%).

Chromatogram

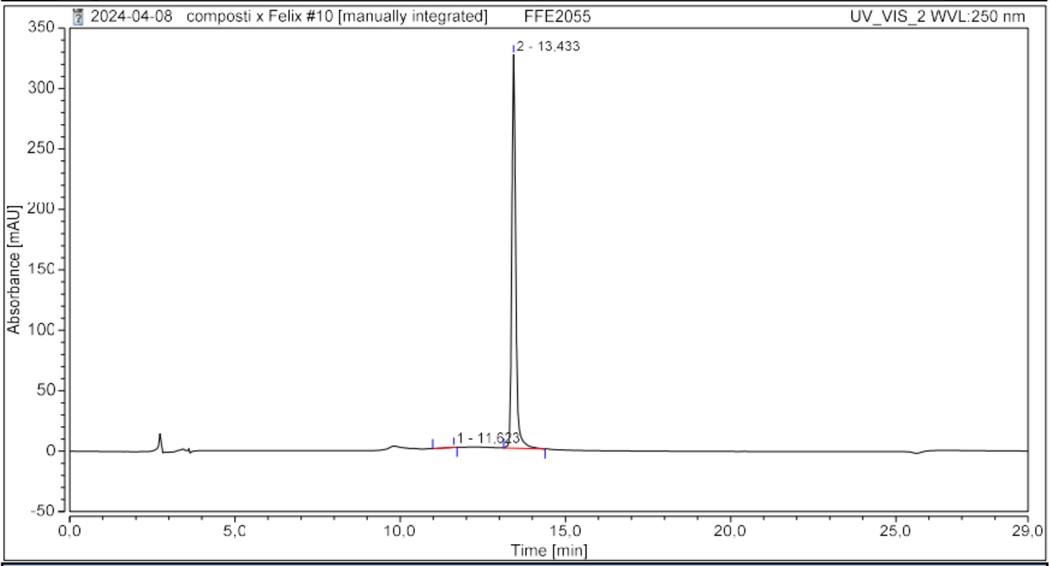

Integration Results

No.	Peak Name	Retention Time min	Area mAU*min	Height mAU	Relative Area %	Relative Height %	Amount n.a.
1		11,623	0,058	0,210	0,13	0,06	n.a.
2		13,433	44,914	325,351	99,87	99,94	n.a.
**Total:**			**44,972**	**325,561**	**100,00**	**100,00**	

HPLC chromatogram of **1j-nc** (gradient A, purity: 99.9%).

Chromatogram

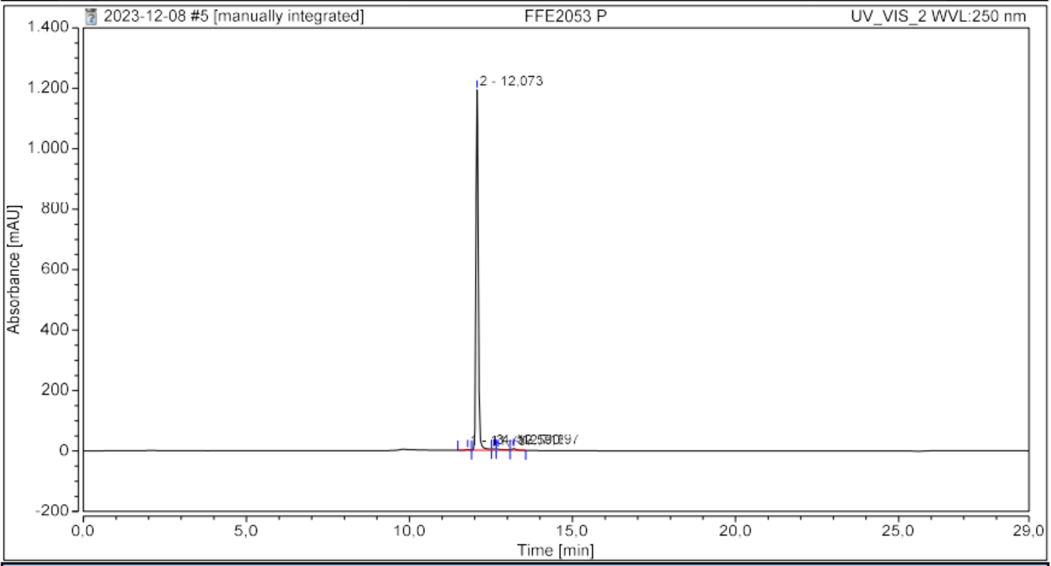

Integration Results

No.	Peak Name	Retention Time min	Area mAU*min	Height mAU	Relative Area %	Relative Height %	Amount n.a.
1		11,787	0,304	2,173	0,31	0,18	n.a.
2		12,073	95,726	1191,763	97,13	98,57	n.a.
3		12,590	0,550	4,756	0,56	0,39	n.a.
4		12,713	1,074	4,347	1,09	0,36	n.a.
5		13,197	0,896	6,060	0,91	0,50	n.a.
**Total:**			**98,550**	**1209,099**	**100,00**	**100,00**	

HPLC chromatogram of **2** (gradient A, purity: 97.1%).

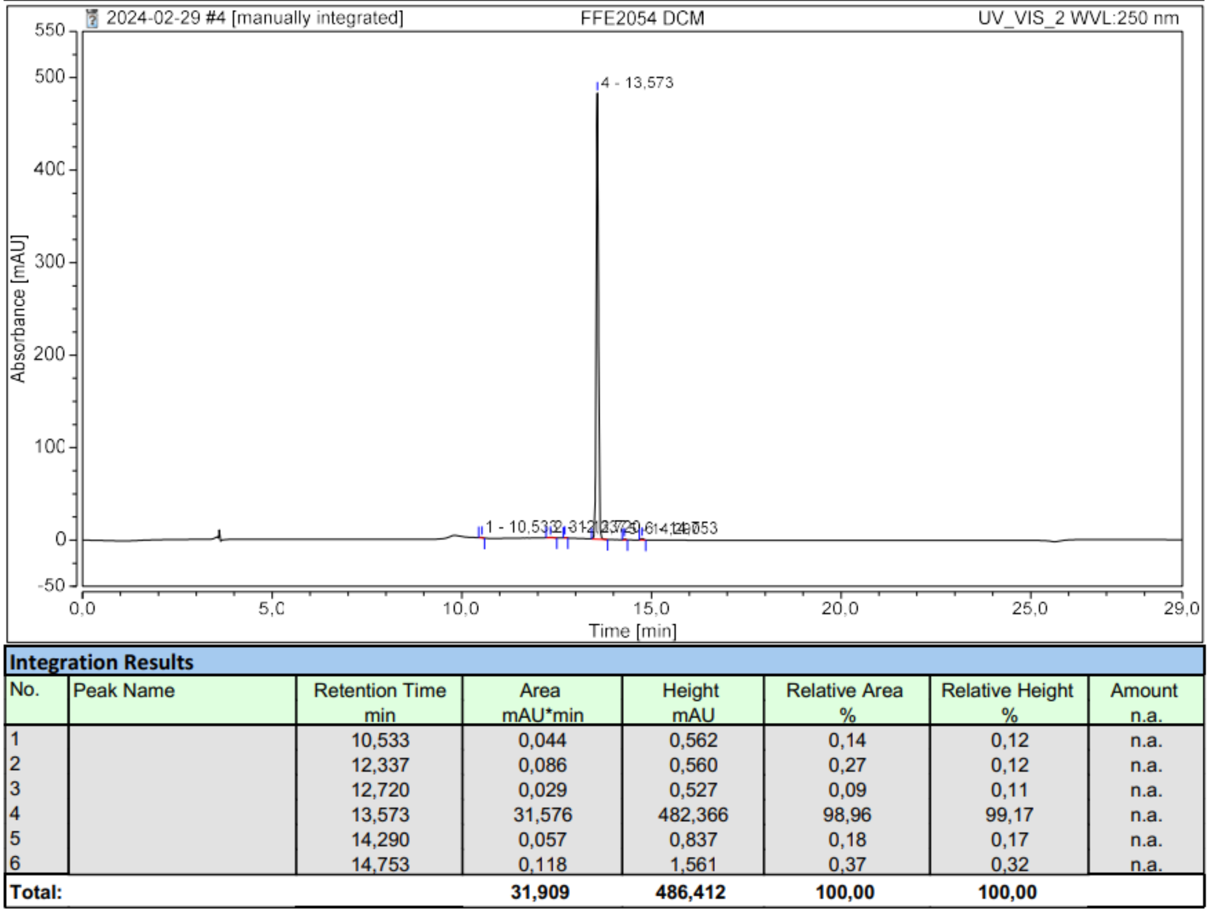

Integration Results

No.	Peak Name	Retention Time min	Area mAU*min	Height mAU	Relative Area %	Relative Height %	Amount n.a.
1		10,533	0,044	0,562	0,14	0,12	n.a.
2		12,337	0,086	0,560	0,27	0,12	n.a.
3		12,720	0,029	0,527	0,09	0,11	n.a.
4		13,573	31,576	482,366	98,96	99,17	n.a.
5		14,290	0,057	0,837	0,18	0,17	n.a.
6		14,753	0,118	1,561	0,37	0,32	n.a.
**Total:**			**31,909**	**486,412**	**100,00**	**100,00**	

HPLC chromatogram of **6** (gradient A, purity: 99.0%).

4. References

[1] T. Ciossek, H. Julius, H. Wieland, T. Maier, T. Beckers, *Anal. Biochem.* **2008**, *372*, 72–81.

[2] C. Bonfils, A. Kalita, M. Dubay, L. L. Siu, M. A. Carducci, G. Reid, R. E. Martell, J. M. Besterman, Z. Li, *Clin. Cancer Res.* **2008**, *14*, 3441–3449.

[3] L. Schäker-Hübner, R. Warstat, H. Ahlert, P. Mishra, F. B. Kraft, J. Schliehe-Diecks, A. Schöler, A. Borkhardt, B. Breit, S. Bhatia, M. Hügle, S. Günther, F. K. Hansen, *J. Med. Chem.* **2021**, *64*, 14620–14646.

[4] N. Reßing, J. Schliehe-Diecks, P. R. Watson, M. Sönnichsen, A. D. Cragin, A. Schöler, J. Yang, L. Schäker-Hübner, A. Borkhardt, D. W. Christianson, S. Bhatia, F. K. Hansen, *J. Med. Chem.* **2022**, *65*, 15457–15472.

[5] F. B. Kraft, M. Hanl, F. Feller, L. Schäker-Hübner, F. K. Hansen, *Pharmaceuticals* **2023**, *16*, 356.

[6] J. V Watson, S. H. Chambers, P. J. Smith, *Cytometry* **1987**, *8*, 1–8.

[7] L. Sinatra, J. J. Bandolik, M. Roatsch, M. Sönnichsen, C. T. Schoeder, A. Hamacher, A. Schöler, A. Borkhardt, J. Meiler, S. Bhatia, M. U. Kassack, F. K. Hansen, *Angew. Chemie Int. Ed.* **2020**, *59*, 22494–22499.

[8] V. Krieger, A. Hamacher, F. Cao, K. Stenzel, C. G. W. Gertzen, L. Schäker-Hübner, T. Kurz, H. Gohlke, F. J. Dekker, M. U. Kassack, F. K. Hansen, *J. Med. Chem.* **2019**, *62*, 11260–11279.

[9] F. Feller, F. K. Hansen, *ACS Med. Chem. Lett.* **2023**, *14*, 1863–1868.
